# Detailed Insight into the Chignolin Folding Process from Maximally Informative Low-Resolution Representations of Its Isocommittor Hypersurfaces

**DOI:** 10.1021/acs.jctc.6c01478

**Published:** 2026-08-26

**Authors:** Alessia Guadagnin Pattaro, Roberto Menichetti, Raffaello Potestio

**Affiliations:** † Physics Department, 19034 University of Trento, via Sommarive 14, I-38123 Trento, Italy; ‡ INFN-TIFPA, Trento Institute for Fundamental Physics and Applications, I-38123 Trento, Italy

## Abstract

The synthetic miniprotein chignolin features a small size, a well-defined native structure, and a rather paradigmatic two-state folding transition. Nonetheless, this deceivingly simple molecule showcases a nontrivial folding pathway, whose detailed characterization has been the subject of several studies and still presents some open questions. In this work, we investigate chignolin by employing, for the first time in a combined and integrated pipeline, transition-path theory (TPT) and the mapping entropy optimization workflow (MEOW). The former describes the folding process in terms of its natural reaction coordinate, that is, the committor; the latter pinpoints, in an unsupervised and system-agnostic manner, those residues of a biomolecule that are most informative about the structural, mechanical, and energetic organization of the configurational ensemble. Through this joint framework, we characterize in great detail the entire transition of the peptide from the unfolded to the native state. The approach allows us to identify which residues entail the largest degree of information about a conformational ensemble at a given level of progress of the folding transition; comparison with data from the literature and validation against independent observables, including the outcomes of an *in silico* mutation analysis, show that these residues also bear functional significance. This work analyzes the folding process of chignolin from a new perspective and showcases the integration of the MEOW protocol with TPT into a pipeline that can complement existing approaches for the investigation of proteins.

## Graphical abstract

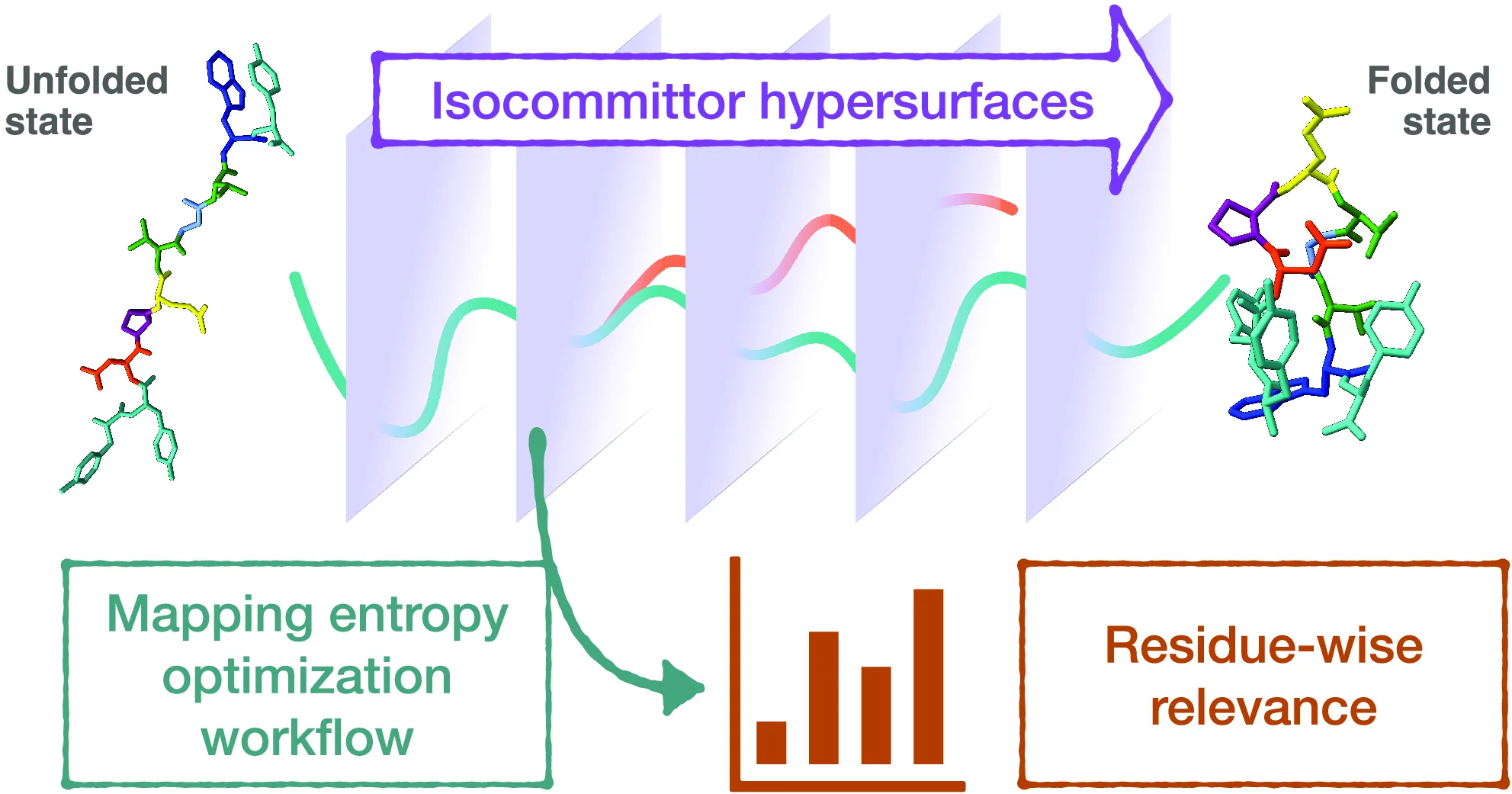

## Introduction

Computer simulations of proteins are key to gaining insight into these molecules at a resolution that cannot be attained in experiments.^1,2^ Despite their importance and usefulness, however, *in silico* approaches are hindered by the accuracy of the force-field, the large size of many molecules of interest, and the long time scales of biological processes such as protein folding, which is extensively investigated by computational means.^3–6^ This notwithstanding, major progress is steadily attained in addressing these limitations, both in terms of the sheer power of the hardware^7–9^ and of the capability of the ever-developing algorithms,^10–12^ especially since artificial intelligence and machine learning entered the game.^13–15^

A particularly effective framework for analyzing the folding mechanism of any protein is that of transition path theory (TPT).^16–19^ TPT enables the characterization of the statistical properties of the ensemble of reactive trajectories that bring a system from a reactant state to a product state; this is done in terms of a function—the *committor*—that quantifies the progress of the transition and represents the ideal reaction coordinate,^16^ in that it completely characterizes the system’s commitment to the transition. The committor is, however, a single scalar parameter in the typically high-dimensional configurational space of the system, and knowledge of it alone (or in combination with other TPT observables) does not guarantee *per se* a mechanistic understanding of the transition at the level of the individual constituents. In particular, a key general problem in characterizing the folding of a protein is to identify which elements—atoms, residues—play a critical functional role, and to figure out if and how these elements change along the transition.

In this work, we address the study of protein folding by means of a novel, general, and system-agnostic analysis protocol. Our approach combines, in a synergistic manner, two hitherto distinct strategies: on the one hand, we make use of TPT to quantitatively characterize the statistical mechanics of the transition and provide a rigorous measure of reaction progress through the committor; on the other hand, we employ the recently developed mapping entropy optimization workflow (MEOW)^20–23^ to assign to each residue a measure of its structural, mechanical, and energetic importance in each ensemble associated with a given degree of the folding progress.

The proposed pipeline is applied to the case of miniprotein chignolin.^24^ This is a small (10 residues) designed polypeptide, which features a well-defined native structure and can rapidly interconvert between its unfolded and folded states, thus rendering the characterization of its conformational transition amenable to molecular dynamics (MD) simulations. Consequently, a large amount of effort has been put into the investigation of chignolin’s folding pathway, revealing, despite the apparent simplicity of this molecule, nontrivial features.^25–37^

Our study of chignolin yielded two main results. First, we found that the group of residues that retains the largest amount of information, or relevance, about the configurational ensembles changes with the level of progress of the folding reaction. Such residues were identified by the protocol in an unsupervised manner and based only on information-theoretic grounds; the *a posteriori* inspection of system-specific observables as well as an *in silico* mutation analysis point toward a functional importance of these amino acids, in line with what was proposed by previous independent studies on the system. Second, by applying our approach in distinct basins of the transition pathway, we characterize different alternative folding mechanisms individually, highlighting the specific features of each one independently.

The strategy employed in this work was tested on miniprotein chignolin as a proof of concept and provided interesting insights into the folding process of this molecule from a novel perspective. The approach showcased here is completely general, and its applicability is not restricted to small proteins; on the contrary, it can fruitfully complement existing analysis tools, contributing to the study of protein folding and the development of effective analysis methods in the context of computational biochemistry.

## Materials and Methods

In this section, we introduce the system under examination, namely, the miniprotein chignolin, recapitulate the frameworks of transition path theory and mapping entropy optimization workflow, and explain their integration into a unified pipeline for the analysis of the chignolin folding process.

### Chignolin and Its Folding Process

Chignolin (Figure 1, left) is a small peptide designed by Honda and co-workers using as a template the B1 chain of the G-peptide, a naturally occurring protein that folds spontaneously into a β-hairpin structure in an aqueous solution.^24^ Experimental probes showed that chignolin is capable of folding in a two-state, cooperative manner and adopts a well-defined native structure, thereby providing empirical grounds for the definition of such a short polypeptide as a protein in its own right. The same research group subsequently optimized the chignolin sequence with the aim of enhancing its stability; specifically, in 2008, Honda et al. reported on a modified variant of this peptide, dubbed CLN025 ^25^ (Figure 1, right), in which the terminal glycines were replaced with tyrosines. Compared to wild-type (WT) chignolin, CLN025 displays increased thermal stability, higher melting temperature, and higher unfolding enthalpy. This variant is the focus of the present work and, from here on out, the term “chignolin” refers to the CLN025 mutant.

**Figure 1:**
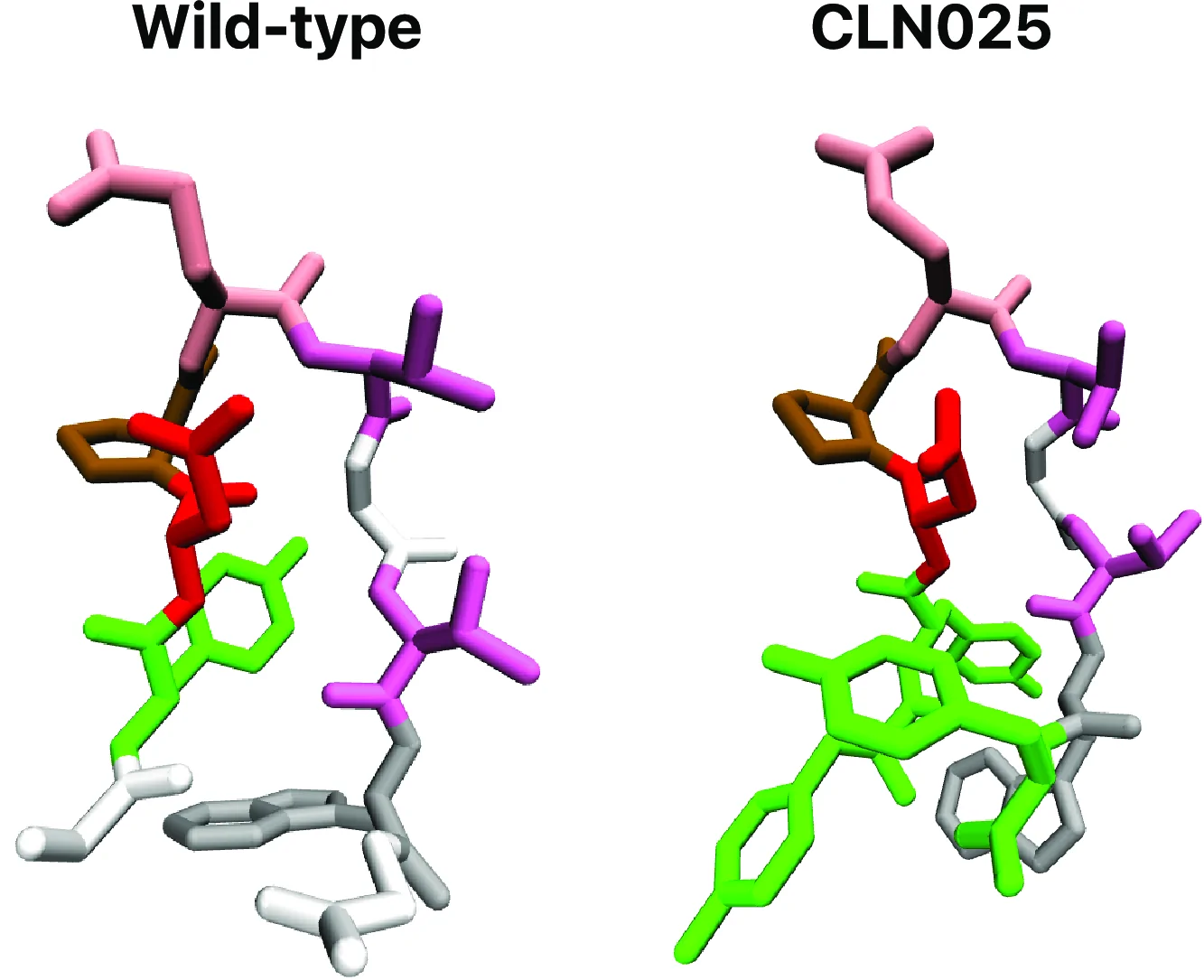
Rendering of the wild type chignolin structure (left) and the derived protein CLN025 (right). Amino acids are colored differently according to their name (Gly in white, Tyr in green, Asp in red, Pro in brown, Glu in pink, Thr in magenta, and Trp in gray).

CLN025 has been extensively investigated experimentally^25,26^ as well as computationally.^27–37^ Several works, starting with the one in which it was introduced,^25^ have sought to characterize its thermodynamic, structural, and kinetic properties. In particular, empirical evidence has been gathered that this molecule is a two-state folder,^25^ with a smooth, funnel-shaped free energy landscape connecting the unfolded basin to a well-defined native state. Various studies have also shown that the transition occurs in the absence of intermediate states and with an extremely fast rate, even exceeding that of other similarly sized two-state folders.^26^

Detailed inspection, however, has revealed that the folding mechanism of chignolin is not as straightforward as its relatively simple architecture may suggest. In fact, early experimental studies^26^ have identified two concurrent, competing folding pathways, with slightly different rates. These pathways involve either the formation of the turn or that of the β-sheet as a key, distinctive trait, and neither of them was found to be a rate-limiting step. In contrast, they occur alternatively and early in the course of the folding, the main “bottleneck” being due to the search for the right fine geometry to establish stabilizing contacts. The picture that emerges from experimental investigation tells of a rapid collapse, largely driven by hydrophobic interactions, and a final stabilization of the structure through the formation of interstrand contacts; in between, a major role is played by the termini, which favor the compactness of the chain (up to the point of taking its folding rate close to that of cyclic peptides^26^), and by the residues in proximity of the turn, which drive the formation of the hairpin bend.

Complementarily, computational methods^27–30,34,35^ have been largely deployed to validate and deepen this picture. A milestone study by Lindorff-Larsen and co-workers^27^ revealed that, in the very early stages of the folding process, CLN025 attains topology and secondary structure close to those of the native state, and only later forms stabilizing contacts. Most interestingly, they observed a rather large fraction (>30%) of native contacts already formed at the transition state. Various authors have subsequently investigated chignolin *in silico*, analyzing the features of its thermodynamics and folding pathway as well as addressing the dependence of the latter on the force field employed in the simulations. In fact, the choice of protein and/or solvent model turned out to impact in a nontrivial manner the outcome of a computer-aided characterization of chignolin.^28,32,34^ This notwithstanding, general consensus is found in the cooperative, two-state folding process of this peptide, as well as in the presence of two distinct routes that the molecule can follow to reach the native state. In these works, different analysis approaches have been employed,^28–30^ most notably sampling strategies based on transition path theory^31–33,36^ that allowed a detailed characterization of the folding by making use of the committor as a natural coordinate to follow the progress of the reaction.

In summary, despite its small size and deceivingly simple native architecture, chignolin appears to *simply behave in a complex manner*, folding through a cooperative, two-step process while at the same time having two alternative ways to do so. While both experimental and computational approaches have greatly contributed to shedding light on this, various aspects still remain unclear. In the following, we describe the workflow that we developed and applied to furthering our understanding of this system and, in perspective, of many others.

### The Methodology at a Glance

The approach presented in this work, and here applied to the investigation of chignolin, aims at identifying those residues that contribute most strongly to the structural and energetic organization of a protein in configurational ensembles characterized by different degrees of progress in the folding reaction. To this end, one has to answer two separate and interconnected questions: first, how can we rigorously and quantitatively parametrize the transition from the unfolded to the folded state? Second, what quantifier can be employed to assess the *importance* of a residue in each of these steps?

The first question can be addressed through transition path theory,^16–19^ a theoretical framework developed to characterize the properties of the ensemble of reactive trajectories of a system connecting a *reactant* state to a *product* state. In our context, the reactant and product correspond to the unfolded and folded basins of the protein, respectively. Each reactive trajectory thus represents a realization of the peptide’s folding process, and TPT investigates such conformational transition from a statistical mechanics perspective.

A pivotal ingredient of TPT is the *committor function q*(**x**),^38,39^ which provides a quantitative description of the progress of the reaction. The committor is the probability (hence *q* ∈ [0, 1]) that a trajectory starting from a given configuration **x** will lead the system to the product state before returning to the reactant. *q* is thus zero in the unfolded state, one in the folded state, and grows monotonically as the system transitions from the former to the latter. In its exact formulation, the committor is the ideal reaction coordinate:^40^ it assigns to each configuration the probability of reaching the product state before returning to the reactant state, and configurations with the same committor value are statistically equivalent with respect to the transition; furthermore, a projection of the transition onto this variable reproduces the exact equilibrium kinetics of the full system. The committor thus provides the optimal one-dimensional coordinate to follow the progress of the reaction; in practical implementations, however, the accuracy of the results depends on the reliability of the force field employed, the coverage of the sampled space, and the parametrization of the committor model.

Central to this work is then the notion of *isocommittor hypersurface* (IH),^38,39^ i.e., the subset of all configurations that share the same committor value. The committor thus allows us to resolve the folding transition into “stages” of reaction progress, to be understood as statistical ensembles of configurations with comparable probabilities of reaching the folded basin before the unfolded one. Analyzing such stages individually allows us to extract information about the structural variability that remains after fixing the degree of progress along the folding transition.

**Figure 2:**
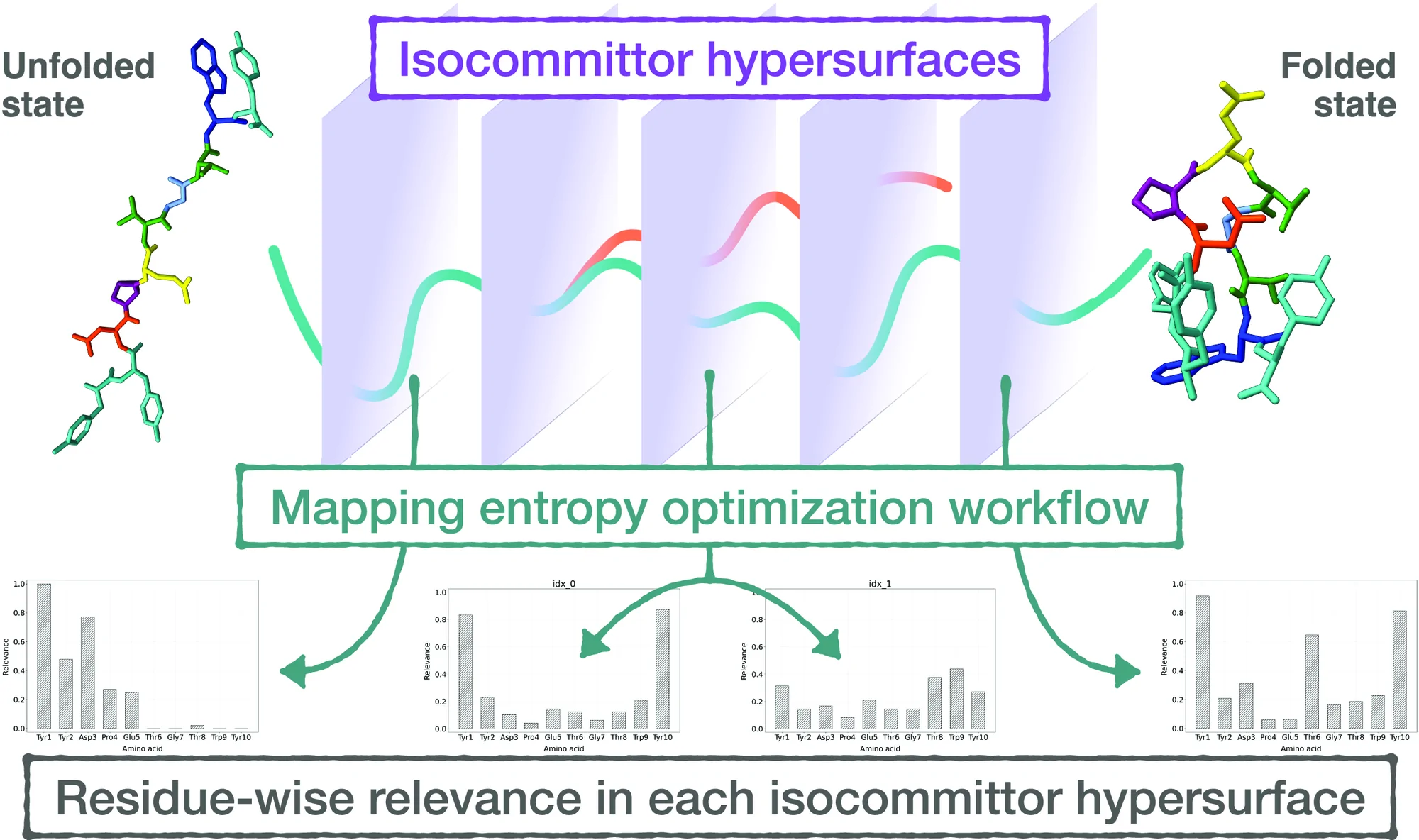
Graphical representation of the TPT+MEOW pipeline employed in this work. The configurations sampled in the course of extensive folding/unfolding trajectories are grouped according to their committor value, thereby producing a foliation of the configuration space between the unfolded and the folded states. Subsequently, the frames in each isocommittor hypersurface are analyzed through the lens of the mapping entropy optimization workflow, which returns a value indicating the structural, energetic, and functional relevance of each residue in the given conformational ensemble. Lastly, if the hypersurface is crossed by more than one reaction pathway, the MEOW analysis can be performed in each of them independently.

This leads us to the second question: what criterion can we employ to define and identify “relevant” residues in a given pool of configurations of the system? A possible solution to the problem is offered by the mapping entropy optimization workflow,^20–22^ a recently developed analysis protocol that assigns to each atom in a molecule a measure of its structural, energetic, and functional importance across the sampled configurations. The method relies on the notion of low-resolution representations of the system at hand, whose information content can be quantified and optimized. More specifically, we define a low-resolution description as one based on a subset of the system’s constituent atoms, or *decimation mapping*, where different mapping choices can be associated with varying degrees of informativeness on the system’s behavior. For example, consider a 10-residue peptide such as the one investigated in this work. Representing it solely in terms of its 10 C_α_ atoms or instead using 10 atoms drawn from its first amino acid significantly affects how much of the system’s properties can be captured. Intuitively, when analyzing a system through reduced descriptions, some subsets of atoms can prove more effective than others.

This qualitative notion is made quantitative by the *mapping entropy* (ME),^20–23,41–44^ the latter being a measure of the statistical information that is lost when a system is looked at in terms of a subset of its original constituents. Critically, the ME only depends on the choice of the retained atoms one employs in the protein’s low-resolution representation, so that a selection that *minimizes* this observable returns the largest amount of information about the configurational space sampled by the molecule, compatibly with the chosen level of detail. In particular, the mappings minimizing the ME include those atoms whose positions are determinant to define the configurational and energetic state of the whole protein. By identifying several selections that minimize the ME, we can assign to each atom a probability to be part of an optimal low-resolution representation, which is then employed as a measure of that atom’s relevance in dictating the statistical behavior of the system.^20–22^

The protocol of determining, for a generic system, its maximally informative reduced representations through which calculating the associated atom-wise probabilities goes under the name of mapping entropy optimization workflow.^1,20–23,45^ Importantly, previous applications of MEOW to biological macromolecules^20–22^ revealed that the method is capable of identifying, in an unsupervised manner, functional regions of the system as catalytic or binding sites, further capturing how their relevance can depend on the specific conformational basin considered. It is natural to expect, however, that the importance of a particular residue of a protein undergoing, e.g., a conformational change may vary *in different stages of the process*; motivated by this consideration, we here combine MEOW and TPT in a unified framework and apply this scheme to the analysis of the chignolin folding transition. In particular, the configurational space of the reaction is foliated into IHs to which MEOW is separately applied; by conditioning configurations on the committor, we factor out differences in the *degree of commitment to folding*. The relevance obtained from MEOW in each IH therefore allows us to pinpoint which residues optimally describe the conformational variability among structures sharing the same probability of reaching the folded state. Tracking this quantity across successive committor values reveals how the statistical properties of the transition-path ensemble evolve along the reaction. The pipeline here described is schematically illustrated in Figure 2.

### Transition Path Theory

The first component of our integrated workflow, transition path theory,^16–19^ provides a mathematical framework for analyzing the ensemble of reactive trajectories of a system that connect a reactant and a product state. The approach is very general, and in the context of protein biophysics it can be employed for the quantitative study of different kinds of reactions, e.g., conformational changes, allosteric transitions, and protein folding. In this latter case, the unfolded basin is identified with the reactant state, and the native basin with the product state. Hereafter, we provide a brief illustration of TPT, referring the interested reader to the associated literature for further technical details.^16–19,38–40,46^

We consider a system whose configuration **x**(*t*) at time *t* belongs to set $\Omega \subseteq R^{D}$ and whose evolution is dictated by a stochastic, Markovian dynamics satisfying detailed balance with respect to an equilibrium probability distribution *m*(**x**). The system is further assumed to be ergodic with respect to *m*(**x**), so that given an infinitely long trajectory ${\left\{x\left(t\right)\right\}}_{t\in R}$ and an observable *O*(**x**), one has

$$\underset{T\to \infty }{\lim}\frac{1}{2T}\int_{-T}^{T}d\mathrm{t O}\left(x\left(t\right)\right)=\int_{\Omega }dx\;O\left(x\right)m\left(x\right)$$ 1

In the case of a system in the canonical ensemble, *m*(**x**) corresponds to the Boltzmann distribution exp­(−β*U*(**x**))/*Z*, where *U*(**x**) is the potential energy, and *Z* is the corresponding canonical configurational partition function.

We now want to focus our attention on a reactive process occurring in the system. Accordingly, within Ω, we define two subsets *A* and *B* representing the reactant and product states, respectively, with *A* ∩ *B* = ⌀. The reactive region is then defined as R=Ω\(A∪B) , which thus consists of all the system’s configurations that lie outside the reactant and product basins. The infinitely long ergodic trajectory ${\left\{x\left(t\right)\right\}}_{t\in R}$ ensures the observation of a large (potentially infinite) number of transitions between the reactant set *A* and the product set *B*; from these transitions, *reactive fragments* of the original trajectory can be extracted as those portions of ${\left\{x\left(t\right)\right\}}_{t\in R}$ that are contained in the reactive region R and strictly originate from A and terminate in B—a pictorial representation of this concept being provided in Figure 3. TPT focuses on such transition path ensemble to quantitatively characterize the statistical properties of the reaction.

**Figure 3:**
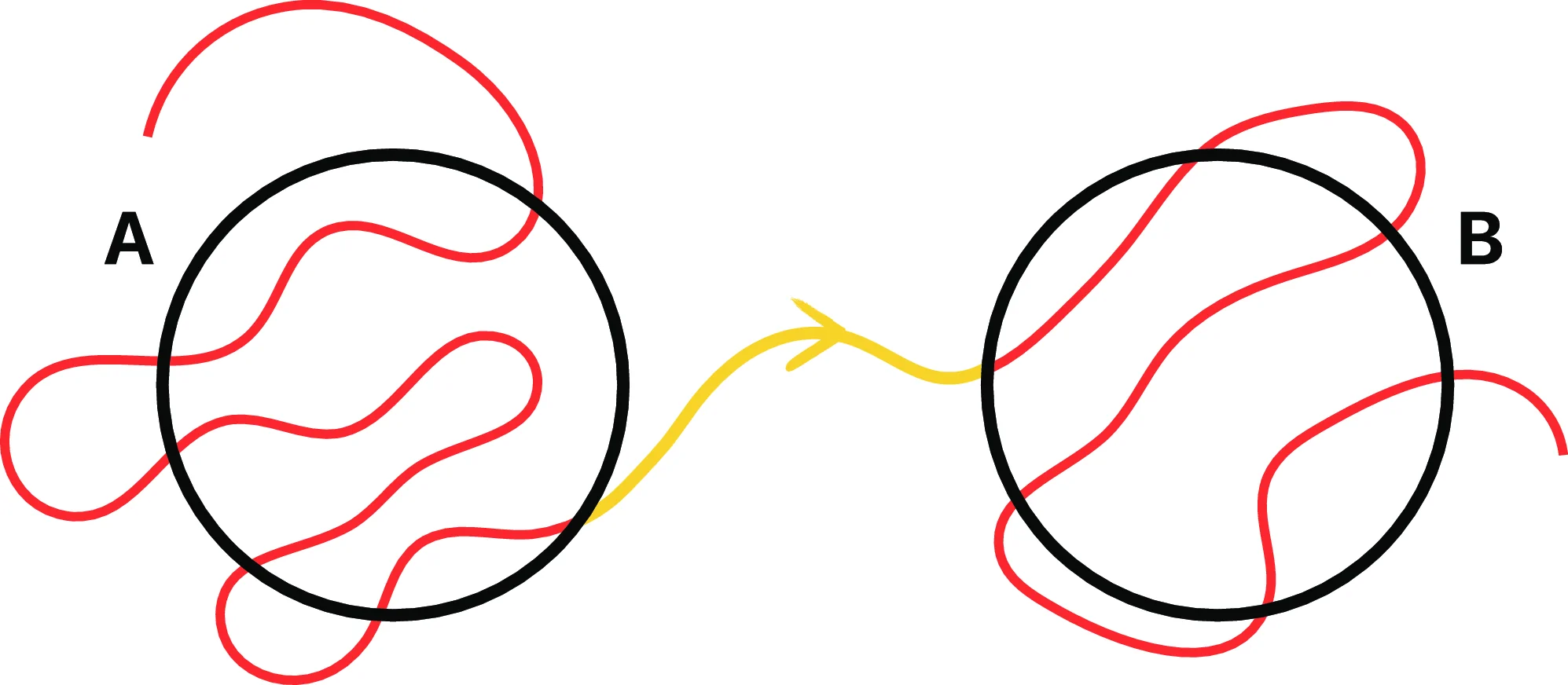
Graphical representation of the reactant state *A* and the product state *B*, crossed by a trajectory (in red). The reactive part of the trajectory is highlighted in yellow; the combination of all reactive trajectories constitutes the transition path ensemble.

Within TPT, the transition from the reactant to the product can be fruitfully analyzed in terms of the *committor function q*(**x**), defined as the probability that a trajectory starting from configuration **x** reaches the product state *B* before the reactant state *A*.^16,18,38–40,46^ Given the committor, the probability density of configurations **x** crossed by reactive trajectories is provided by

$$\begin{array}{l} m_{\mathrm{AB}}\left(x\right)=q\left(x\right)\left(1-q\left(x\right)\right)\frac{e^{-\beta U(x)}}{Z_{\mathrm{AB}}}\\ Z_{\mathrm{AB}}=\int_{R}dx\;q\left(x\right)\left(1-q\left(x\right)\right)e^{-\beta U(x)} \end{array}$$ 2

Furthermore, the reactive space can be partitioned into regions where the committor function attains a well-defined value. On such *isocommittor hypersurfaces* (IHs), the probability density of configurations belonging to a reactive trajectory satisfies

$$m_{q}\left(x\right)=\frac{e^{-\beta U(x)}}{Z_{\mathrm{AB}}}q\left(x\right)\left(1-q\left(x\right)\right)\delta \left(q\left(x\right)-q\right)$$ 3

and is thus proportional to the equilibrium probability distribution, i.e., the Boltzmann weight. This property of IHs is relevant for the application of the MEOW approach, which relies on the assumption that the configuration sample under examination is Boltzmann-distributed.^20,21^

In this work, our sample consists of four independent unbiased MD trajectories of chignolin that we combine in a single trajectory totaling ∼120 μs,^31^ made available by the group of R. Covino, which were processed to partition the configurations according to their committor value—see Supporting information (SI) for all technical details. We relied on the committor function model developed by Covino and co-workers,^31,47^ where a neural network is employed to control and increase the sampling of reactive transition paths while concurrently learning the committor.

The committor range [0, 1] was partitioned into 20 equally sized intervals, and we assigned each configuration to the IH in which its committor value falls. In the upper panel of Figure 4, we report the number of configurations sampled in each bin associated with a given range of committor values, with the counts reported on a logarithmic scale to highlight the less populated bins. Conformations in the proximity of the folded state were found to be the most frequently visited, followed by those assigned to the unfolded basin. The bottom panel of the same figure shows the associated free energy profile as a function of the committor.

**Figure 4:**
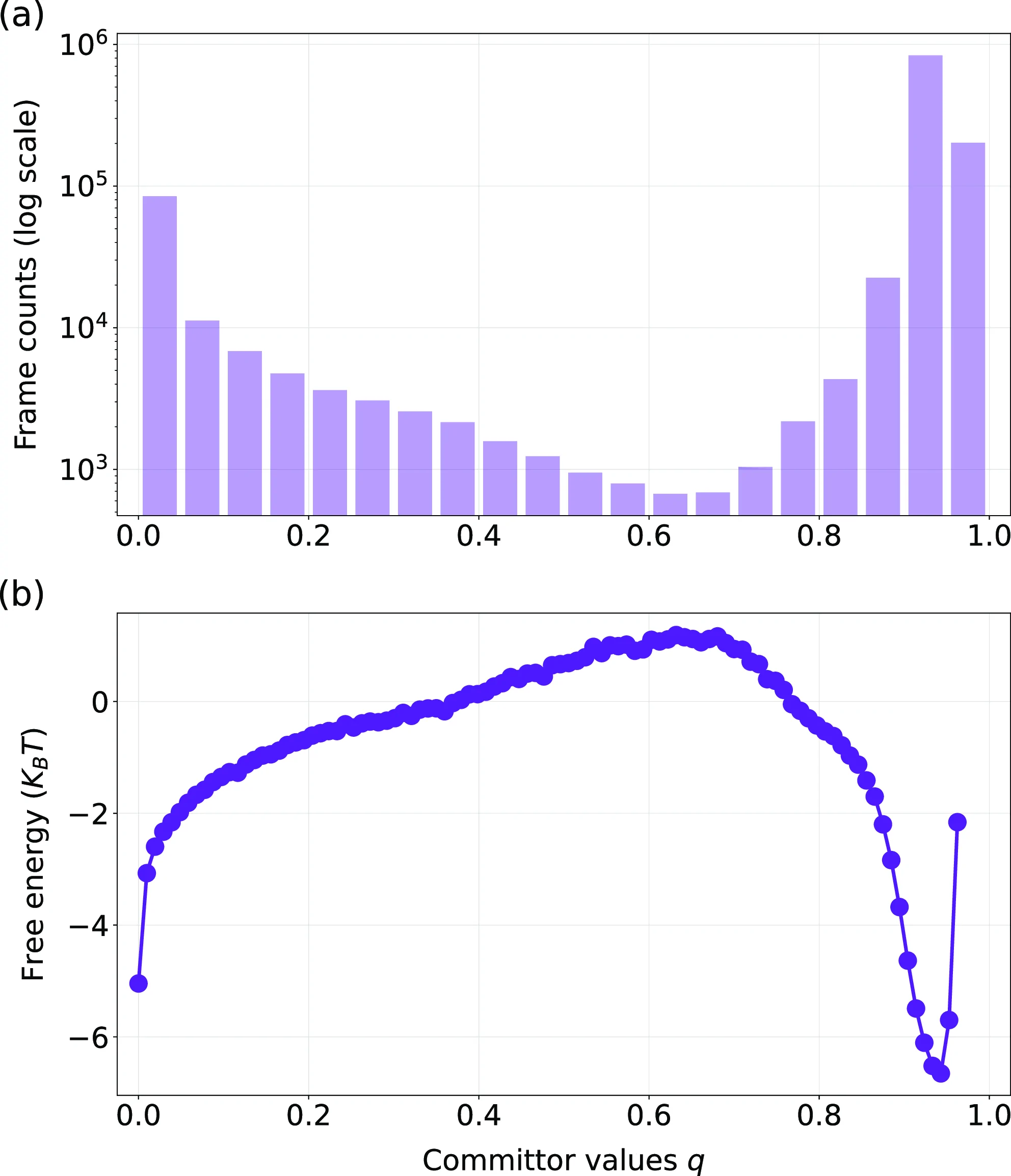
(a) Occurrence of committor function values for the full, ∼120 μs-long MD trajectory of chignolin. The histogram shows the number of configurations sampled for each range of values of the committor, the latter being partitioned in 20 bins, or *isocommittor hypersurfaces*. The number of frames is reported in a logarithmic scale. (b) Free energy of the system as a function of the committor for the folding process in *K*_B_ *T* units. In deriving panels (a) and (b), and throughout the remainder of this work, we relied on the MD simulations and committor model for chignolin reported by Covino and co-workers.^31,47^

### Mapping Entropy Optimization Workflow

The second ingredient of our integrated pipeline, dubbed mapping entropy optimization workflow,^20–23^ is an information-theoretic method that processes a sample of molecular configurations and returns a measure of structural and energetic relevance of each atom. It builds on the assumption that, when a system is observed at “low resolution”, the loss of detail can be balanced by an enhancement of the important features.

MEOW relies on the definition of reduced representations of the system of interest, which is described in terms of *a subset* of its original constituents: in the case of a protein, instead of keeping track of all atoms in the analysis, we only focus on some of them. The selection of atoms from the high-resolution representation is called a *decimation mapping*,^20,21,48^ and is formally defined in terms of a projection operator **M**(**r**) applied to the Cartesian coordinates of the system **r** = {**r**_1_, **r**_2_,..., **r**_*n*_} to obtain the coarser configuration **R** = {**R**_1_, **R**_2_,..., **R**_*N*_} constituted by *N* < *n* atoms. In particular, for a given mapping **M**(**r**) we have

4 $$\begin{array}{l} M_{I}\left(r\right)=\sigma_{i}r_{i},,\sigma_{i}=1\;\mathrm{for}\;a\;\mathrm{given}\;I,0\;\mathrm{otherwise};\\ \sum_{i=1}^{n}\sigma_{i}=N \end{array}$$

where *i* is the atomic index, and *I* is the index of those specific atoms that are retained in the reduced subset.

Critically, it is possible to compute, for each of such decimation mappings, the amount of *conformational information it loses*, that is, measuring how much structural and energetic variability of the original sample is washed away upon filtering. This measure can be done through the *mapping entropy* (ME), which is defined as^20–23,41–44^

$$S_{\mathrm{map}}=k_{B}\int dr\;p_{r}\left(r\right)\ln\left[\frac{p_{r}\left(r\right)}{{\mathrm{p̅}}_{r}\left(r\right)}\right]$$ 5

In eq 5, *p*_*r*_(**r**) is the probability distribution of the full system taken as a reference. In contrast, *p̅*_*r*_(**r**) is the average probability of all configurations **r** that map onto the same coarse configuration **R**, which is assigned back to the original configuration **r**. The latter distribution thus represents a “smeared” version of the high-resolution probability density. *S*_map_ is a multibody function of the atoms selected to be retained in mapping **M**; the goal of the MEOW method is to identify those mappings that *minimize* the ME, so as to identify subsets of atoms that return a representation of the system entailing the largest degree of statistical informativeness.^1,20–23,45^ Since a single minimization of ME returns only one of its potentially many local minima, several optimizations are typically performed, from which the probability *P*_*i*_ that an atom belongs to an optimal mapping is computed. The probability *P*_*i*_ is thus a measure of how important, or *relevant*, an atom is in constructing an informative low-resolution representation of the system. Eventually, given the atom-wise relevance, we define the relevance of an amino acid as that of its most frequently conserved atom; that is, for residue θ, we have

6 $$P_{\theta }=\max_{i\in \theta }\left(P_{i}\right)$$

Various works highlighted that those residues bearing a high *P*_θ_ are likely involved in key functions of the system under examination. Indeed, MEOW was applied in different contexts, such as simple neural networks, biomolecular systems, and financial markets;^20,22,23,45^ in all cases, it was observed how the elements preferentially retained by the protocol, and identified by it in an unsupervised manner, shed light on the structure and the energetics of the system. Here, the MEOW approach has been employed, in combination with TPT, to identify residues endowed with important structural, energetic, and possibly functional properties in different stages of the folding transition of chignolin. In this work, mapping entropy optimizations were performed through the recently developed EXCOGITO software platform,^21^ see SI for all technical details, which efficiently implements the MEOW approach as well as other mapping-related analysis methods.

## Results and Discussion

The aim of our work is to characterize the folding transition of chignolin in terms of those amino acids that take a prominent part in distinct stages of the process. To this end, we first employ MEOW to investigate the system’s ∼120 μs simulation as a whole and the folded and unfolded basins separately; second, we compute the amino acid relevance profile in each of the 20 isocommittor hypersurfaces in which the folding transition has been partitioned. The choice of subdividing the committor range in 20 IHs represents an optimal balance between slicing the committor range in enough bins to observe a smooth transition, and having a statistically relevant large number of configurations in each bin. The consistency of the obtained results was assessed through the same analysis performed on the partition of the committor range in 10 and in 30 bins: the corresponding outcomes are reported in Figure S2 of the Supporting Information.

### MEOW Analysis of the Full Trajectory and the Folded/Unfolded States

The first step of our study is the MEOW analysis of the full ∼120 μs MD simulation trajectory of chignolin folding/unfolding of ref 31. The combined trajectory, originally constituted of four independent runs of slightly different duration (see SI), was partitioned into four parts of equal length, with MEOW applied separately to each one in order to have independent analysis outcomes and make an estimate of the statistical significance of the results. Figure 5 shows that the amino acid relevance returned by the protocol has a similar trend in all four subtrajectories, with the highest values found for the Tyr1 and Tyr10 terminals, as well as for Tyr2 and Asp3. The substantial agreement among the relevance profiles of the four subsequent chunks, despite the whole 120 μs trajectory being characterized by an inhomogeneous sampling of folded, unfolded, and intermediate conformations, *vide infra*, supports the robustness of the results.

**Figure 5:**
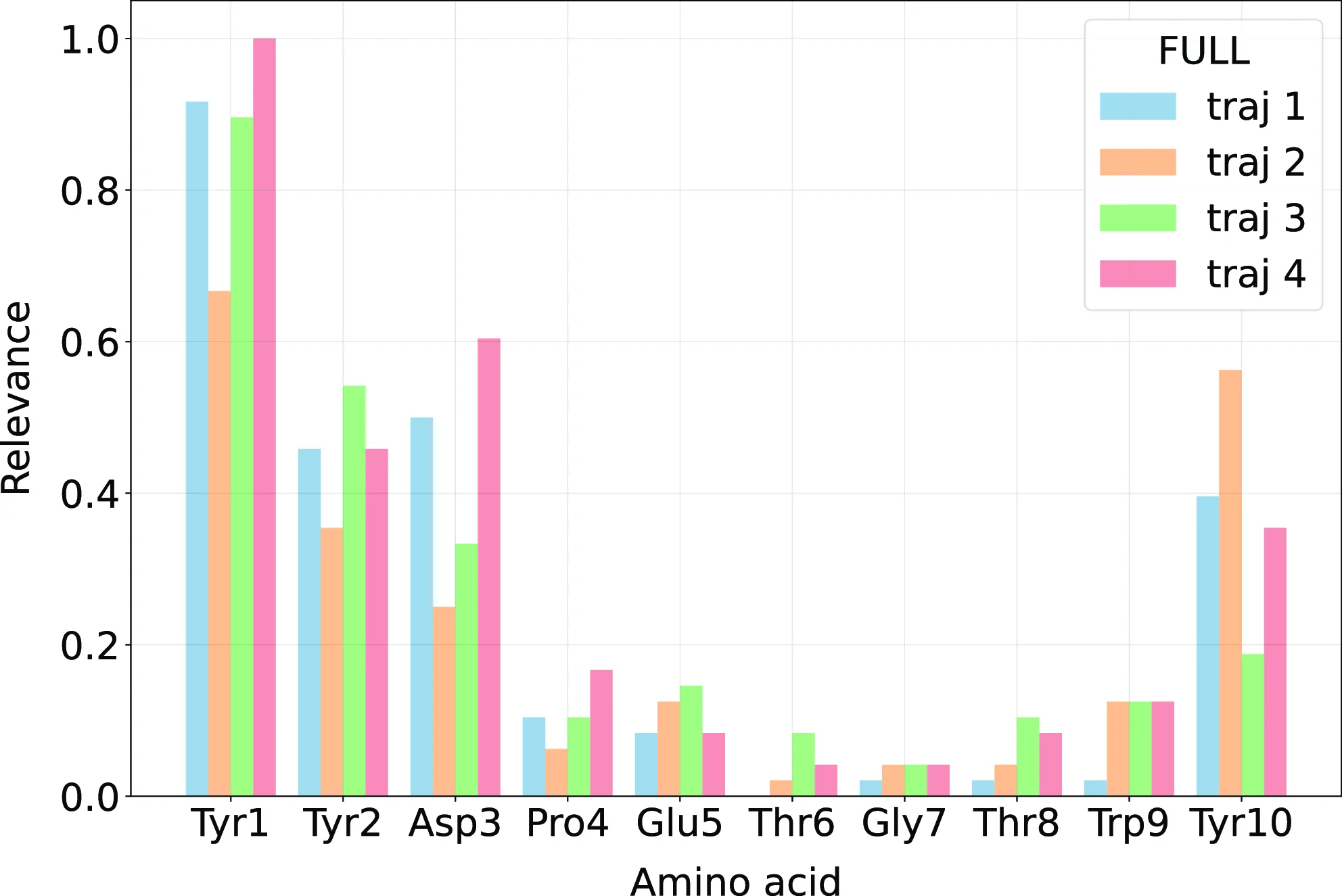
MEOW relevance of each residue in the whole chignolin folding/unfolding transition computed independently on the four chunks in which the full ∼120 μs MD simulation trajectory was partitioned. Results from each chunk are shown in different colors.

According to the analysis of the root-mean-square fluctuation (RMSF) pattern (Figure S1), the two termini are the most fluctuating residues of the peptide: since, in general, amino acids that undergo important conformational changes can be related to large energetic variations, it is reasonable that the termini showcase a high relevance.^20,21^ In contrast, given that Tyr2 and Asp3 do not fluctuate as much as Tyr1 and Tyr10, see Figure S1, the reason for their high relevance must lie elsewhere, as will be discussed more thoroughly hereafter.

We then turned our attention to those amino acids characterized by the highest MEOW relevance in the initial and final states of the process, namely, the peptide’s unfolded, denatured state, and folded, native state. To discriminate the two conformational basins, we relied on a criterion reported by Lazzeri et al.^31^ and discussed in Section S5 of the SI, which employs the fraction of native contacts (FONC) to select only those configurations that are closest to the folded or to the unfolded state. On the basis of this criterion, for both basins we extracted 10 subsamples of 10,000 frames from the original trajectory and applied MEOW independently to each of them. The results obtained are presented in the two panels of Figure 6, where we report, separately for the native and unfolded states, the average relevance of the protein residues calculated across the 10 associated subsamples.

**Figure 6:**
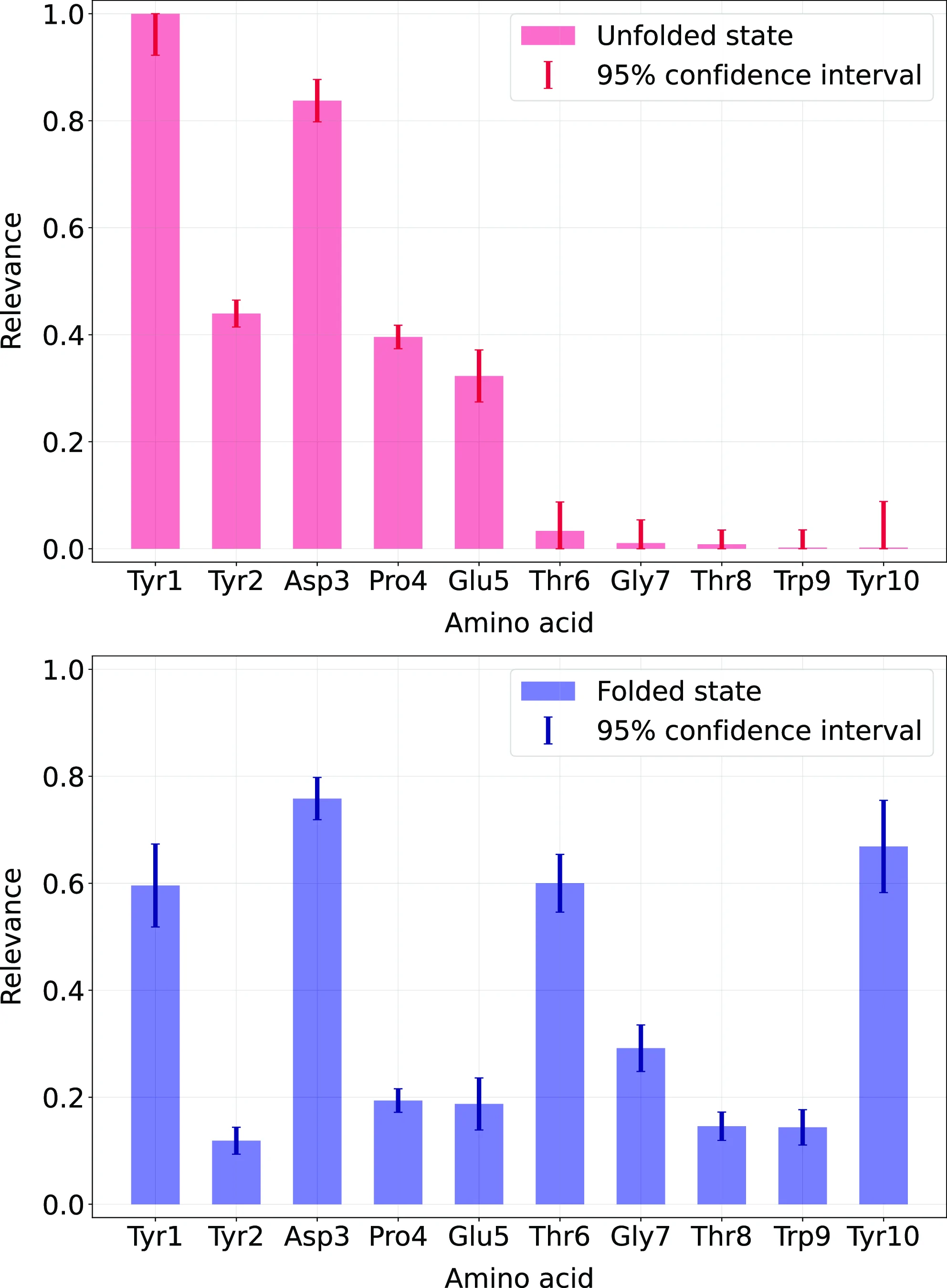
Average MEOW residue relevance calculated from 10 subsamples of the original trajectory restricted to the unfolded (upper panel) and folded (lower panel) states of chignolin. For each state, a confidence interval error of 95%, obtained from the 10 associated subsamples, is shown as an error bar. Only values in [0, 1] are reported.

The outcome of the MEOW analysis on both the unfolded and folded states of chignolin (Figure 6) differs from that which emerged from the processing of the full trajectory (Figure 5). More specifically, in the peptide’s unfolded basin (Figure 6, top panel), Tyr1 and Asp3 display even higher relevance than they do in the full trajectory; interestingly, the interaction between these residues was reported by Sobieraj and Setny^30^ to be an important structural element for chignolin in its path from the unfolded to the transition state, compatible with the behavior observed in Figures 5 and 6. In the full trajectory, though, the inclusion of the intermediate and folded states, as seen in Figure 5, likely lowers the overall relevance of these two residues. Furthermore, Pro4 and Glu5 have a higher relevance in the unfolded basin than they do when the whole trajectory is analyzed, while the subsequent residues along the peptide sequence, most notably Tyr10, lose almost completely any signal in the unfolded state.

We then observe that the MEOW relevance profile of the native state of chignolin (Figure 6, bottom panel) is remarkably different from that of its unfolded and full-trajectory counterparts, the most frequently retained amino acids being Tyr1, Asp3, Thr6, and Tyr10. Notably, Asp3 and Thr6 are the two residues that form the strongest and most numerous hydrogen bonds in the folded protein, as reported by several experiments and simulations.^25,30,33^ Asp3 maintains, also in this basin, a very high relevance of ∼0.8; in contrast, Tyr1 drops from ∼1 in the unfolded state to 0.6 in the folded state. This points to the strength and importance of Asp3 in creating H-bonds and underpins its critical role in the close-to-native chignolin structure, compared to Tyr1. Moreover, Asp3 and Tyr10 form a strong H-bond in the native state, as evidenced by nuclear magnetic resonance experiments.^25^

This initial application of the MEOW protocol thus returns indications about those residues that preserve the largest amount of information about the behavior of chignolin and, prospectively, that entail some functional importance in different conformational basins. To corroborate this hypothesis, we carried out a simple but informative study: specifically, we performed a principal component analysis (PCA)^49^ of all amino acids of the molecule considering separately the full trajectory, the unfolded state, and the folded state; furthermore, a PCA of the three most relevant amino acids identified by MEOW in each state was made, together with a similar analysis in which a 10-fold pool of three randomly selected amino acids was considered. In all cases, the covariance matrices employed to perform the PCA were constructed by using the positions of the α carbons only. The results of this study are presented in Figure 7 and summarized in Table 1.

**Figure 7:**
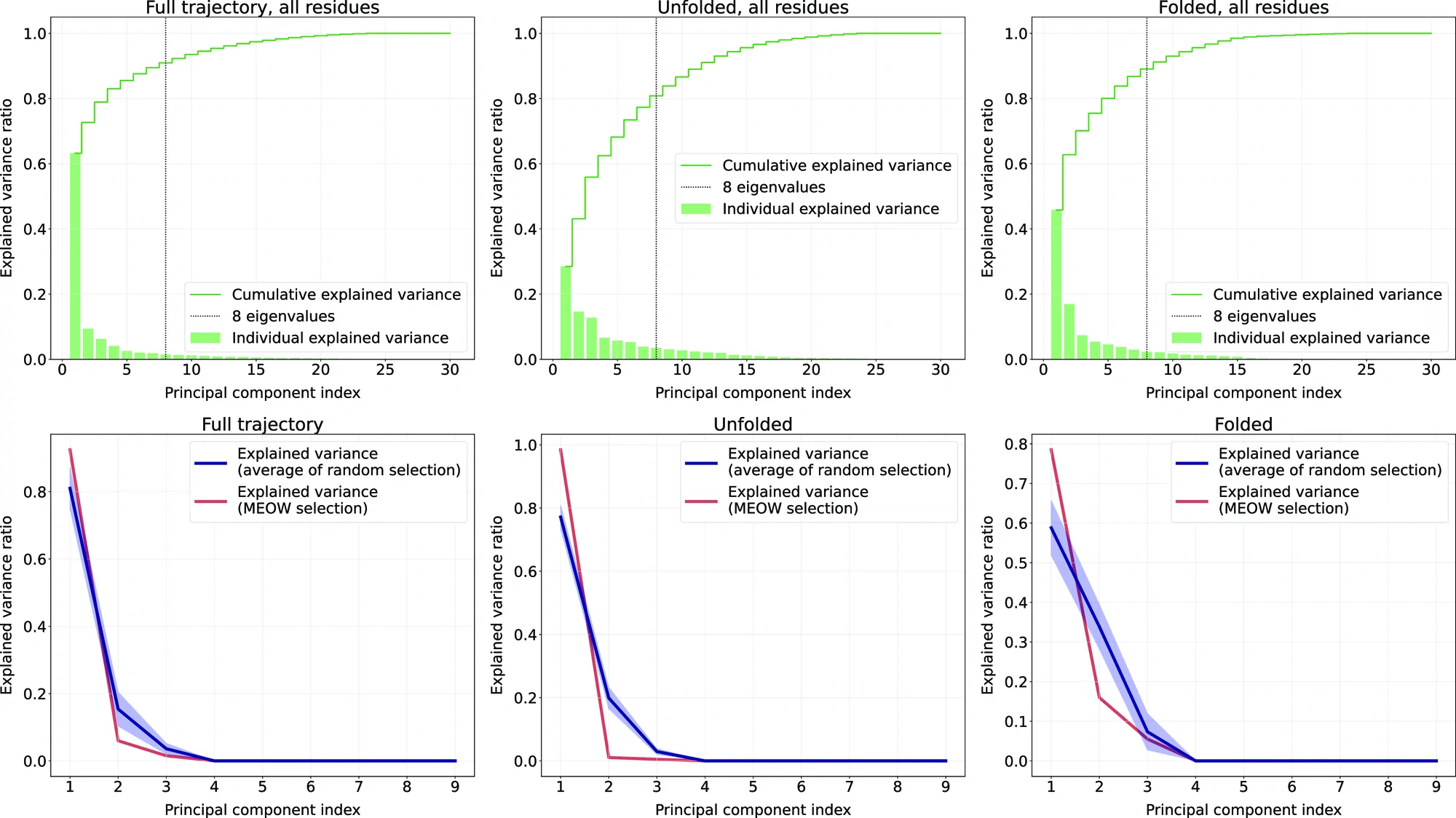
Principal components analysis of the full trajectory (first column), of the unfolded state (second column), and of the folded state (third column) of chignolin. The first row pertains to the structure with all amino acids, showing the individual variance (histogram), the cumulated variance (step function), and the indication of the eighth eigenvalue (vertical bar). The second row reports the results for the three most relevant residues identified by MEOW for each conformational ensemble, where the individual variance over such residues is compared with the average of the variance calculated across 10 random triplets of amino acids with a confidence interval of 95% (blue shadow).

**Table 1:** Cumulated Variance Values for the Full, the Unfolded, and the Folded Trajectories Divided into All Residues (Reporting Cumulated Variance up to the Third and Eighth Eigenvalues), Most Relevant Residues According to MEOW (Reporting Only the Variance for the First Eigenvalue), and the 10 Random Triplets (Reporting the Average Variance for the First Eigenvalue)

	ALL RESIDUES		MOST RELEVANT	10 RANDOM TRIPLETS
	cumul var. (3 eigv)	cumul var. (8 eigv)	cumul var. (1st eigv)	avg cumul var. (1st eigv)
Full traj	0.789	0.910	0.925	0.809
Unfolded	0.559	0.808	0.984	0.745
Folded	0.701	0.891	0.785	0.597

We first analyzed the eigenvalues of the PCA of the whole protein and the cumulative variance as a function of the number of principal components included. From the inspection of Figure 7 (first row), we note that the first eigenvalue is sufficient to account for more than 60% of the variance of the full trajectory, while this value drops to ∼30 and ∼ 45% for the unfolded and the folded state, respectively. This is reasonable, since the full trajectory features a major rearrangement of the molecule and the first principal component describes such a relevant transition from the unfolded to the folded state. In contrast, the unfolded state is largely disordered, suggesting that no well-defined direction in configuration space can be identified, thus leading to a smooth decay of the eigenvalues; the folded state shows an intermediate behavior between the previous two, with the first eigenvalue describing almost half of the covariance. When performing the analysis of the modes describing the conformations of the three most relevant residues identified by MEOW, we note a remarkable boost in the first eigenvalue in all (sub-) trajectories; these reach values as high as 0.93 and 0.98 for the full and unfolded trajectories, respectively, and 0.79 for the folded state. The other eigenvalues are dramatically lower, suggesting that the three residues pinpointed by the MEOW protocol sample the conformational space in a nontrivial and informative manner.

However, a fair comparison of these numbers with those obtained in the analysis of the system in terms of all residues is unfair, since the *total* number of degrees of freedom is different. We thus resort to a simple heuristic: if we consider the C_α_ atoms only, the full structure will have 10 atoms, making up a total of 30 degrees of freedom, 6 of which are associated with null roto-translational modes; hence, the effective, nontrivial covariance modes are 24. Differently, if we have three α carbons, we will obtain a total of 9 degrees of freedom, with 3 active modes. Consequently, it makes sense to compare the first eigenvalue of the three-residue PCA against the first one-third of the eigenvalues of the full protein, which consists of 8 modes; this comparison is summarized in Table 1. Here we see that the first mode of the three MEOW residues is generally higher than the sum of the first 8 modes of the whole system in both the full trajectory and the unfolded substate; this suggests that the conformational variability captured by the optimal low-resolution representation is comparable or even higher than the one entailed by a much more detailed description of the molecule. In the folded state, in contrast, the covariance of the first third of the whole system modes is approximately 10% higher than that of the three selected residues’ first mode. This can be ascribed to the fact that in the folded basin the molecule performs small-amplitude, rather unspecific fluctuations about the native conformation, and to properly describe them only three atoms, however carefully selected, are less informative than ten.

Lastly, we aimed to assess the statistical significance of the data obtained from analyzing the PCA modes of the top three amino acids selected by the MEOW approach by comparing them against ten groups of randomly selected amino acid triplets; the results are reported in the lower row of Figure 7 as well as in the last column of Table 1. From these data, we note that the first PCA mode eigenvalue of the MEOW triplet is always larger than the average of the first eigenvalue of the randomly selected triplets: this shows that, in comparison with the latter, the triplets identified by the MEOW approach better discriminate the conformational state of the system, or, analogously, show a larger drop between the first and the second eigenvalue,^50–52^ and are thus better suited to recapitulate the collective behavior of the protein.

In summary, we observe that the protocol was able to mark as relevant, in an unsupervised manner, those residues that are involved in the early stage of the folding process of chignolin and those that are responsible for the main native interactions in the folded basin. In the next section, the promising results about the structural, energetic, and functional role of residues pinpointed by the MEOW approach within large conformational basins are leveraged to characterize a much finer partition of the peptide’s folding transition.

### Analysis of the Foliated Folding Transition

Having examined the full trajectory and the unfolded and folded states separately, we now turn our attention to a stepwise investigation of the folding reaction of chignolin, partitioning its reactive configurational space into isocommittor hypersurfaces (IHs). This analysis aims to highlight, through the lens of the MEOW approach, the structurally and energetically key residues of the system in each stage of the transition. We thus divided the committor range [0, 1] into 20 intervals and extracted, from the original trajectory, the subset of configurations associated with each committor bin; then, we carried out the MEOW analysis independently in each of these samples. The results of the mapping entropy optimizations are reported in the heatmap in Figure 8, which displays the relevance of each residue in the ensemble of configurations sharing the same committor value. In the Supporting Information, we also report the relevance profiles of each IH individually, see Figures S3 and S4, as well as the relevance heatmaps obtained by partitioning the committor range into 10 and 30 bins, see Figure S2.

**Figure 8:**
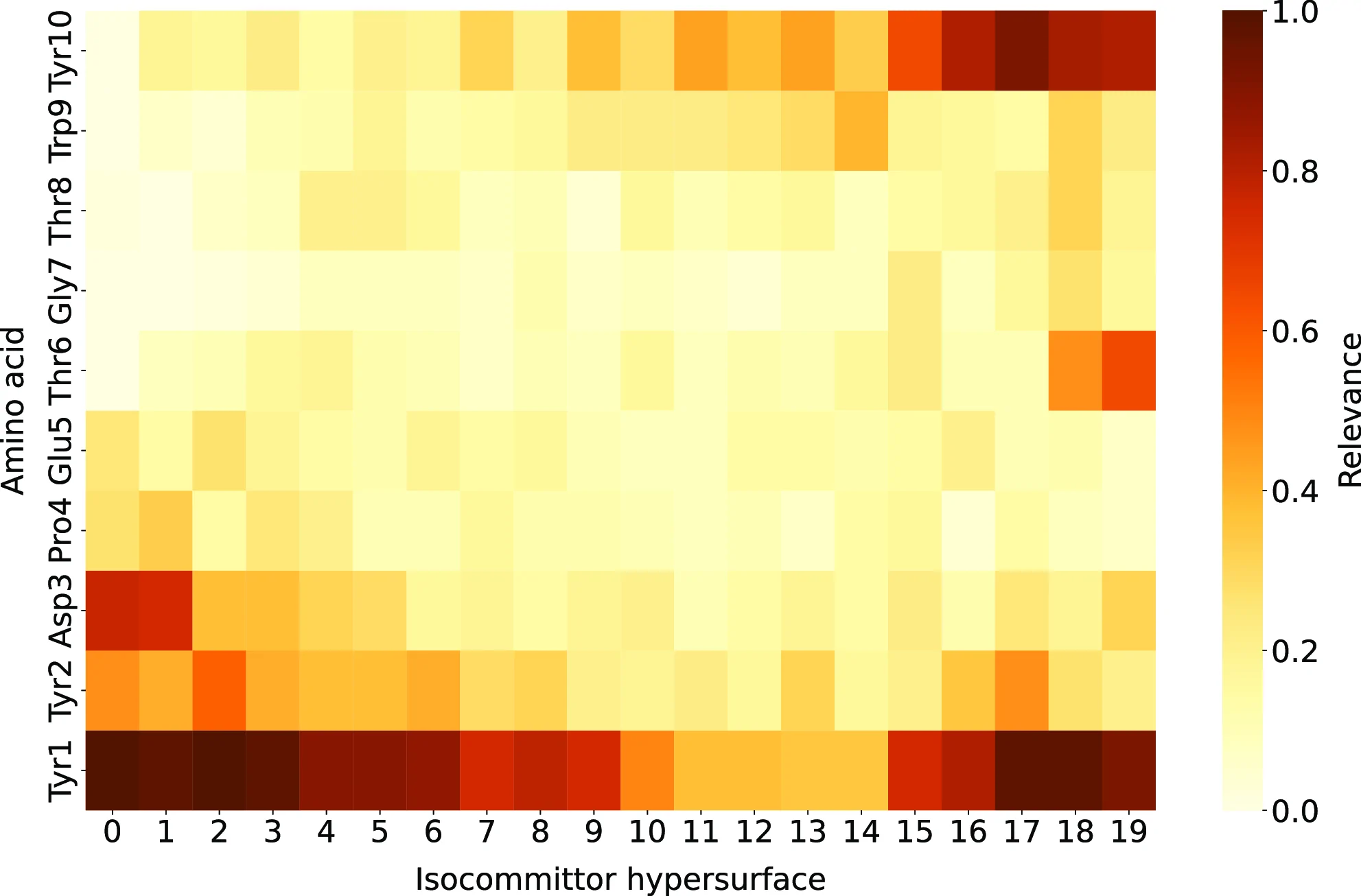
Heatmap of the MEOW relevance of each residue of chignolin throughout its folding process, partitioned into 20 isocommittor hypersurfaces. Darker colors are associated with a higher probability of the residue being retained by MEOW in the specific IH.

From the heatmap, it can be readily appreciated that each amino acid of chignolin exhibits a gradual variation in relevance as folding progresses. As it was observed in the analysis of the unfolded state (see the upper panel of Figure 6), in IH_0_, the most relevant residues are Tyr1, Tyr2, and Asp3. As the reaction progresses, Tyr1 maintains a high and rather constant relevance, whereas the values associated with Tyr2 and Asp3 decrease. In the vicinity of the transition state (*q* = 0.5,^16^ corresponding to the hypersurface IH_10_), all residues feature generally low values of relevance. In particular, in IH_12_ (see Figure 8 as well as Figure S4), the MEOW profile appears rather flat and featureless, except for the termini showing a somewhat higher signal than the rest of the peptide. This should not be interpreted as a loss of structural/energetic importance of all amino acids at once, quite the opposite: each atom and residue of the system bears as much information as the others. At this stage of the folding, the whole system cooperates to overcome the tipping point of highest free energy (Figure 4), and the MEOW approach conveys this signal in the form of a distributed relevance value; the precise nature of this behavior will be detailed further on. Any echo of the transition state is over at IH_15_, where we again observe a high relevance for Tyr1, together with that of Tyr10: here, in close proximity to the folded basin, the two terminals interact very frequently,^25,26,30^ and the system reaches a final state that is consistent with what was observed in Figure 6 (lower panel). About the folded state (IH_18_–IH_19_), we also have a high relevance for Thr6 that, as previously discussed, forms the largest amount of H-bonds within the protein (Figure S5).

The residue-wise relevance attributed by the MEOW protocol is inferred from the molecule’s conformational ensemble and energetics; it is therefore reasonable to wonder whether it reflects simpler properties, such as local flexibility, collective conformational variability, or the direct formation of native contacts. This, however, turns out not to be the case. The uniqueness of the MEOW relevance can be appreciated by contrasting it with more conventional measures, most notably distance maps, RMSF, per-residue FONC, and PCA modes. These properties were computed for each IH and expressed, when possible, in the form of a heatmap, in order to be directly comparable with Figure 8. The distance maps (Figures S6 and S7), the RMSF (Figure S8), and the per-residue FONC (Figure S9), consistently describe the evolution of the committor-conditioned conformational variability of chignolin from an extended state populating a structurally heterogeneous ensemble to a well-defined, stable conformation. The PCA explained variance and the inverse participation ratio of each principal component (Figures S10 and S11) show that the essential modes are in general rather delocalized, but do not capture coherent large-scale motions in configuration space: in fact, the covariance spectrum does not exhibit a clear gap, indicating that the dynamics is broadly distributed over several modes rather than amassed along a single dominant collective direction. It is just in IH_14_–IH_16_ that the explained variance of the first principal component is higher than 0.5, that is, when chignolin steers toward the native conformation; after IH_14_, the RMSF and the per-residue FONC heatmaps become essentially stationary. Lastly, an inspection of the per-residue PCA loadings of the first three principal components highlights the mobility of Tyr1, Pro4, and Tyr10 throughout most of the transition. The observed patterns, while informative, do not follow the evolution of the MEOW relevance throughout the folding reaction progress: overall, these comparisons thus indicate that the MEOW relevance does not simply mirror or re-elaborate properties that can be derived from more established structural descriptors; instead, by quantifying the contribution of individual residues to the construction of maximally informative low-resolution representations, it provides a complementary characterization of the configurational ensemble at different values of the committor.

As anticipated in the introduction, various works in the literature report on the possibility that the folding of chignolin can take place through two distinct routes.^26,28,31,36^ In such a case, it has to be acknowledged that the relevance profiles obtained in a given IH might actually combine the features of two different reaction pathways, which deserve to be investigated separately. To explore this possibility, in analogy with ref 31 we analyzed the system in terms of two variables first introduced and employed by Lazzeri and co-workers:^47^ the distance *d*_3–7_ between the backbone carbonyl oxygen of Asp3 and the α carbon of Gly7; and the distance *d*_2–8_ between the α carbon of Tyr2 and the backbone carbonyl oxygen of Thr8.

First, we computed the free energy landscape of the whole trajectory in terms of the two aforementioned variables. The inspection of this profile, reported in Figure 9, reveals several interesting features such as distinct basins and putative metastable states, as already highlighted by Lazzeri et al.^47^ Second, to follow the progress of the folding reaction along the committor, we also computed analogous free energy landscapes *restricted to each isocommittor hypersurface*. Analyzing this set of profiles, which are reported in full as Supporting Information (Figures S13 and S14), we could identify distinct substates, namely the first intermediate basins *p*_0_ and *p*_1_ and the second intermediate basins *p*_0_^′^ and *p*_1_^′^. These are marked in Figure 9, together with the unfolded and the folded state, to provide a mnemonic reference for the following discussion.

**Figure 9:**
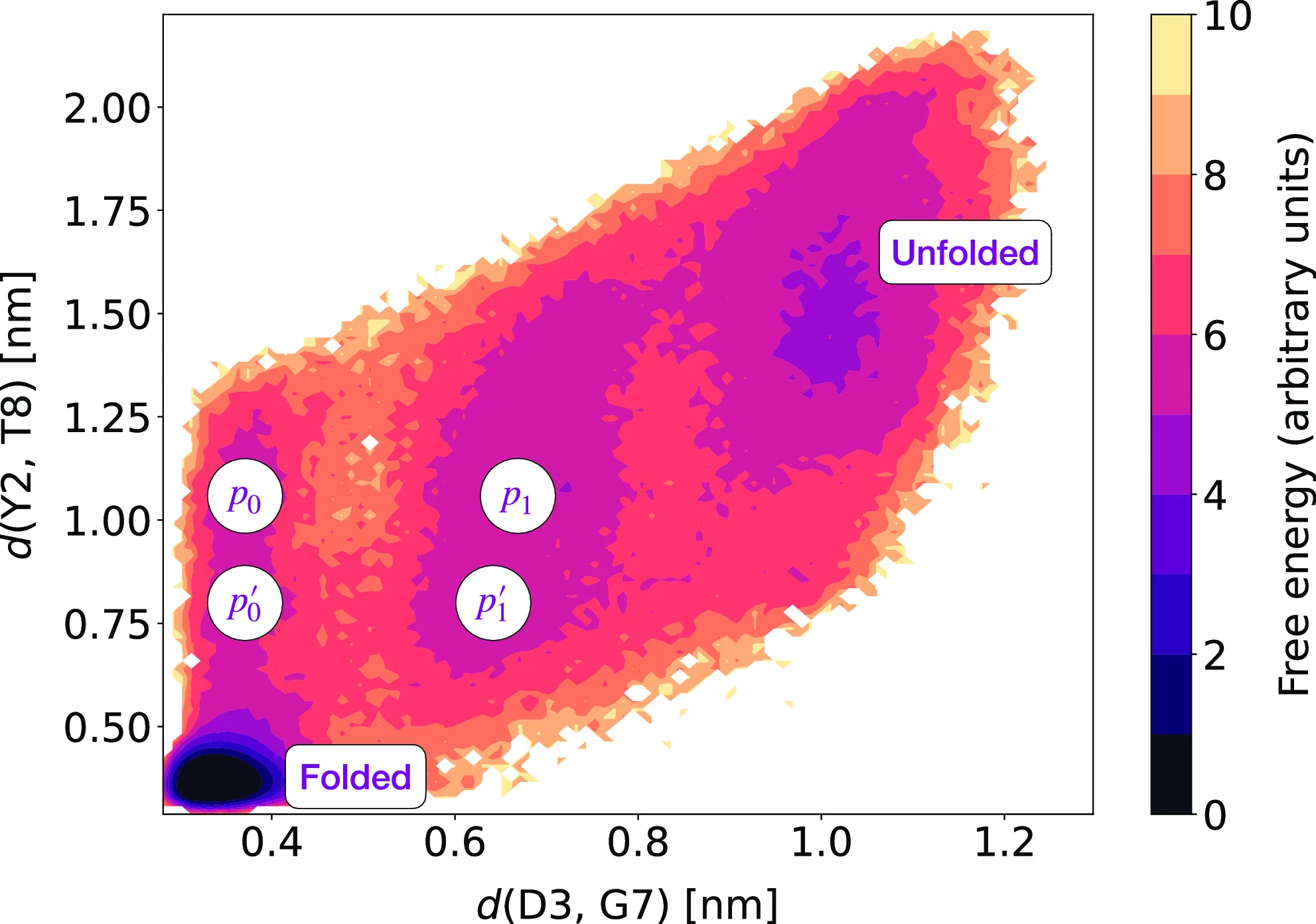
Free energy landscape of chignolin in terms of the variables *d*_2–8_ and *d*_3–7_ (see text). The labels on the plot mark the positions of the unfolded state, the folded state, and particular intermediate states discussed in detail in the main text.

Looking at IH_0_ (Figure S15), from the unfolded state the system immediately populates the state labeled as *p*_1_, and then *p*_0_. Both states have a value of *d*_2–8_ ∼ 1 *nm*, but rather different values of the distance *d*_3–7_. A quantitative inspection of the number of configurations in IH_1_ as a function of this latter distance shows that a value of *d*_3–7_ = 0.505 nm adequately serves as a threshold to discriminate the two states, with configurations with *d*_3–7_ below the threshold belonging to *p*_0_, and those above the threshold grouping in *p*_1_. The division is illustrated in Figure S16. While, by construction, in *p*_0_ the distance *d*_3–7_ is smaller than in *p*_1_ (and then closer to values attained in the folded state), the former is on average *farther* from the native structure than the latter (see distribution of MSD with respect to the native conformation in IH_1_, leftmost part of Figure S17); this suggests that *p*_0_ corresponds to an arrangement in which the two halves of the protein are close but the overall structure is distorted with respect to the native conformation, while configurations in *p*_1_ are on average relatively native-like, but the protein is more “open”.^26^ Subsequently, between IH_1_ and IH_4_, the average distance *d*_2–8_ decreases for both *p*_0_ and *p*_1_, indicating the progressive closure of the termini; in the course of this transition, we observe the emergence of the new states *p*_0_^′^ and *p*_1_^′^ highlighted in Figure 9.

Between IH_5_ and IH_9_, the state *p*_1_^′^ becomes fainter, while *p*_0_^′^ accumulates toward values of the distance variables close to those of the native conformation. At the nominal transition state (IH_10_), the overwhelming majority of the configurations have fixed the distances *d*_2–8_ and *d*_3–7_, the remainder of the transition consisting of a stabilization of the rest of the molecule. A trace of *p*_1_^′^, though, persists up to IH_16_, after which it merges with the native state: this is suggestive of a secondary folding scenario by which the molecule first acquires a conformation generally similar to the native structure but wider, then stabilizes. In contrast, the main mechanism appears to be the rapid formation of a compact core in a “bent” arrangement, then the closure of the termini, and eventually a rearrangement into the native conformation. All in all, these results are consistent with the hypothesis of a folding pathway such that the molecule, immediately out of the unfolded basin, features native-like topology and secondary structure,^27^ and progresses along one of two alternative routes: the main one that starts with the formation of the turn, the secondary one with a global collapse of the structure driven by the interactions between the termini.^26,31,47^

A further, quantitative characterization of the folding reaction along the committor was done by computing the average fraction of native contacts ⟨*Q*⟩ within each IH, alongside the associated susceptibility χ_*Q*_ defined as the variance of *Q*; the dependence of these quantities on the committor *q* is reported in the upper and lower panels of Figure 10, respectively. These plots show that ⟨*Q*⟩ grows monotonically with the committor, suggesting a substantial degree of correlation between the two quantities. Interestingly, we observe that, at the transition state, half of the native contacts are already formed.^27^ The susceptibility, on the other hand, showcases a more complex behavior: in the unfolded and folded states, χ_*Q*_ attains its lowest values, as one can expect from the fact that for *q* ≃ 0 the molecule has formed virtually no native contact, while for *q* ≃ 1 we have *Q* ≃ 1 in practically all conformations of the IH. In between, however, we observe a rapid growth of χ_*Q*_ to higher values, followed by a decrease, a local maximum after the transition state, and then a further decline.

**Figure 10:**
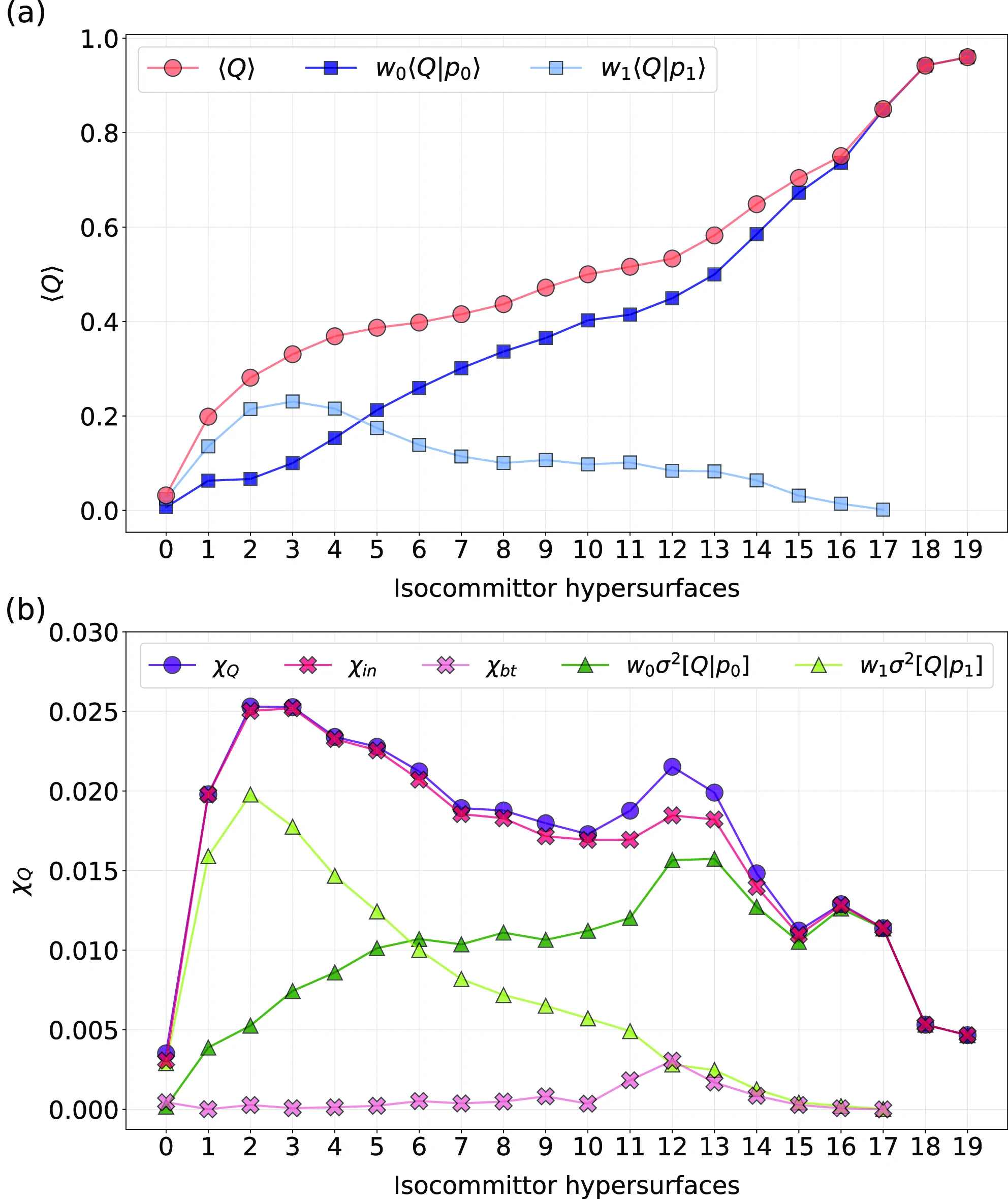
Fraction of native contacts and associated susceptibility across the isocommittor hypersurfaces of chignolin. (a) Average FONC ⟨*Q*⟩ and its contributions from the substates *p*_0_ and *p*_1_ for each IH, see eq 7 of the main text. (b) Susceptibility χ_*Q*_, its decomposition into χ_in_ and χ_bt_, and contributions to χ_in_ from the substates *p*_0_ and *p*_1_ for each IH, see eqs 7 and 8.

To make sense of this pattern, we decomposed the committor-dependent average FONC ⟨*Q*⟩ and associated susceptibility χ_*Q*_ in a way that accounts for the structural heterogeneity evinced from the free energy profiles hitherto discussed; specifically, we subdivided the configurations in each IH according to their value of *d*_3–7_, as was done for the states *p*_0_ and *p*_1_. Note that, in the following, we employ these labels for the groups of configurations respectively below and above the *d*_3–7_ ≈ 0.505 nm threshold used for defining *p*_0_ and *p*_1_, even for those IHs in which we know that these states do not provide a valid description of the conformational diversity of the system. In fact, the last two IHs do not contain any configuration with *d*_3–7_ above the threshold. This notwithstanding, the following analysis provides interesting insight into the behavior of the system, as detailed hereafter.

Having partitioned all configurations in each IH according to their belonging to one of the two states, we then computed ⟨*Q*⟩ and the total susceptibility χ_*Q*_ as the sum of two terms, namely

7 $$\begin{array}{l} \left\langle Q\right\rangle =\sum_{i=0,1}w_{i}\left\langle Q|p_{i}\right\rangle \\ \chi_{Q}=\chi_{in}+\chi_{bt} \end{array}$$

where

8 $$\begin{array}{l} \chi_{in}\equiv \sum_{i=0,1}w_{i}\sigma^{2}\left[Q|p_{i}\right]\\ \chi_{bt}\equiv \sum_{i=0,1}w_{i}{\left(\left\langle Q|p_{i}\right\rangle -\left\langle Q\right\rangle \right)}^{2} \end{array}$$

In eqs 7 and 8, within each isocommittor hypersurface and for each state *p*_*i*_, *w*_*i*_ is the fraction of configurations belonging to the state, and ⟨*Q*|*p*_*i*_⟩ and σ^2^[*Q*|*p*_*i*_] are the state-restricted mean and variance of *Q*, respectively. The terms χ_in_ and χ_bt_ measure the contributions to the susceptibility coming, respectively, from the heterogeneity *within* and *between* the putative pathways. The results of this decomposition are reported in Figure 10.

First, we observe that the main contribution to the FONC in the early stages of the folding comes from *p*_1_, see Figure 10a, but at about IH_5_ it starts to deplete and rapidly gives way to the state *p*_0_. Additionally, as soon as the system leaves IH_0_, *p*_1_ shows higher values of ⟨*Q*⟩ than *p*_0_ (see Figure S18, upper panel); this observation corroborates the interpretation of the former substate as more native-like than the latter, as we proposed based on its smaller MSD from the native state (Figure S17, upper left panel).

Second, we note that the variance *between* the states, χ_bt_, contributes to the total χ_*Q*_ by an almost negligible amount, see Figure 10b. The total susceptibility, in fact, is for the most part a weighted average of the variance of *Q* computed in the two putative pathways separately; these, however, do not contribute equally: the term associated with the *p*_1_ state peaks right after the unfolded state, consistently with the conformational breadth of this basin and the formation/disruption of early native contacts that, as noted above, takes place mainly in *p*_1_. Its contribution, however, rapidly decreases as the state gets less and less populated.

The *p*_0_ term, in contrast, grows monotonically and peaks at IH_12_–IH_13_, then decreases after a small bump, see Figure 10b. The main peak takes place right after the nominal transition state (*q* = 0.5) and in proximity of the committor free energy maximum (Figure 4), and corresponds to a stage in which the conformational variability as well as the fraction of native contacts experience important fluctuations. This maximum in χ_*Q*_ is thus a true hallmark of cooperativity, and it explains the “flat” relevance profile observed in IH_12_ as a consequence of the large-scale, collective fluctuations in the molecule. The second, local maximum of χ_*Q*_ at IH_16_, instead, coincides with the conclusive step that, as mentioned earlier, takes place at this stage with the full stabilization of the native contacts and the *d*_3–7_, *d*_2–8_ variables attaining their final values.

Based on these observations, we have carried out further detailed analyses on the two most notable IHs, specifically (i) IH_1_, immediately after the system has abandoned the unfolded basin; and (ii) IH_12_, which corresponds to the peak of the susceptibility χ_*Q*_ right after the transition state, see Figure 10b. The configurations of IH_1_ have been separated in the states *p*_0_ and *p*_1_ based on their value of *d*_3–7_. In IH_12_, instead, *d*_3–7_ is not anymore a good descriptor to distinguish putative conformational substates, as it is made evident by the low values of weighted fraction of native contacts and susceptibility of *p*_1_ with respect to *p*_0_ (Figure 10a and b); the peak in χ_*Q*_, however, suggests that the system still endows a large structural variability, which is thus worth characterizing in some detail. Hence, in IH_12_ we have resorted to an MSD-based *K*-medoid clustering to identify two groups of structurally distinct configurations (further details on the clustering procedure are provided as Supporting Information). For both pairs of configuration clusters in which IH_1_ and IH_12_ were partitioned, we carried out a mapping entropy optimization analysis to identify those residues that show particular relevance in each substate, the associated results being reported in Figure 11. Additionally, we inspected structural and energetic properties of these pools of configurations, namely the contact maps, the RMSF profiles, and the frequency of ring-stacking interactions (see Figures S19 and S20).

**Figure 11:**
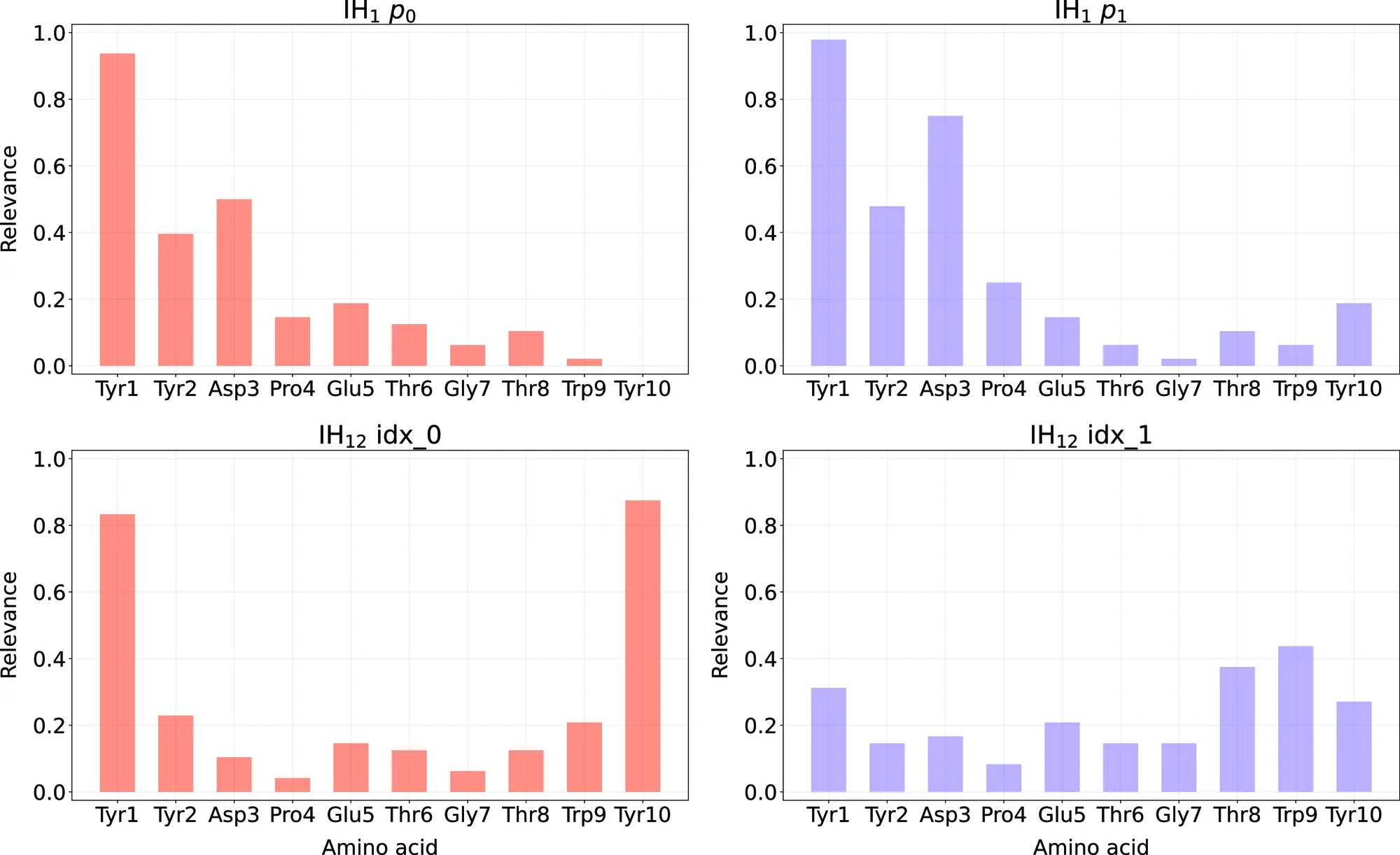
Relevance profiles resulting from the application of MEOW to the two clusters (*p*_0_ and idx_0 in orange on the left, and *p*_1_ and idx_1 in blue, on the right) of IH_1_ (top panel) and IH_12_ (bottom panel).

In IH_1_, the substates *p*_0_ and *p*_1_ display similar relevance patterns (top row of Figures 11 and S19), both of which mirror the profile of the completely unfolded conformation of the peptide depicted in Figure 6. The remarkable asymmetry of the relevance patterns in Figure 11 with respect to the peptide sequence, already visible in the analysis performed in the unfolded basin, can be at least in part explained by a relatively small degree of conformational variability in residues 5–10, all of them having RMSF about 6 Å (see S19). In contrast, the first four amino acids of the protein cover a broader range of RMSF (5–8 Å), which hints at a more complex pattern of interactions and conformational arrangements. In particular, in *p*_0_ as well as in *p*_1_, the residue with the highest MEOW relevance is Tyr1, followed by Asp3 and Tyr2. In both clusters, the two tyrosines showcase a high mobility with an RMSF ∼ 8 Å, while Asp3 has values in the range 5–6 Å. A frequent stacking interaction between Tyr1 and Tyr2 (see Figure S19) and the contacts between Tyr1 and Asp3,^25,30^ as anticipated in the discussion of the unfolded state, explain the high relevance of these three residues. Tyr10 appears to gain some relevance in *p*_1_, possibly as a consequence of a slightly higher flexibility (RMSF ∼ 7 Å). Interestingly, the similarity of the relevance patterns of the clusters mismatches their structural differences, which appears from the inspection of their contact maps presented in Figure S19: in *p*_0_ we observe a tripartite arrangement, with rather defined blocks encompassing residues 1–3, 4–6, and 7–10; in *p*_1_, on the other hand, we have an overall more compact organization of the peptide, with the residues within each half of the chain getting closer among them. The MEOW analysis of the two substates identified in IH_1_, which are putatively ascribed to distinct folding pathways, in combination with a detailed inspection of the structural features of their ensembles, reveals remarkable conformational differences that go in tandem with a strikingly similar relevance pattern, which, as previously highlighted, is furthermore quite consistent with that of the unfolded state. These results are suggestive of a folding mechanism that, at the very early stages of the process, can take structurally different routes; these, however, seem to rely on the same key elements, most notably the interactions involving Tyr1, Tyr2, Asp3 and, to a lesser degree, Tyr10.

The MSD-based K-medoid partition of IH_12_ returns two markedly unbalanced clusters (see Table 8 in SI): the more populated one, idx_0, features a high relevance on the termini and a rather uniform profile through the rest of the sequence (top row of Figure 11 and Figure S20); in contrast, for the less populated cluster idx_1 the relevance profile is rather flat (bottom row of Figure 11 and Figure S20). These results reflect the characteristics of the associated contact maps, reported in Figure S20, which highlight the presence of distinct compact cores in the two clusters: in idx_0, the protein appears to have a quite large and packed bulk, accompanied by more open and flexible termini whose high relevance observed in this cluster is thus likely due to their large distance and mobility. On the other hand, cluster idx_1 shows a smaller, compact center of the peptide, and a dense group of residues close to the C-terminal. The partition of IH_12_ into two structurally dissimilar clusters does not allow one to clearly identify distinct conformational basins: both share similar features and, with the exception of the termini, similar uniform relevance profiles; furthermore, we note that both relevance patterns are consistent with that obtained in the analysis of the whole isocommittor hypersurface (Figure S4). This result is not suggestive of a well-defined conformational arrangement of the molecule, but rather of a large degree of variability in structure and interactions, already hinted at by the peak in the FONC susceptibility χ_*Q*_ in this IH. In conclusion, the analysis carried out in this IH describes a conformational ensemble that, in spite of the large (>60%) average fraction of native contacts, features an important structural variability near the transition state, and supports the hypothesis of a close-to-native post-transition conformation (see upper right panel of Figure S17) later stabilized by the fixation of interstrand contacts.^26,31,34,36^

### *A Posteriori* Validation through AlphaFold-Based Mutations

The TPT+MEOW pipeline applied to the folding reaction of chignolin has allowed us to identify which residues, in each structural ensemble characterized by a given value of the committor, have a particular significance from the standpoint of conformational informativeness; that is, which residues retain the greatest amount of information about the structural and energetic variability of the ensemble. Different independent measures have been employed to verify the coherence of the resulting signals with more standard MD simulation analyses, as well as with the body of knowledge gathered about chignolin folding through simulation and experiment; the picture that emerges is that the residues bearing higher relevance seem indeed to entail a functional role. As a last step, we tested this hypothesis through a simple, yet significant predictive strategy, namely, an *in silico* mutation analysis.

More specifically, we considered four representative IHs (IH_1_, IH_6_, IH_12_, and IH_19_): for each of them, we produced two mutant sequences, replacing the two residues with the highest MEOW relevance and, separately, the two residues with the lowest MEOW relevance with alanines; the resulting sequences are reported in Table 2. The native ensemble corresponding to each mutant was then obtained through the AlphaFold3 (AF) structure prediction server:^14^ for each mutation, 10 putative structures were generated independently using AF; furthermore, in order to have a common reference, 10 structures obtained from the original CLN025 sequence were generated as well. For each predicted structure, the model with the highest confidence score was used to perform a molecular dynamics simulation with the same force field and settings employed in the work by Lazzeri et al.;^31^ the structures were equilibrated using a V-rescale thermostat for 50 ns, and then a C-rescale barostat for 100 ns. The last frame of the equilibration phase of each run was aligned to the crystal structure of CLN025 (PDB: 2RVD), and the backbone RMSD with respect to the crystal was computed; lastly, we averaged the RMSD among the 10 MD-equilibrated structures of each sequence.

**Table 2:** Sequences Used in the Mutational Study. For IH_6_ Low 2, Pro4 and Thr6 had the same value of relevance, so they were both mutated, along with Glu5. IH_12_ and IH_19_ share the same Top 2 mutated structure.

type	Top 2	Low 2
CLN025	YYDPETGTWY	
IH_1_	**A**Y**A**PETGTWY	YYDPET**AA**WY
IH_6_	**AA**DPETGTWY	YYD**A**E**AA**TWY
IH_12_	**A**YDPETGTW**A**	YYD**A**ET**A**TWY
IH_19_	**A**YDPETGTW**A**	YYD**AA**TGTWY

The results of the mutation analysis are summarized in Figure 12 and Figure S21, while more detailed data is reported in Table S9 of the SI; note, in passing, that the top-ranking mutated amino acids of IH_12_ are the same as IH_13_. When the mutation affects the two residues with the lowest relevance, the RMSD remains close to that of the WT sequence of CLN025 (1.21–1.23 Å) in all cases under examination. Conversely, altering the two residues with the highest relevance leads to a more diversified behavior: for the top-ranking residues in IH_1_ the deviation is comparable to that observed mutating the amino acids with lowest relevance; in contrast, the mutation of the top-ranking residues of IH_6_, IH_12_, and IH_19_ (the last two having the same top residues) induces an appreciable structural distortion, which results in a larger increase in RMSD compared to the WT.

**Figure 12:**
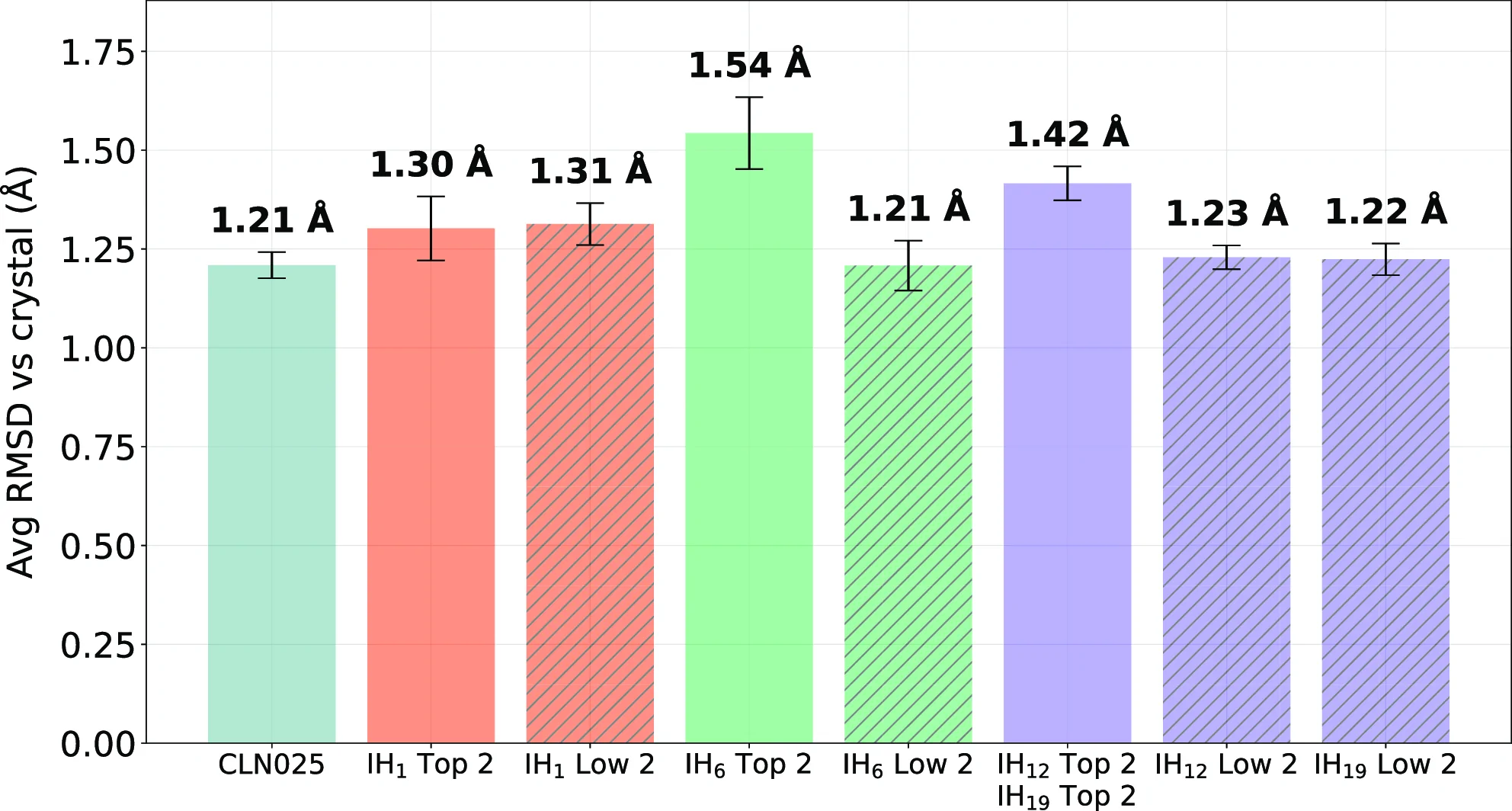
Mean values of RMSD of the backbone with the 95% confidence intervals for wild-type CLN025 and the mutated chignolins against the standard crystal structure for CLN025 (PDB: 2RVD). The hatched bars represent the structures where the mutated amino acids had the lowest values of relevance.

The picture that emerges is that the mutation of the two residues with the lowest relevance does not alter the predicted native structure substantially, while a mutation of the two residues with the highest value of relevance does modify the predicted fold in a statistically significant manner. Interestingly, though, this change does not occur in the case of IH_1_ but only for the top-ranking residues identified in the following stages of the reaction pathway. This observation is consistent with the hypothesis, previously discussed and supported by our analyses as well as by independent evidence,^27,28,31^ that chignolin attains a native-like conformation rather early in the course of the reaction, but the native state is correctly stabilized only in a later stage. The combined TPT/MEOW approach thus identifies residues that are informative about each stage, and the *in silico* structure prediction analysis highlights that certain residues from different stages bear different importance for the precise conformation of the native state.

This AlphaFold-based mutation analysis is certainly limited in scope, for at least two reasons: first, the prediction is based on the amino acid sequence alone and does not account by any means of the folding process, thereby preventing us from observing how changing certain residues affects the reaction pathways; a much more accurate and informative investigation would require the extensive simulation each protein mutant from an unfolded conformation to its native state. Second, because of the small size of the protein under examination, the impact of noise might be large: we acknowledge that the comparison between the mutations of low-ranking and top-ranking residues gave neatly separated RMSD values with sufficiently small statistical dispersion, but at the same time we expect this type of probe to have much more discriminating power in a larger protein, where a solid body of control mutations on irrelevant residues can be gathered. On the other hand, this kind of analysis has returned results coherent with those obtained so far and represents a potentially effective protocol for further investigations along the lines of those presented here.

## Conclusions

The CLN025 variant of the 10 amino acid long miniprotein chignolin undergoes a fast two-state folding transition with a very stable native conformation.^25^ Despite its small size and relatively simple structure, chignolin features a nontrivial folding mechanism. In fact, various works in the literature describe chignolin as a cooperative two-state folder, but at the same time support a two-route folding scenario in which distinct, alternative pathways compete;^26,31,33^ furthermore, *in silico* studies have also shown a nontrivial dependence on the fine detail of the folding mechanics on the simulation/analysis approach^30,31,33,36^ or the force field employed.^28,34^ In general, then, a neat, mechanistic understanding of the folding pathway of chignolin is still lacking, and the rationalization of its features can lead to a better understanding of this as well as larger and more complex systems.

In this work, we employed a novel computational pipeline to perform an unsupervised analysis of unbiased MD trajectories of chignolin to identify those amino acids that retain the largest amount of information about the conformational and energetic variability in each stage of its folding transition. To this end, two ingredients are required: first, a quantitative parameter to describe the progress of the reaction; second, an unsupervised, general definition of the functional relevance of an amino acid in a given conformational ensemble. These needs are aptly answered by the combination, presented here for the first time, of transition path theory and a mapping entropy optimization workflow.

Given a plain, unbiased molecular dynamics trajectory that explores in a statistically significant manner the folded and unfolded states of a protein, as well as the reactive trajectories joining them, TPT allows one to label each configuration based on the value of its committor *q*(**x**), which is the probability that a reactive trajectory starting from a configuration **x** will reach the folded state before going back to the unfolded basin. In this work, we combined four independent, unbiased MD simulations of chignolin^31^ and grouped the associated frames according to their committor values, in order to have a statistically relevant sample of the configuration space at different stages of the folding reaction.

The second task, namely the identification of mechanically/energetically relevant residues, is carried out through the mapping entropy optimization workflow.^1,20–23,45^ This approach quantifies the information about the conformational variability of a protein that is lost when, instead of describing it in terms of all its atoms, only a subset of them is employed. Critically, such selection can be performed so as to minimize the loss of information: this allows one to pinpoint those atoms and residues that entail key structural and energetic features of the system. In various works carried out by some of us, the key residues identified by MEOW have been associated with functional regions of the proteins under examination.^20–22,45^ This correlation between the relevance attributed by MEOW to a residue and its role in the protein’s behavior was also observed in the case of chignolin.

The analysis we carried out pinpointed Tyr1, Tyr2, Asp3, and Tyr10 as the residues with the highest relevance when the whole trajectory is considered; in contrast, individually focusing on the initial and final basins of the transition, Tyr1 and Asp3 are specifically associated with the denatured, unfolded state, while Tyr1, Asp3, Thr6, and Tyr10, which are key in the stabilization of the native structure,^25,30,33^ are the signature of the folded state. A simple but insightful analysis of the conformational covariance explained by the top three residues identified by MEOW further corroborated the high degree of informativeness that they entail. In particular, we observed that the first PCA mode of these specific amino acids is associated with an eigenvalue that is appreciably higher than that obtained by choosing three residues at random: the selection operated by MEOW is thus optimal for the definition of a low-resolution description of the system that returns the largest amount of information about its conformational variability.

Subsequently, we foliated the transition region, subdividing the *q* ∈ [0, 1] range in 20 bins, and applied the MEOW protocol in each of them separately. Our analysis returned results consistent with what we observed for the unfolded and folded basins, as well as a gradual evolution from the former to the latter. In particular, we addressed the hypothesis, suggested by various works in the literature, that chignolin folds via two main pathways; a detailed analysis of the free energy profiles as a function of two structural variables–computed for the whole trajectory as well as in each IH separately–indeed highlights, already at the earliest stages of the folding process, the appearance of two distinct channels, characterized by different values of the distance between residues Asp3 and Gly7.

A systematic analysis of the fraction of native contacts and, in particular, of its variance as functions of the committor allowed us to distinguish key features of the folding process along the two pathways. The state *p*_1_, corresponding to high values of *d*_3–7_, showed a peak in the weighted variance of *Q* in the earliest phases of the folding; in contrast, the state *p*_0_, associated with lower values of *d*_3–7_, peaked immediately after the nominal transition state at *q* = 0.5, indicating large-amplitude conformational fluctuations and a cooperative, collective behavior. This feature was captured by the MEOW protocol, which in IH_12_ returned a rather uniform pattern of relevance values across the whole chain, pointing to a distributed importance of all residues at this critical transition point.

These properties of IH_1_ and IH_12_ suggested a conformational heterogeneity worthwhile to characterize in detail. We thus grouped the configurations belonging to distinct free energy basins in these hypersurfaces: in the first case we distinguished the two states on the basis of the *d*_3–7_ distance, while in the second case we performed a structure-based K-medoid clustering with *K* = 2; subsequently, we carried out a MEOW analysis of the clustered configurations. The results of the analysis are qualitatively different: in IH_1_ the relevance patterns are rather similar despite a marked separation of the conformations in two distinct clusters; in contrast, in IH_12_ the relevance profiles are rather uniform, and the structural differences between the conformational clusters are minor. A detailed structural analysis of the clusters provides a mechanistic, in-depth background for the observed relevance patterns, highlighting the complex interplay of factors behind the simple yet significant information distilled by the MEOW approach. Taken together, these results support the hypothesis that the amino acids that participate more prominently in the various phases of the folding process do not bear a simple correlation with the overall structural arrangement of the molecule, but can nonetheless be identified and, possibly, leveraged as probes for the study of the protein.

This hypothesis was tested through a mutation analysis aimed at measuring to what extent the mutation of residues deemed relevant by the MEOW approach led to a disruption of the predicted native fold. We mutated to alanines the two residues with the highest relevance in selected IHs and employed the AlphaFold structure prediction tool to generate an ensemble of corresponding native conformations. Pairs of amino acids relevant in the midlate stage of the folding transition determined predicted structures on average more distant from the reference wild-type structure than those obtained by mutating the two least relevant residues; furthermore, this effect was not observed for residues relevant in the early stage of the folding, supporting the hypothesis that the chignolin native state is critically determined by residues that play an important functional role deep into the reaction progress.

The effectiveness of the proposed approach clearly relies on specific prerequisites, most importantly the reliability of the force field employed to perform the underlying simulations, the statistical coverage of the sampled trajectories, and the accuracy of the parametrization of the committor model; limitations in these characteristics of the input naturally reflect on the output, as it is the case for any analysis method, and they have to be factored in when interpreting the results. On the other hand, the generality of the method represents a major strength in that it can be applied to a variety of specific cases with no or minimal adaptation: this flexibility is a consequence of the robustness and simplicity of the concepts underlying the algorithms that constitute the pipeline.

In conclusion, the combination of TPT and MEOW in the pipeline presented in this work has been shown to be effective in retrieving amino acids of miniprotein chignolin that have critical importance along its folding pathway. The MEOW framework is unsupervised, system-agnostic, and the information it returns is inherently multibody (that is, it cannot be obtained from simple, elementary sets of local interactions between atoms^20,22^ ); its integration with TPT paves the way to a deeper understanding of protein function not only in the native basin^20^ and/or in different conformers,^22^ but also in the course of folding and other relevant reactive processes, e.g., ligand binding, conformational interconversion, allosteric transitions, etc. The identification of those residues that play a key structural, mechanical, and energetic role in such processes may prove instrumental also to actively intervene in the system’s function, e.g., to design proteins or ligands with tailored characteristics, or to identify targets for pharmaceutical drugs to enhance or suppress a protein’s activity even before it reaches the native state.^53^

## Supplementary Material



## Data Availability

The raw data associated with this work are freely available on the Zenodo repository at https://zenodo.org/records/19094879, while the CLN025 trajectory data and the neural network used to calculate the committor can be found at https://zenodo.org/records/8172628. The molecular dynamics simulations of the AlphaFold-generated structures can be obtained upon reasonable request.

## Notes

Chignolin data postprocessing and analysis; EXCOGITO parameters used to perform MEOW; Technical details on the calculation of contact maps, fraction of native contacts, *K*-medoid clustering; Full MEOW results for the transition region for 10, 20, and 30 IHs; Free energy projections on the 20 IHs and on a finer division of IH_0_; MSD distributions in the 20 IH; Distances distributions for IH_1_ and IH_12_ and their detailed analysis; Quantitative analysis of the fraction of native contacts, and the fraction of H-bonds per amino acid per IH; Independent measures on the 20 IHs (RMSF of C_α_ atoms, distance maps, fraction of native contacts pe residue of the C_α_ atoms, PCA explained variance, inverse participation ratio, PCA loadings); Graphic representations of the AlphaFold-generated structures against the original CLN025 (PDF)
