# Supplementary material for: Detailed Insight into the Chignolin Folding Process from Maximally Informative Low-Resolution Representations of Its Isocommittor Hypersurfaces
Source: J Chem Theory Comput. 2026 Aug 26;22(18):9831–46. doi: 10.1021/acs.jctc.6c01478 (PMC13602922; doi:10.1021/acs.jctc.6c01478)
Supplement: Supplementary file 1 [file ct6c01478_si_001.pdf]

Supplementary material to
Detailed insight in the chignolin folding process from maximally informative low-resolution representations of its isocommittor hypersurfaces

Alessia Guadagnin Pattaro,[1,2] Roberto Menichetti,[1,2,*] and Raffaello Potestio[1,2,†]

[1] *Physics Department, University of Trento, via Sommarive, 14 I-38123 Trento, Italy*
[2] *INFN-TIFPA, Trento Institute for Fundamental Physics and Applications, I-38123 Trento, Italy*

(Dated: August 20, 2026)

* roberto.menichetti@unitn.it
† raffaello.potestio@unitn.it

CONTENTS

S1. Data analysis and AI usage S3

S2. Chignolin data S3

S3. EXCOGITO and subroutine `excogito optimize` S3
A. Full trajectory optimizations S4
B. Isocommittor hypersurfaces optimizations S4

S4. Distance Maps S5

S5. Fraction of Native Contacts S5

S6. K-Medoids clustering S6

S7. Mapping entropy optimizations of clustered IH_1 and IH_{12} S6

S8. SI: Results S7
A. Measures across the isocommittor ensembles S7

References S9

S1. DATA ANALYSIS AND AI USAGE

All analyses of the project have been done with Jupyter Notebook running Python-3.11, in particular using the libraries MDAnalysis [1, 2] and MDTraj [3]. Data analysis was operated on a personal MacBook Pro M4 with macOS 15. The code used for data analysis was refactored with Google Gemini and ChatGPT to reduce the execution time and to improve readability.

S2. CHIGNOLIN DATA

Unbiased molecular dynamics (MD) trajectories of the chignolin structure (variant CLN025, amino acid sequence YYDPETGTWY) were provided by Gianmarco Lazzeri and Roberto Covino at the Frankfurt Institute of Advanced Studies (FIAS) [4]. The duration of the unbiased trajectories is presented in Table S1.

Runs 1, 2, and 3 are generated from run 0 at time $13\,\mu s$ such that they perform a greater exploration of configurational space to have a proper sampling. According to the authors, the trajectories contain up to 50 folded/unfolded transitions.

Table S1: Simulation times of the unbiased trajectories of chignolin employed in the project.

**Unbiased trajectory**	**Time length** (μs)
`run0.xtc`	~ 40
`run1.xtc`	~ 27
`run2.xtc`	~ 27
`run3.xtc`	~ 27

The unbiased MD simulations were performed by using the Goethe-HLR cluster of Goethe University Frankfurt (general1 Intel Skylake GPU) with GROMACS 2022.5. The force field CHARMM22 was used to replicate the same simulation set-up employed by Lindorff-Larsen *et al.* [5]. The production run was operated using the V-rescale thermostat [6] at a temperature of $340\,K$, close to the experimental chignolin denaturation temperature [7], and the Parrinello-Rahman barostat [8] at a pressure of $1.0\,atm$. Positions and velocities were written every $50\,ps$ with an integration step occurring every $2\,fs$.

The four trajectories were joined using GROMACS 2021.4 yielding a total time of ~ $120\,\mu s$ (119331192 frames). Then, to perform the analysis in a more homogeneous way, the trajectory was divided into four smaller trajectories of ~ $30\,\mu s$ (298327 frames) each.

S3. EXCOGITO AND SUBROUTINE `EXCOGITO OPTIMIZE`

EXCOGITO [9–12] is a software suite used to analyze all-atom MD simulations using coarse-grained representations of the system and mapping entropy. The software is freely available on Github.

The tool retrieves the most informative representations of a system after the latter undergoes a coarse-graining procedure, meaning that it is able to find the mapping operators $\mathbf{M}^N(\mathbf{r}^n)$ selecting the subset of atoms in the system that minimize the mapping entropy $\tilde{S}_{map}$. The selected subset of atoms is hence maximally informative about the statistical behavior of the protein in spite of the reduction of the number of degrees of freedom. This operation is done via the subroutine `excogito optimize`, which performs a Monte Carlo (MC) optimization of the coarse-grained (CG) representation of the system via a $\tilde{S}_{map}$-based simulated annealing procedure. The coarse-grained structures are obtained via agglomerative bottom-up hierarchical clustering with average linkage (UPGMA).

The mapping entropy optimizations were launched on the HPC cluster owned by the University of Trento, performing a total number of parallel optimizations equal to 48, this is necessary to assure a proper sampling of the rugged mapping entropy landscape, since no local minima is preferred over the others. The statistical analysis performed over the 48 optimizations allows one to retrieve the atoms that are in the pool of optimized solutions that might have a biological role within the molecule.

A. Full trajectory optimizations

The file preparation of the mapping entropy optimizations for the full chignolin trajectory divided into four parts were performed on a local Linux machine. EXCOGITO has an upper limit for the trajectory frame number employable in the analysis, the latter being $10,000$ when running on an HPC cluster. Since the number of frames of each original trajectory was too large, a subsampling was employed to reduce the number of frames of each trajectory to $10,000$.

The parameters used in the optimizations are reported in Table S2.

Table S2: Parameters used in EXCOGITO for the mapping entropy optimization of the full trajectories.

**atomnum**	**frames**	**cgnum**	**MC steps**	**rotmats period**	**Ncores**	**criterion**	**min nclust**	**max nclust**
93	10,000	10	2000	400	48	2	20	100

The `cgnum` parameter refers to the number of CG sites employed to describe the molecule at a lower resolution, set here as equal to the number of amino acids, hence the EXCOGITO output is the list of the 10 most retained atoms in the protein. To assure an appropriate exploration of mapping entropy space, 48 mapping entropy optimizations were run each time. The probability of each atom to be retained was then calculated by considering the occurrence of each atom of being in a mapping, divided by the total number of mappings; the relevance of each *amino acid* was considered to be the one of the atom of that residue with the maximum value of probability. The `criterion` parameter is used to choose the hierarchical clustering method, in this case the *multiple maxclust*, as reported by Giulini *et al.* [10], was used. These settings will be used throughout the whole project, unless stated otherwise.

B. Isocommittor hypersurfaces optimizations

To accurately characterize each transition step, the committor function was employed. The original committor probability distribution calculated by Lazzeri *et al.* [4] was that of the unfolding process, herein the folding process is considered and the committor will always refer to that from now on.

The distribution of different values of the committor probability was explored by dividing data into 20 partitions, the *isocommittor hypersurfaces*, that are those sets of points where $q^+(\mathbf{x}) = \mathbf{q}^+$ with $\mathbf{q}^+ \in [0,1]$ and represent a foliation of $q(\mathbf{x})$ in the transition region. In this context, IH are defined as the center of the bin and with two extremes in Equation S1.

$$\mathrm{IH}_x = \left(\frac{x}{20} + 0.05\right) \pm 0.05,\ \ x \in [0,20] \tag{S1}$$

The full chignolin trajectory was sliced according to the committor value into 20 smaller trajectories, respectively. Those trajectories that had a number of frames $> 10,000$ were pruned using a decimation criterion, while the others have been left unchanged. Mapping entropy optimizations were run on the HPC cluster using the set of parameters reported in Table S3, with number of frames, maximum, and minimum cluster number varying according to the trajectory being optimized. These parameters were computed similarly also for the case of 10 IH and 30 IH (data not shown).

Table S3: Set of parameter used for the mapping entropy optimizations of the IH. The asterisk refers to different frame numbers in the trajectories, along with the cluster number.

**atomnum**	**frames**	**cgnum**	**MC steps**	**rotmats period**	**Ncores**	**criterion**	**min nclust**	**max nclust**
93	*	10	2000	400	48	2	*	*

Appropriate values of `min_nclust` and `max_nclust` were calculated for the specific frame number, the resulting values being show in Table S4.

The error for the residue-wise relevance plots (Figures S3 and S4) was computed by inheriting the atom-wise error. The atom-wise error was calculated by considering that, for each of the 48 optimizations, each atom can either be chosen or not chosen to be part of a mapping, hence we have a Bernoulli process. The 95% confidence

interval can then be calculated as:

$$\text{rel\_err} = 1.96 \cdot \sqrt{\frac{p(1-p)}{48}} \tag{S2}$$

where $p \in [0,1]$ is the probability for an atom to be selected in a mapping.

Table S4: Number of frames and corresponding number of clusters used for the 20 bins division of IH optimizations.

**n_frames**	10,000	10,000	6833	4762	3627	3065	2568	2155	1583	1239	948
**min_nclust**	20	20	14	10	7	6	5	4	3	2	2
**max_nclust**	100	100	68	48	36	31	26	22	16	12	10

**n_frames**	796	673	688	1041	2187	4337	10,000	10,000	10,000
**min_nclust**	2	1	1	2	4	9	20	20	20
**max_nclust**	8	7	7	10	22	43	100	100	100

S4. DISTANCE MAPS

We employed distance maps to gain a more detailed picture of the structural arrangement of the molecule in each configuration. The distance maps are obtained by calculating the average positions of the center of mass (CoM) of all amino acids and measuring the pairwise distances between the average CoM. These are related to the steric hindrance of the functional groups of the neighboring residues: larger amino acids such as Tyr or Asp will have their CoM farther from the protein backbone, as opposed to smaller residues such as Gly or Pro. Hence, a close distance in the DM does not necessarily imply the occurrence of an interaction between two residues. The DM for the present paper have been built using the MDAnalysis method `distances.distance_array`.

S5. FRACTION OF NATIVE CONTACTS

The discrimination between folded and unfolded conformations of a protein occurring in the course of a MD simulation is preliminary to the definition of the reactive ensemble. A possible strategy to do so employs the fraction of native contacts (FONC) of heavy atoms, relying on the assumption that only native contacts play a dominant role in determining the folding mechanism of a protein.

The FONC is defined according to Best *et al.* [13] in Eq. S3. It considers a list of P native contact pairs (i,j), and all pairs of heavy atoms i and j belonging to residues θ_i and θ_j are considered in contact if the sequential distance between the residues $|\theta_i - \theta_j| > 3$ Å and if the distance between the heavy atoms $r_{ij}(\mathbf{x}) < 4.5$ Å:

$$Q_{heavy}(\mathbf{x}) = \frac{1}{P} \sum_{(i,j)} \frac{1}{1 + \exp\left[\beta(r_{ij}(\mathbf{x}) - \lambda r_{ij}^0)\right]}. \tag{S3}$$

The term r_{ij}^0 is the distance between atoms i and j in the native state, β is a smoothing parameter with value $\beta = 5$ Å^{-1}, and λ is a factor accounting for fluctuations in the contact. For all-atom simulations such as those involved in this paper, $\lambda = 1.8$ [13].

Folded (F) and unfolded (U) conformations were divided according to the criteria employed in the work by Lazzeri and coworkers [4], that is:

$$U = \{\mathbf{x} \,|\, Q_{heavy}(\mathbf{x}) \le 0.01\} \tag{S4a}$$
$$F = \{\mathbf{x} \,|\, Q_{heavy}(\mathbf{x}) \ge 0.99\}. \tag{S4b}$$

The intermediate states are neglected in favor of conformations closest to the native folded state and to the completely denaturated state. The number of configurations corresponding to the two basins U and F was large enough to prune the trajectories but not sufficient to have statistically robust results. Therefore, 10 independent downsamplings of those frames belonging to the two states were operated to obtain 10 independent sequences of 10,000 configurations for each state.

S6. K-MEDOIDS CLUSTERING

K-medoids clustering belongs to the class of partitioning clustering algorithms [14, 15]. Differently from analogous methods, the centers of the clusters - the *medoids* - are points that belong to the original dataset, making the algorithm less sensitive to outliers. The algorithm starts with an initialization step where all points are assigned to a predefined number of clusters K and the medoids are identified as the data points closer to the cluster average. The assignment is redefined to minimize *inertia*, that is, the sum of distances of points from their reference medoid; then the new medoids are computed and the process is iterated until convergence.

Here, we used the K-medoids clustering implementation from Sklearn Extra with `k-medoids++` as initial assignment criterion, since it gives starting medoids that are more separated than the default method. Given that the Sklearn algorithm uses square distances, the K-medoids clustering was performed on the MSD distance matrix, that was calculated for IH_1 and IH_{12}; we partitioned the data in the two IHs in $K = 2$ clusters, testing the hypothesis that the folding pathway of chignolin can take one of two routes.

We performed a statistical test to assess the significance of the resulting partition with respect to a random assignment of the medoids. Specifically, for the two IH we performed a single iteration of K-medoids clustering on $10,000$ random samples, then we measured the Z-score of the inertia of the real data against the random data:

$$Z = \frac{I_{obs} - \mu_{rand}}{\sigma_{rand}}. \tag{S5}$$

Eq. S5 yields consistently negative results for both IH_1 and IH_{12}. The null hypothesis (optimal clustering is no better than a random clustering) was rejected with a significance level of $\alpha = 0.05$ only for IH_1.

Table S5: Z-scores and p-values for K-Medoid clustering with $K = 2$ for IH_1 and IH_{12}.

	**IH_1**	**IH_{12}**
**Z-score**	-3.255	-1.110
**p-value**	0.00010	0.05529

S7. MAPPING ENTROPY OPTIMIZATIONS OF CLUSTERED IH_1 AND IH_{12}

Due to the limited sampling in the four clusters, the number of `MC_steps` and `rotmats_period` has been increased for IH_{12} to assure that results were robust enough. General parameters are reported in Table S6, while the parameters specific for IH_1 and IH_{12} are shown in Table S7 and S8, respectively.

Table S6: Set of parameter used for the mapping entropy optimizations of clustered IH_{12} and IH_{13}. The asterisk refers to different frame numbers in the trajectories, along with the cluster number.

**atomnum**	**n_frames**	**cgnum**	**MC steps**	**rotmats period**	**Ncores**	**criterion**	**min nclust**	**max nclust**
93	*	10	*	*	48	2	*	*

Table S7: EXCOGITO parameters for mapping entropy optimizations of the two clusters of IH_1

**ih_1**	`p_0`	`p_1`
**n_frames**	3487	7759
**MC_steps**	2000	2000
**rotmats_period**	400	400
**min_nclust**	7	16
**max_nclust**	70	155

Table S8: EXCOGITO parameters for mapping entropy optimizations of the two clusters of IH_{12}

**ih_12**	idx_0	idx_1
**n_frames**	584	89
**MC_steps**	10,000	10,000
**rotmats_period**	2000	2000
**min_nclust**	2	2
**max_nclust**	12	6

S8. SI: RESULTS

Hereafter we report the figures and the tables for the following quantities and analyses:

- RMSF of the C_α atoms of the whole trajectory (Fig. S1)
- Relevance heatmaps for the optimizations of the trajectory divided into 10 IH, 20 IH, and 30 IH (Fig. S2)
- Mapping entropy optimization of 20 isocommittor hypersurfaces, from 0 to 19 (Fig. S3 and Fig. S4)
- Fraction of hydrogen bonds per frame at each isocommittor hypersurface (Fig. S5)
- Distance maps of the trajectories at each isocommittor hypersurface (Fig. S6 and Fig. S7)
- Heatmap reporting the RMSF of the C_α atoms for the 20 isocommittor hypersurfaces. (Fig. S8)
- Heatmap reporting the fraction of the native contacts per residue of the C_α atoms for the 20 isocommittor hypersurfaces (Fig. S9).
- Explained variance and inverse participation ratio of the trajectories at each isocommittor hypersurface (Fig. S10 and Fig. S11)
- Heatmaps reporting the PCA loadings calculated for the first three principal components at each isocommittor hypersurface. (Fig. S12)
- Free energy projected onto the (d_{3-7}, d_{2-8}) plane for isocommittor hypersurfaces from 0 to 19 (Fig. S13 and Fig. S14) and for the zeroth isocommittor hypersurface (Fig. S15)
- Distribution of d_{3-7} for the IH_1 division into p_0 and p_1 as counts, highlighting the threshold at $d_{3-7} = 0.505$ nm.(Fig. S16)
- MSD and the distance d_{3-7} calculated for the two clusters of IH_1 according to d_{3-7} and IH_{12} obtained with MSD (Fig. S17)
- Analysis of the fraction of native contacts (Fig. S18)
- Measures used to analyze IH_1 (Fig. S19) and IH_{12} (Fig. S20)
- Graphic representation of the crystal structure of CLN025 and the 10 structures generated by AlphaFold (Fig. S21)
- Mean RMSD of the backbone, standard deviation, and the 95% confidence interval of the last equilibration frame measured for wild-type CLN025 and the mutated chignolins against the CLN025 crystal (Table S9).

A. Measures across the isocommittor ensembles

Distance maps The distance maps were calculated between the pairwise residues of chignolin for each IH using the same method that was employed in Section S4, with the difference that in this case the calculation has been performed using the C_α atoms only, not the heavy atoms as in the previous case. The distance maps across the committor for the division in 20 IH are reported in Fig. S6 and Fig. S7. At the beginning of the folding process, the contact maps have the largest distance between the two terminals Tyr1 and Tyr10, but by IH_4 the largest distance shifts to being between the central and the terminal amino acids (Thr6-Tyr1, Glu5-Tyr10). After IH_4, the protein tends to assume a progressively more compact form without changing much conformation, hereby confirming that chignolin reaches a close-to-folded arrangement in an early stage of the folding transition.

RMSF of the C_α atoms RMSF was calculated for the C_α atoms only for each IH by employing the Python library `mdtraj`, the results are reported in Fig. S9. RMSF is very high for the terminal residues Tyr1 and Tyr10 at the beginning of the process, but it progressively lowers as the protein assumes its final, folded conformation. The most consistently fluctuating residues across the whole transition are Tyr1, Tyr10, and Pro4.

Per-residue fraction of native contacts The per-residue fraction of native contacts was calculated by adapting the code used in Section S5 to employ C_α atoms only. Since chignolin is very small, the Best-Hummer parameters were adapted to reflect that, the cutoff to define a native contact ($r^0_{i,}$) was raised from 4.5 Å to 8 Å, with the crystal structure (2RVD) used as a reference. The results are reported in Fig. S9 By construction, the highest contacts fraction is always between the three central amino acids Pro4, Glu5, and Thr6. At the beginning of the folding process, the two halves of chignolin do not interact together, the protein seem to assume a more closed conformation at around IH_4, confirming the data obtained with the distance maps in Figs. S6 and S7. The terminal atoms do not establish persistent contacts until about IH_{14}, after which the protein reaches its final, folded form.

PCA PCA was calculated for the C_α atoms of chignolin at each IH using the same method that was employed in the main text. Additionally, the inverse participation ratio [16] of the residues for each principal component (PC) was calculated as

$$\mathrm{IPR}(\mathbf{v}) = \frac{\sum_{i=1}^{N} |\mathbf{v}_i|^4}{\left(\sum_{i=1}^{N} |\mathbf{v}_i|\right)^2}$$

where $|\mathbf{v}_i|$ is the norm of the cartesian components of the i-th eigenvector. IPR lies between the values of $1/N$, indicating global motions, and 1, which stands for more localized movements. The results of the PCA cumulated variance along with IPR for all IHs are reported in Fig. S10 and Fig. S11. From the two panel we can immediately notice that the cumulated variance for the first PC is always around the values of 0.3-0.4 but it increases towards the end of the transition process up to 0.5-0.6 in IH_{15} and IH_{16}, where we already observed the “settling” of the terminals into the folded position. This means that chignolin, during the process, does not have a preferred direction until it starts forming the final folded structure. The range of IPR is always around 0.2-0.5, indicating that the motions involving the proteins are mostly global during the whole process. This might be due to the small size of chignolin. Additionally, the PCA loadings [17] - how much each amino acid contributes to each PC - were retrieved for the first three PCs, for each IH. These were calculated as

$$L_i = |\mathbf{v}_i|\sqrt{\lambda_i}$$

where λ_i is the i-th eigenvalue corresponding to the i-th eigenvector $\mathbf{v}_i$. The results for L_i are reported in Fig. S12, showing that the largest contibutions to the fluctuations are most remarkably in PC1 (top), and mostly due to Tyr1, Pro4, and Tyr10, reflecting the findings of RMSF in Fig. S8.

[1] Richard J. Gowers, Max Linke, Jonathan Barnoud, Tyler J. E. Reddy, Manuel N. Melo, Sean L. Seyler, Jan Domański, David L. Dotson, Sébastien Buchoux, Ian M. Kenney, and Oliver Beckstein, "MDAnalysis: A Python Package for the Rapid Analysis of Molecular Dynamics Simulations," in *Proceedings of the 15th Python in Science Conference* (Sebastian Benthall and Scott Rostrup, eds.), pp. 98 – 105, 2016.

[2] N. Michaud-Agrawal, E. J. Denning, T. B. Woolf, and O. Beckstein, "Mdanalysis: A toolkit for the analysis of molecular dynamics simulations," *Journal of Computational Chemistry*, vol. **32**, no. 10, pp. 2319–2327, 2011.

[3] R. T. McGibbon, K. A. Beauchamp, M. P. Harrigan, C. Klein, J. M. Swails, C. X. Hernández, C. R. Schwantes, L.-P. Wang, T. J. Lane, and V. S. Pande, "Mdtraj: A modern open library for the analysis of molecular dynamics trajectories," *Biophysical Journal*, vol. 109, no. 8, pp. 1528 – 1532, 2015.

[4] G. Lazzeri, H. Jung, P. G. Bolhuis, and R. Covino, "Molecular free energies, rates, and mechanisms from data-efficient path sampling simulations," *Journal of Chemical Theory and Computation*, vol. **19**, pp. 9060 – 9076, 2023.

[5] K. Lindorff-Larsen, S. Piana, R. O. Dror, and D. E. Shaw, "How fast-folding proteins fold," *Science*, vol. **334**, pp. 517 – 520, 2011.

[6] G. Bussi, D. Donadio, and M. Parrinello, "Canonical sampling through velocity rescaling," *The Journal of Chemical Physics*, vol. **126**, no. 014101, 2007.

[7] S. Honda, T. Akiba, Y. S. Kato, Y. Sawada, M. Sekijima, M. Ishimura, A. Ooishi, H. Watanabe, T. Odahara, and K. Harata, "Crystal structure of a ten-amino acid protein," *Journal of the American Chemical Society*, vol. **130**, pp. 15327 – 15331, 2008.

[8] M. Parrinello and A. Rahman, "Polymorphic transitions in single crystals: A new molecular dynamics method," *Journal of Applied Physics*, vol. **52**, no. 12, pp. 7182 – 7190, 1981.

[9] M. Giulini, R. Fiorentini, L. Tubiana, R. Potestio, and R. Menichetti, "Excogito, an extensible coarse-graining toolbox for the investigation of biomolecules by means of low-resolution representation," *Journal of Chemical Information and Modeling*, vol. **64**, no. 12, pp. 4912 –4927, 2024.

[10] M. Giulini, R. Menichetti, M. Scott Shell, and R. Potestio, "An information-theory-based approach for optimal model reduction of biomolecules," *Journal of Chemical Theory and Computation*, vol. **16**, no. 11, pp. 6795–6813, 2020.

[11] R. Holtzman, M. Giulini, and R. Potestio, "Making sense of complex systems through resolution, relevance, and mapping entropy," *Physical Review E*, vol. 106, no. 4, p. 044101, 2022.

[12] R. Menichetti, M. Giulini, and R. Potestio, "A journey through mapping space: characterising the statistical and metric properties of reduced representations of macromolecules," *The European Physical Journal B*, vol. 94, no. 10, p. 204, 2021.

[13] R. B. Best, G. Hummer, and W. A. Eaton, "Native contacts determine protein folding mechanism in atomistic simulations," *Proceedings of the National Academy of Sciences*, vol. **110**, no. 44, 2013.

[14] B. Keller, X. Daura, and W. F. van Gunsteren, "Comparing geometric and kinetic cluster algorithm for molecular simulation data," *The Journal of Chemical Physics*, vol. **132**, p. 074110, 2010.

[15] H. Park and C. Jun, "A simple and fast algorithm for k-medoids clustering," *Expert Systems with Applications*, vol. **36**, no. 2, pp. 3336 – 3341, 2009.

[16] S. Cocco, R. Monasson, and M. Weigt, "From principal component to direct coupling analysis of coevolution in proteins: low-eigenvalue modes are needed for structure prediction," *PLoS computational biology*, vol. 9, no. 8, p. e1003176, 2013.

[17] C. C. David and D. J. Jacobs, "Principal component analysis: a method for determining the essential dynamics of proteins," in *Protein dynamics: Methods and protocols*, pp. 193–226, Springer, 2013.

[18] G. Lazzeri, P. G. Bolhuis, and R. Covino, "Optimal rejection-free path sampling," *arXiv preprint arXiv:2503.21037*, 2025.

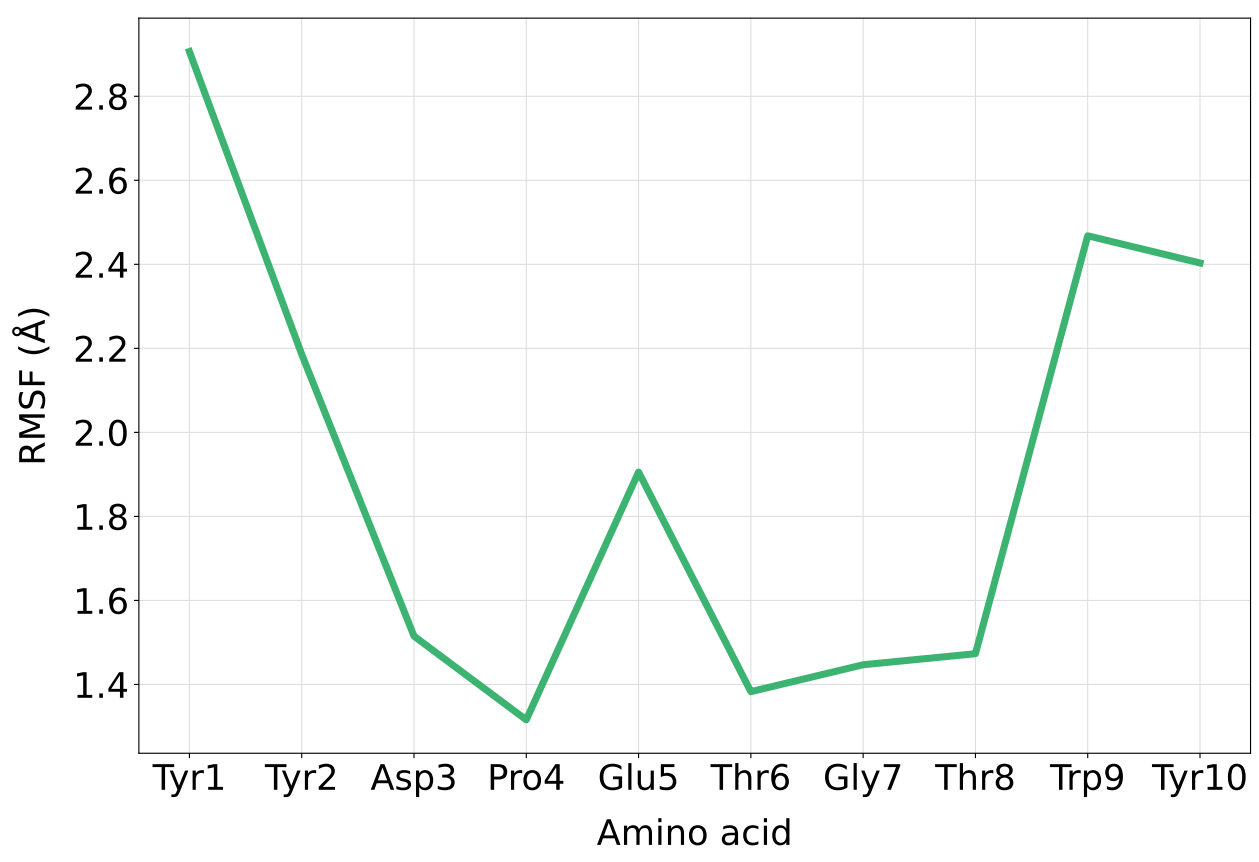

Figure S1: RMSF of the C_α atoms of the whole trajectory.

Table S9: Values of mean RMSD of the backbone, standard deviation, and the 95% confidence interval of the last equilibration frame measured for wild-type CLN025 and the mutated chignolins against the standard crystal structure for CLN025 (PDB: 2RVD).

**mutation**	**mean RMSD (Å)**	**stdev (Å)**	95% **confidence interval (Å)**
CLN025	1.209	0.046	$[1.176, 1.242]$
IH$_1$ Low 2	1.313	0.074	$[1.259, 1, 366]$
IH$_1$ Top 2	1.302	0.113	$[1.221, 1.383]$
IH$_6$ Low 2	1.208	0.087	$[1.145, 1.270]$
IH$_6$ Top 2	1.543	0.128	$[1.451, 1.634]$
IH$_{12}$ Top 2<br>IH$_{19}$ Top 2	1.416	0.060	$[1.373, 1.459]$
IH$_{12}$ Low 2	1.229	0.042	$[1.199, 1, 259]$
IH$_{19}$ Low 2	1.224	0.056	$[1.184, 1.264]$

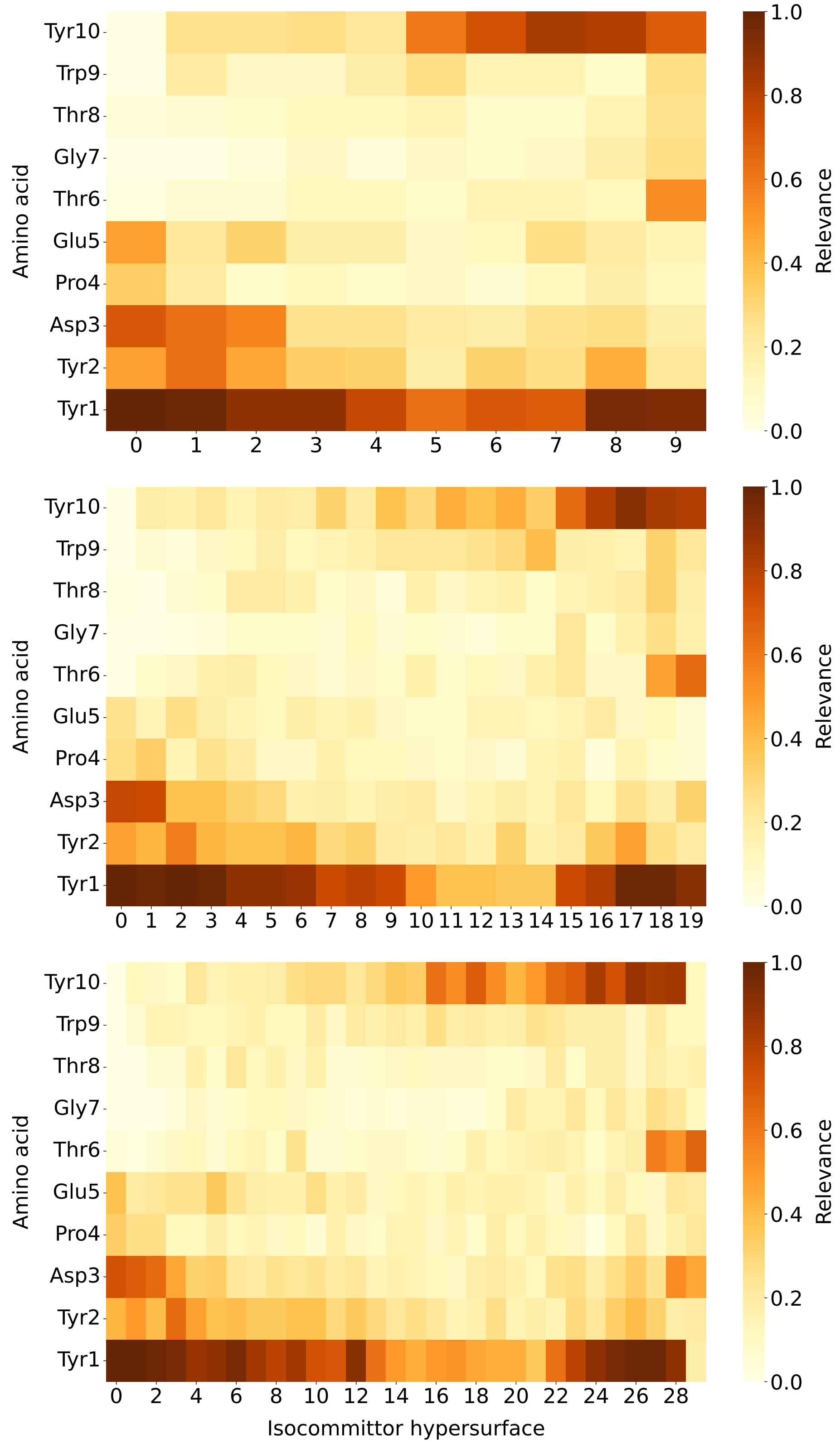

Figure S2: Relevance heatmaps for 10 isocommittor hypersurfaces (top), 20 isocommittor hypersurfaces (center), and 30 isocommittor hypersurfaces (bottom). The coloring scale is the same for all three plots.

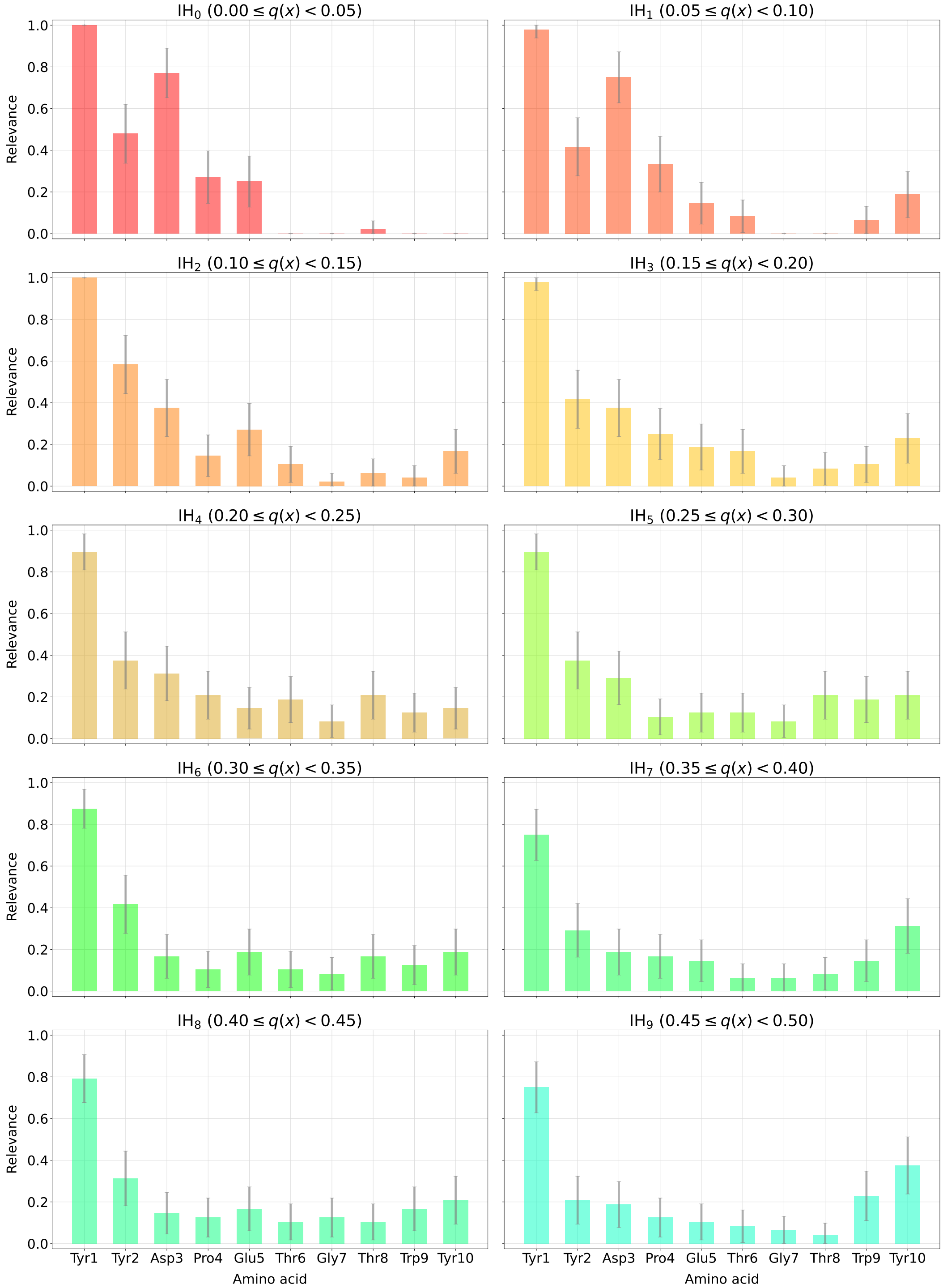

Figure S3: Panel reporting the explicit results with a confidence interval of 95% for the mapping entropy optimization of 20 isocommittor hypersurfaces, from 0 to 9. The plots are ordered from top to bottom and from left to right.

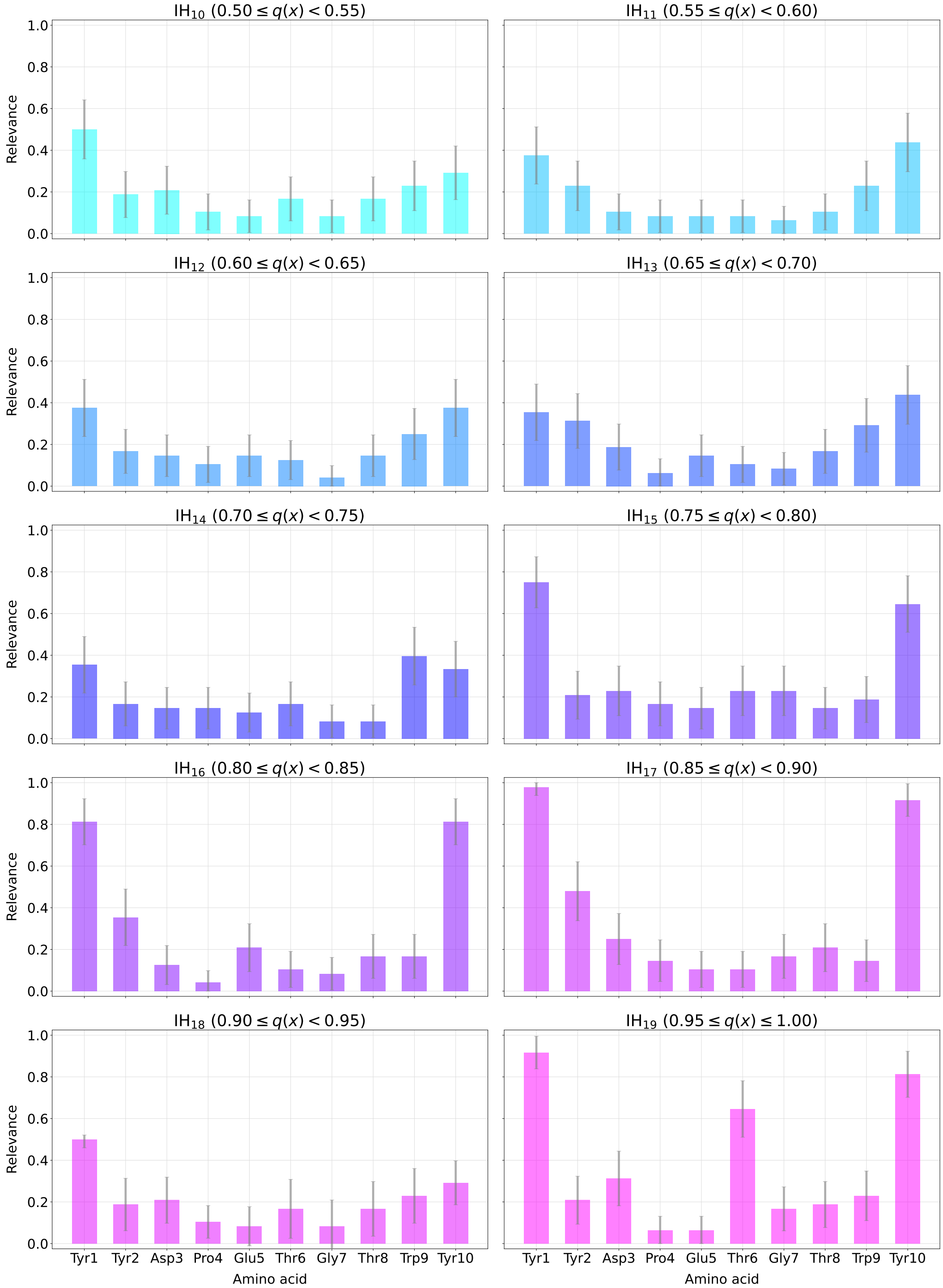

Figure S4: Panel reporting the explicit results with a confidence interval of 95% for the mapping entropy optimization of 20 isocommittor hypersurfaces, from 10 to 19. The plots are ordered from top to bottom and from left to right.

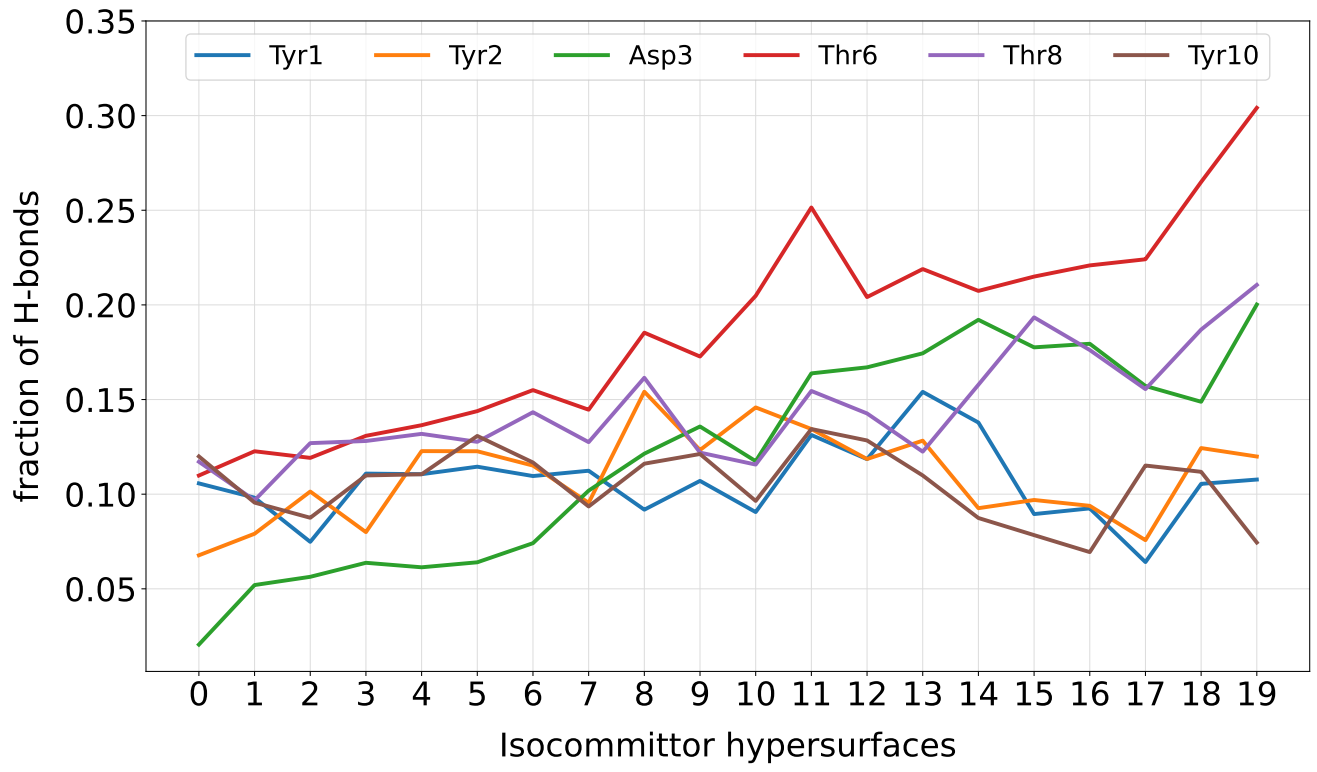

Figure S5: Fraction of hydrogen bonds per frame at each isocommittor hypersurface for those amino acids able to donate or accept a hydrogen bond.

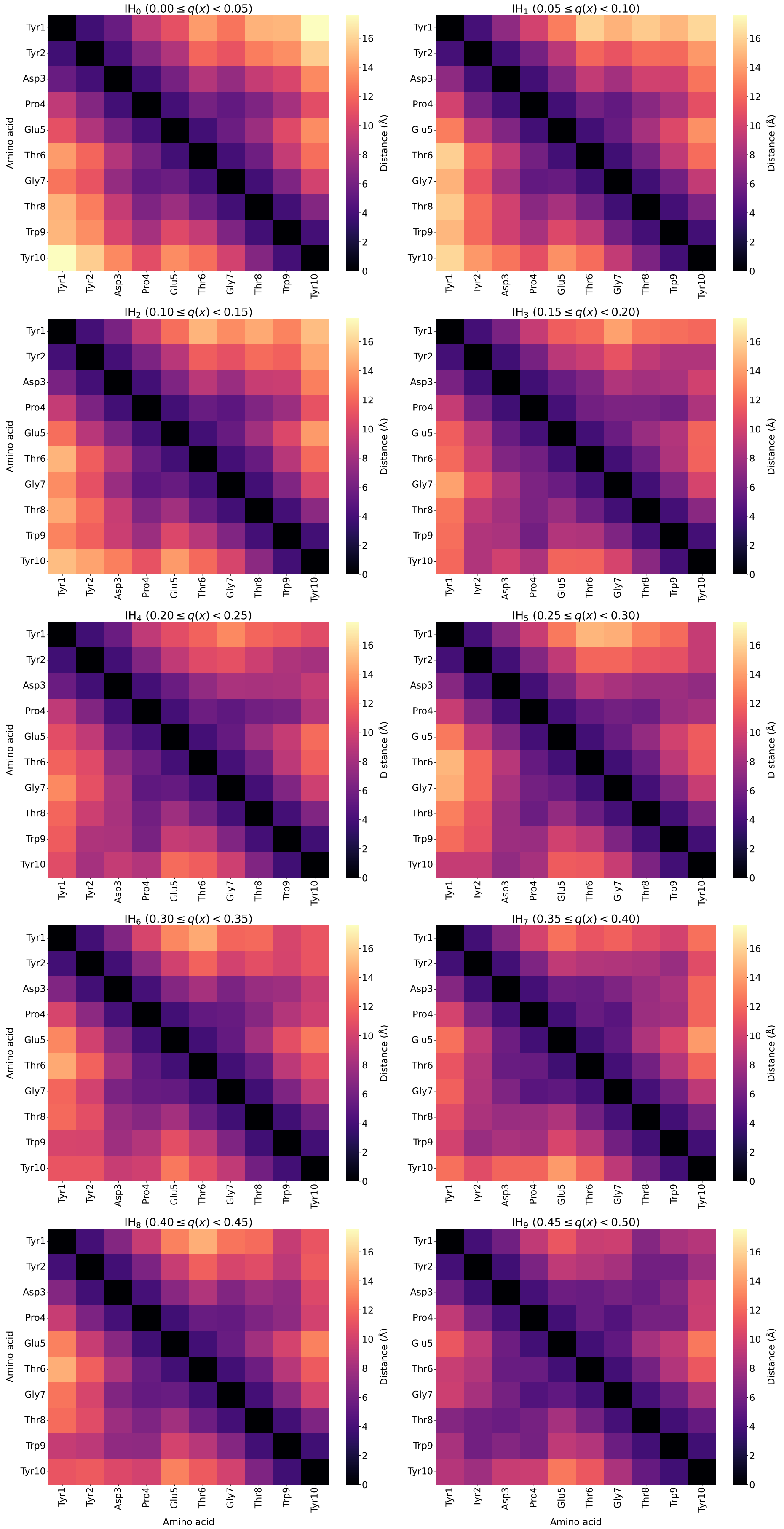

Figure S6: Panel reporting the distance maps between the C_α atoms for the 20 isocommittor hypersurfaces, from 0 to 9. The plots are ordered from top to bottom and from left to right. The coloring scale is the same for all the plots.

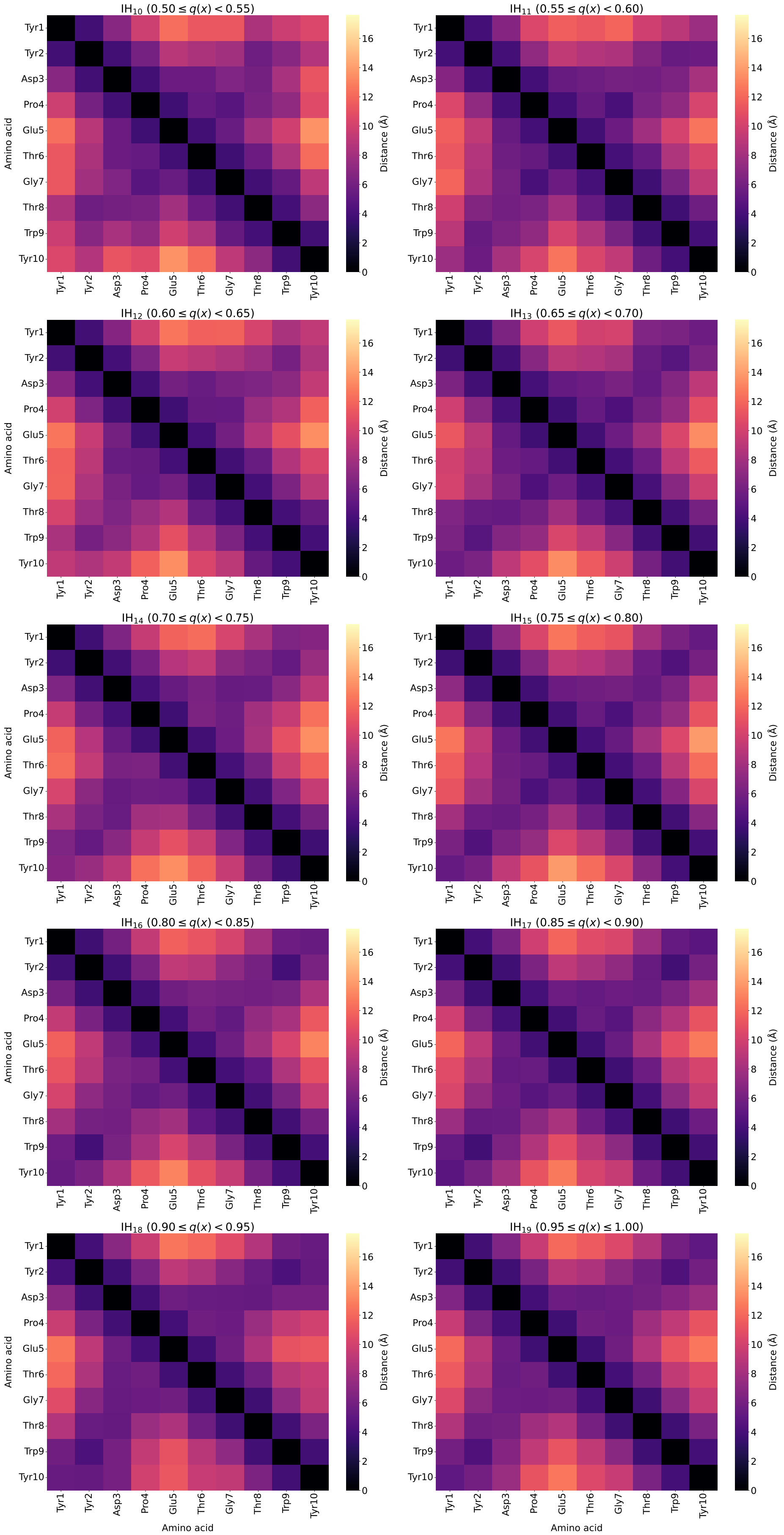

Figure S7: Panel reporting the distance maps between the C_α atoms for the 20 isocommittor hypersurfaces, from 10 to 19. The plots are ordered from top to bottom and from left to right. The coloring scale is the same for all the plots.

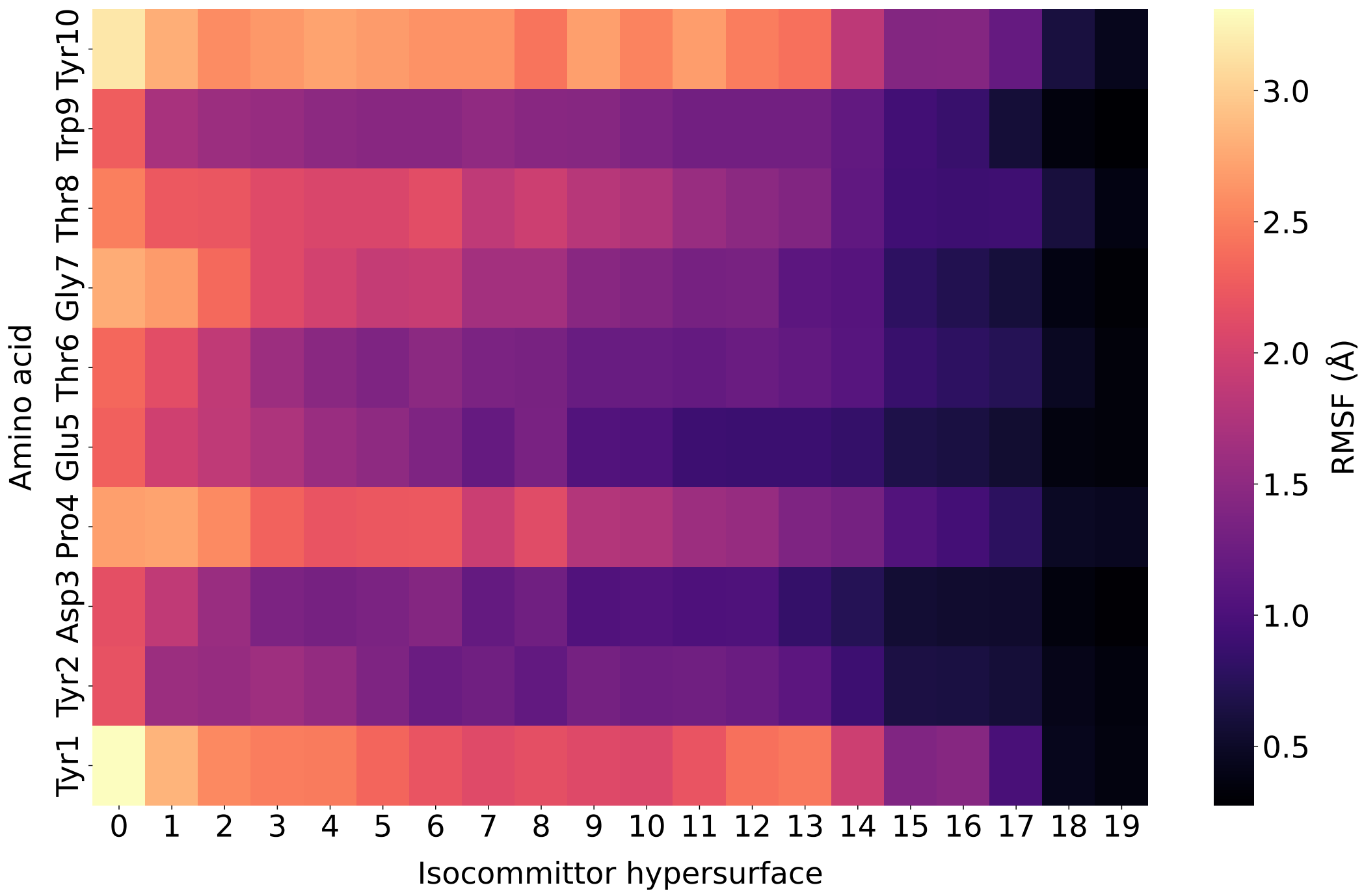

Figure S8: Heatmap reporting the RMSF of the C_α atoms for the 20 isocommittor hypersurfaces.

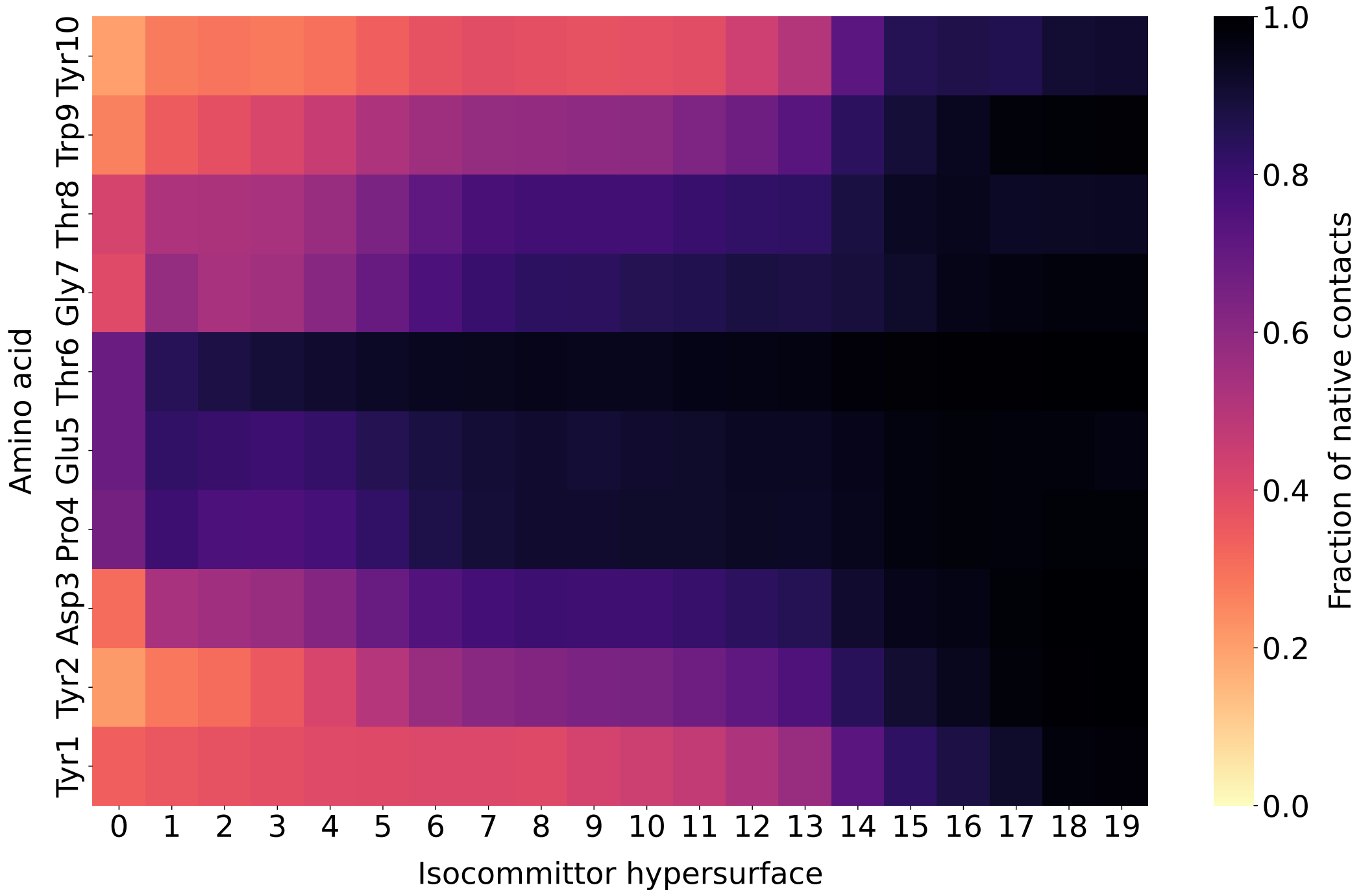

Figure S9: Heatmap reporting the fraction of the native contacts per residue of the C_α atoms for the 20 isocommittor hypersurfaces.

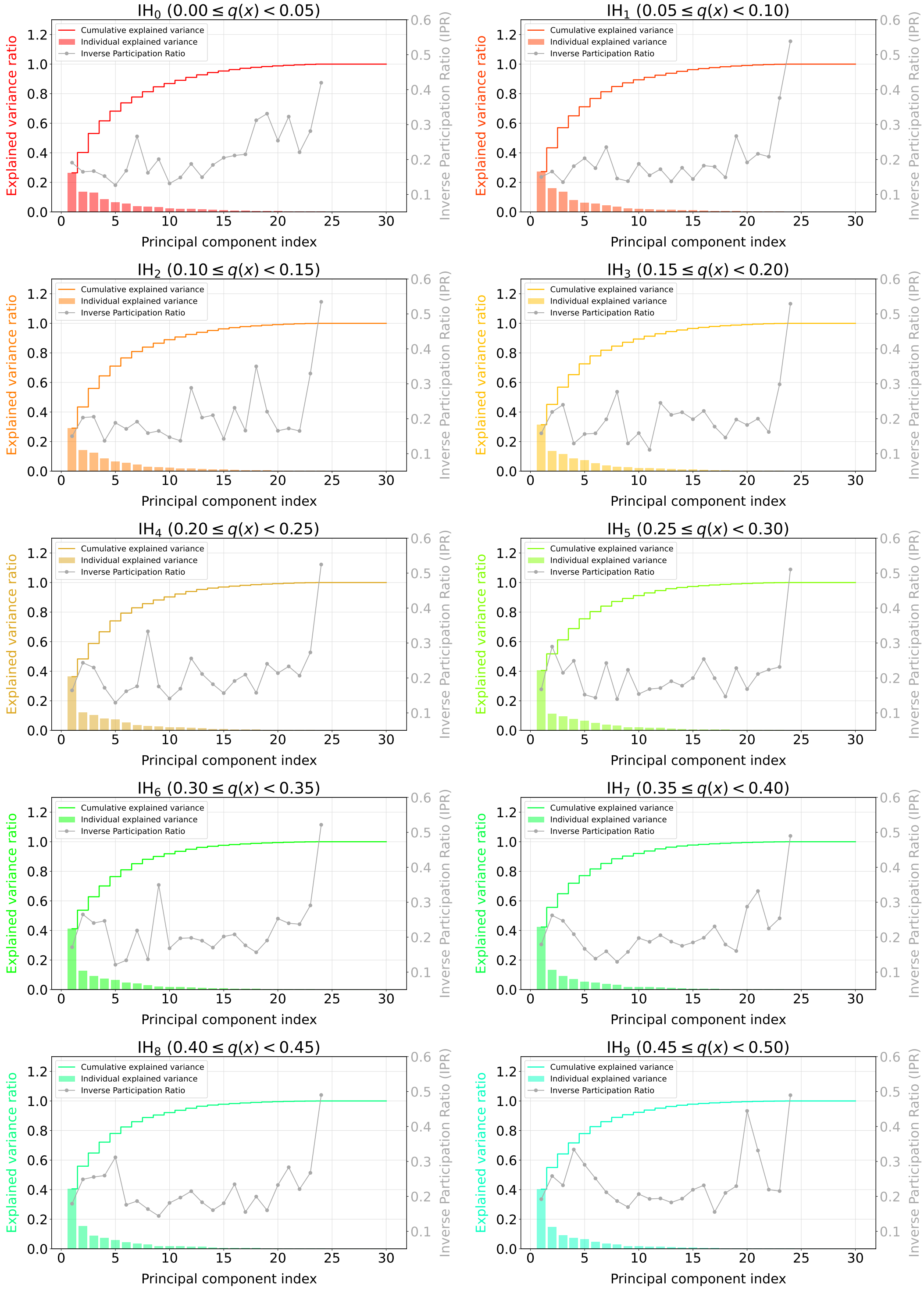

Figure S10: Panel reporting the explained variance for each principal component (histogram, colored), the cumulative explained variance (step, colored), and the inverse participation ratio (line, grey, only for nonzero principal components) for the 20 isocommittor hypersurfaces, from 0 to 9. The plots are ordered from top to bottom and from left to right.

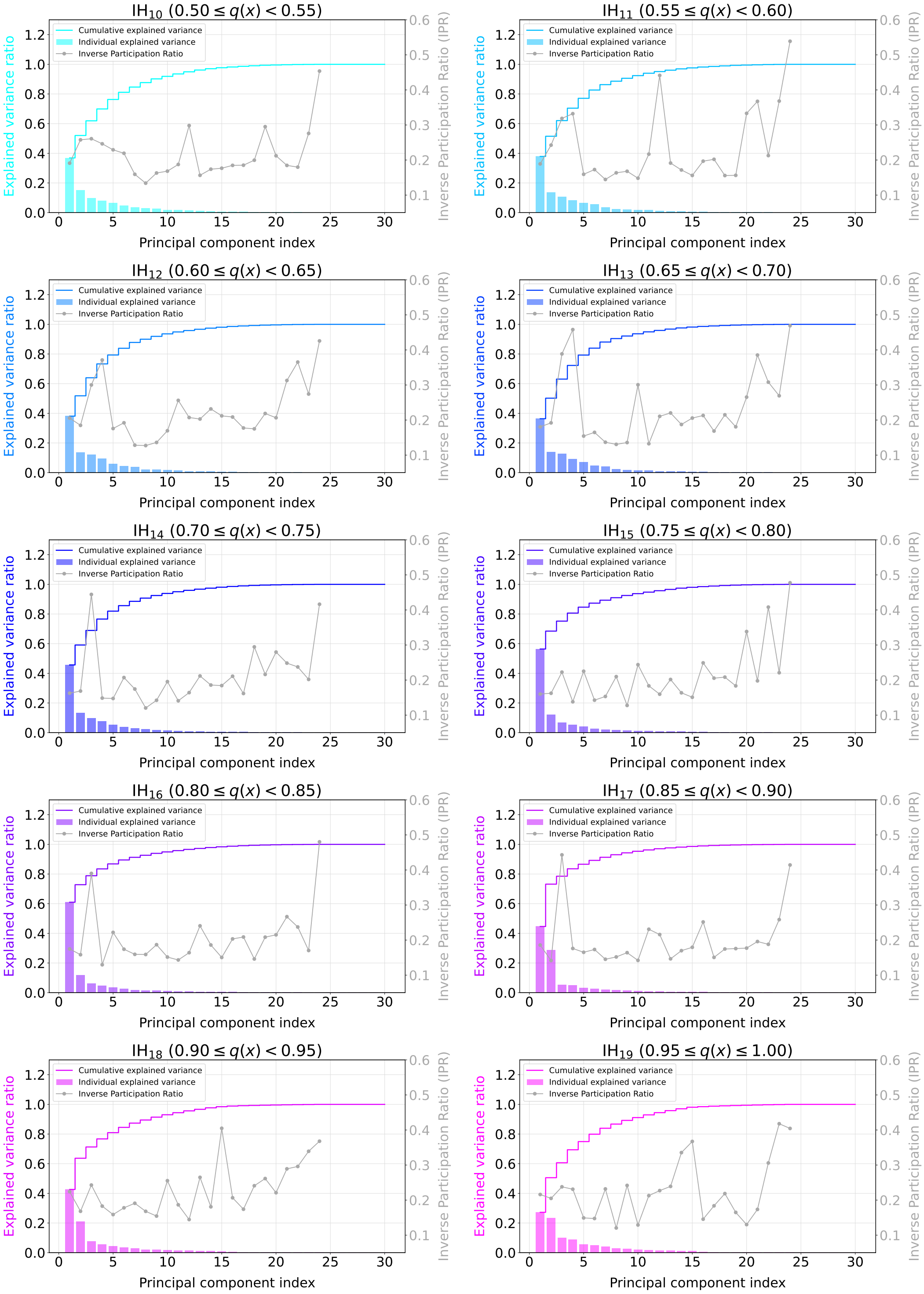

Figure S11: Panel reporting the explained variance for each principal component (histogram, colored), the cumulative explained variance (step, colored), and the inverse participation ratio (line, grey, only for nonzero principal components) for the 20 isocommittor hypersurfaces, from 10 to 19. The plots are ordered from top to bottom and from left to right.

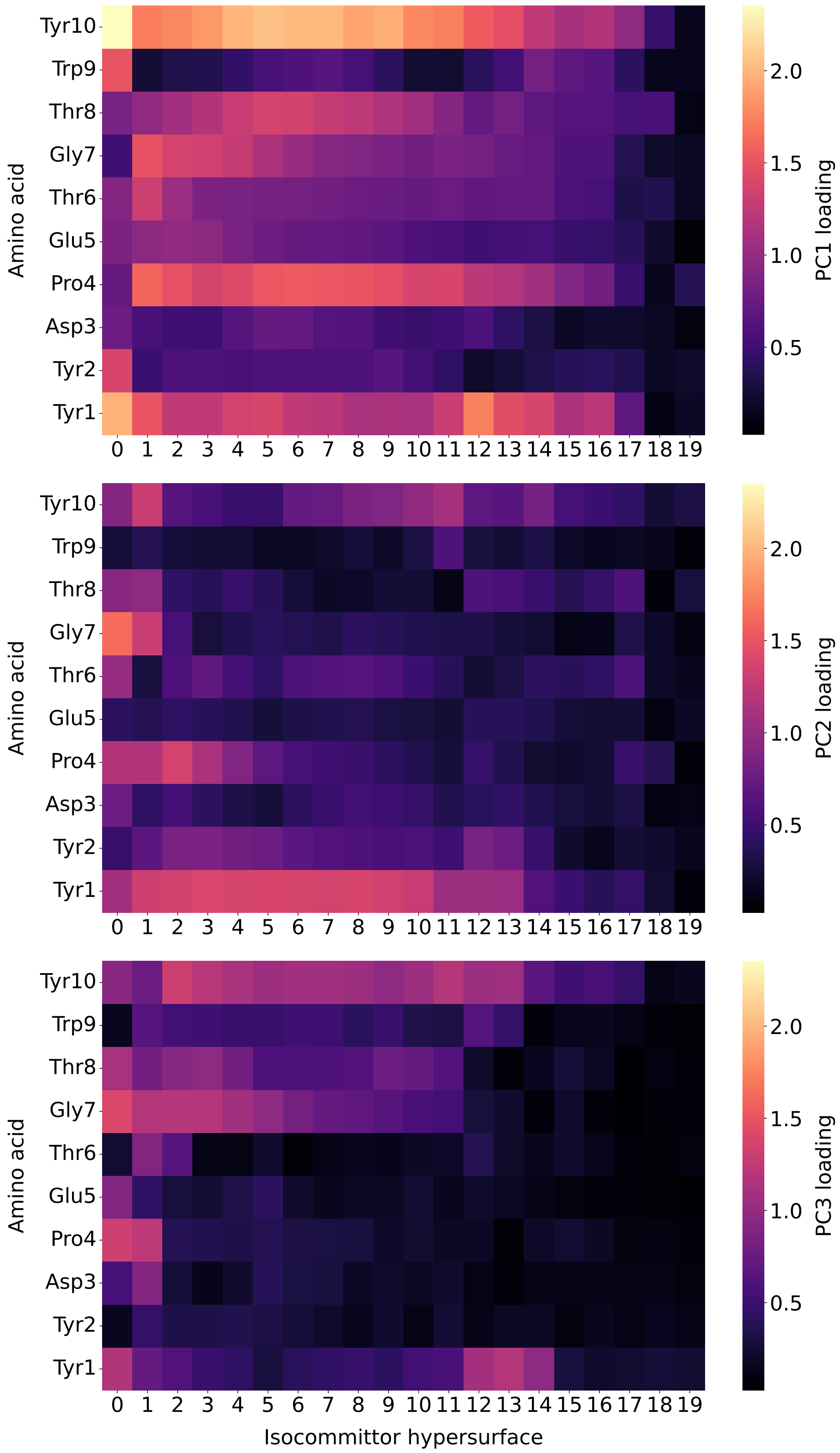

Figure S12: Heatmaps reporting the PCA loadings calculated for the first principal component (top), the second principal component (center), and the third principal component (bottom) at each isocommittor hypersurface. The coloring scale is the same for all three plots.

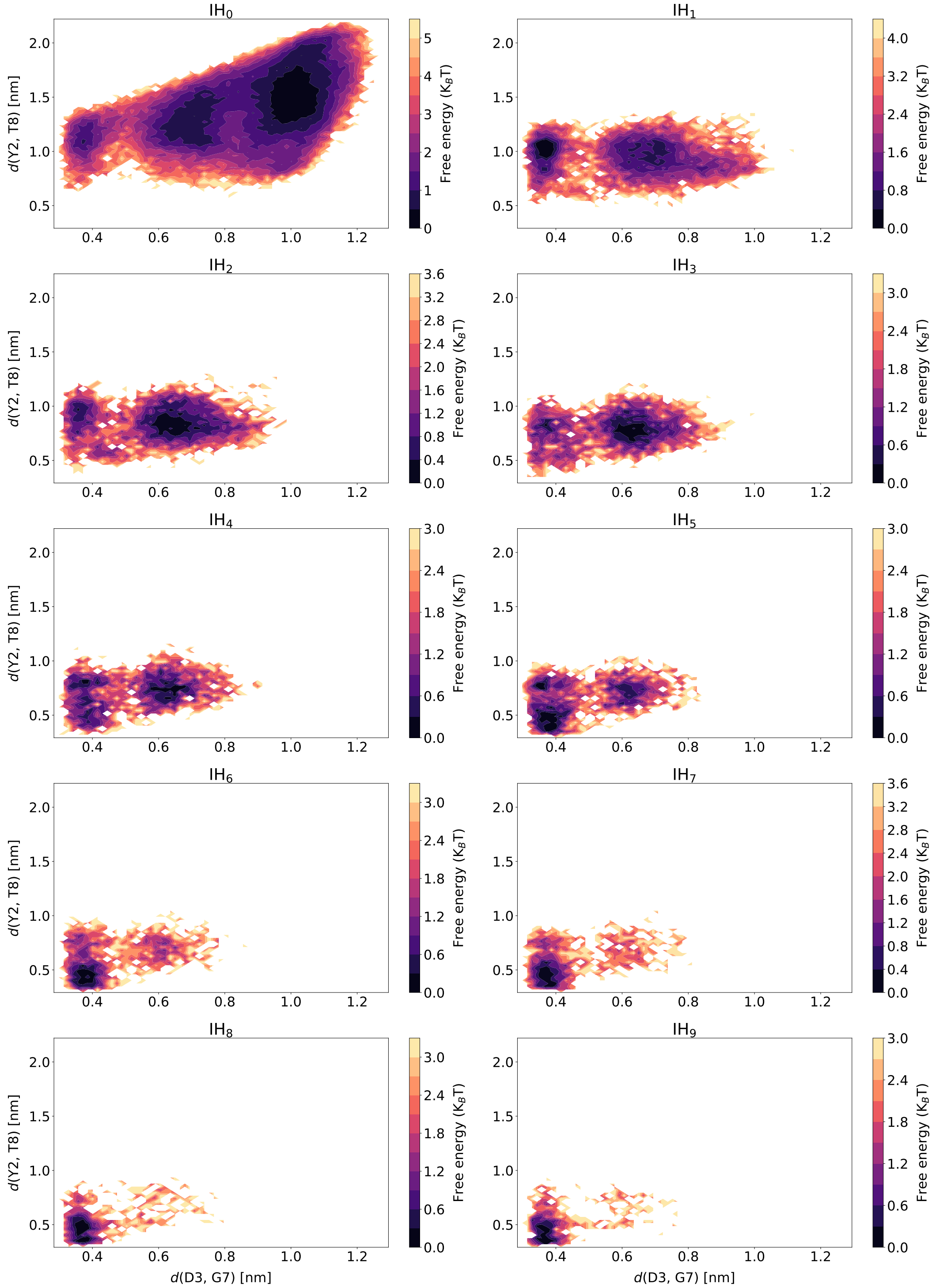

Figure S13: Panel reporting the free energy projected onto the (d_{3-7}, d_{2-8}) plane, calculated according to [18], for isocommittor hypersurfaces from 0 to 9.

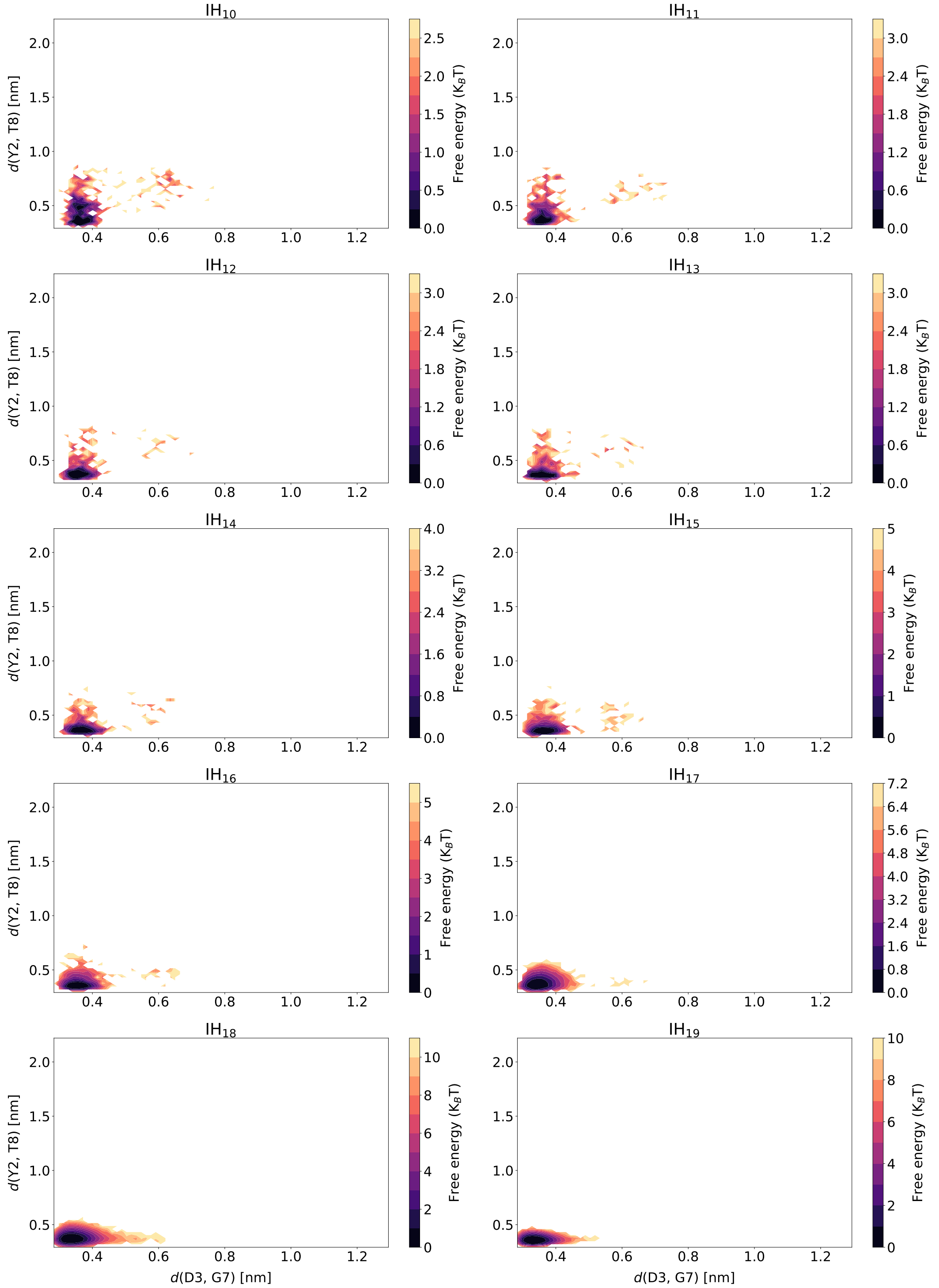

Figure S14: Panel reporting the free energy projected onto the (d_{3-7}, d_{2-8}) plane, calculated according to [18], for isocommittor hypersurfaces from 10 to 19.

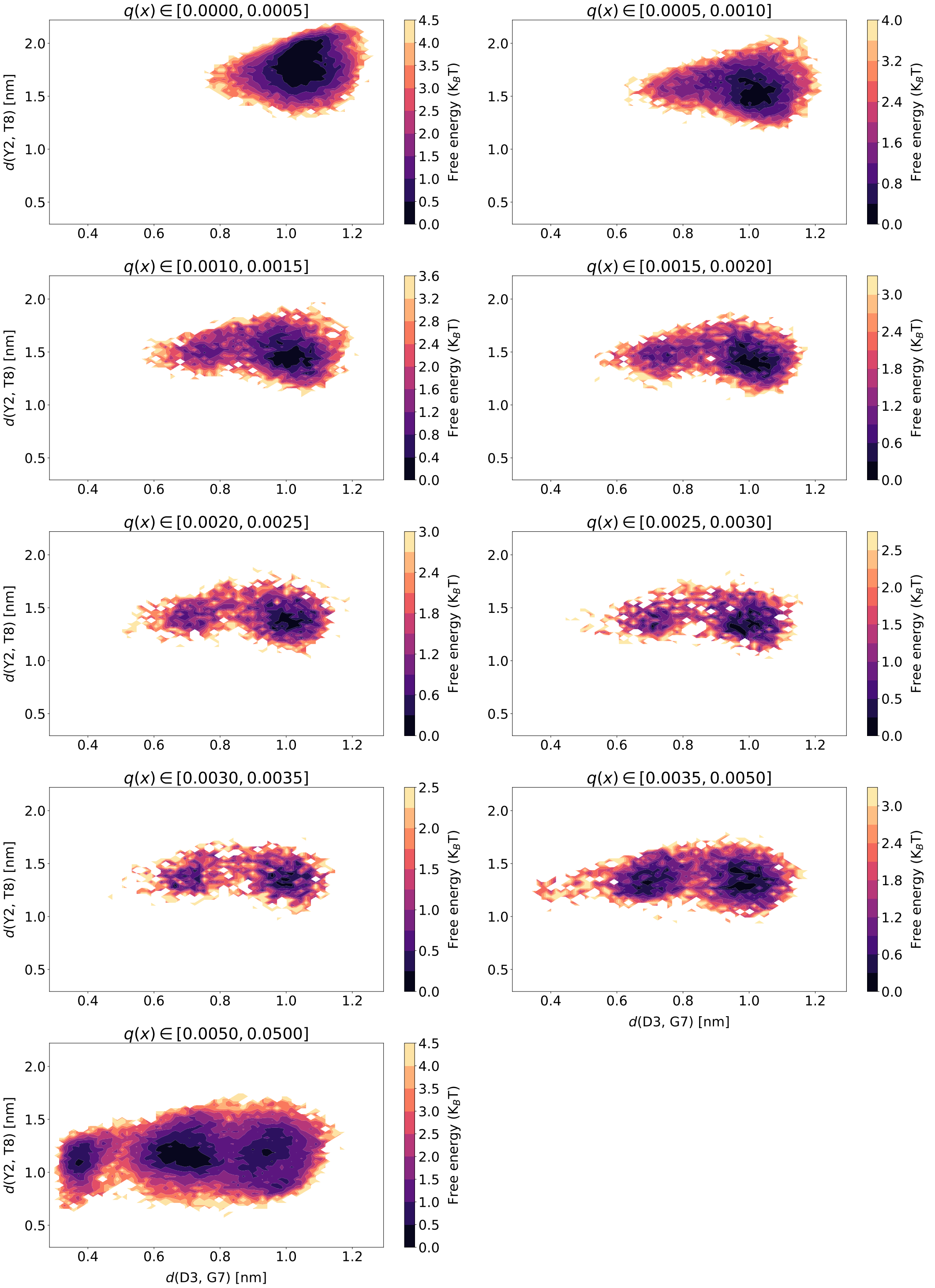

Figure S15: Panel reporting the free energy projected onto the (d_{3-7}, d_{2-8}) plane, calculated according to [18], for the zeroth isocommittor hypersurface (unfolded state). The corresponding committor values $q(x) \in [0.00, 0.05]$ have been divided into inhomogeneous sub-intervals to have a better understanding of the ongoing process.

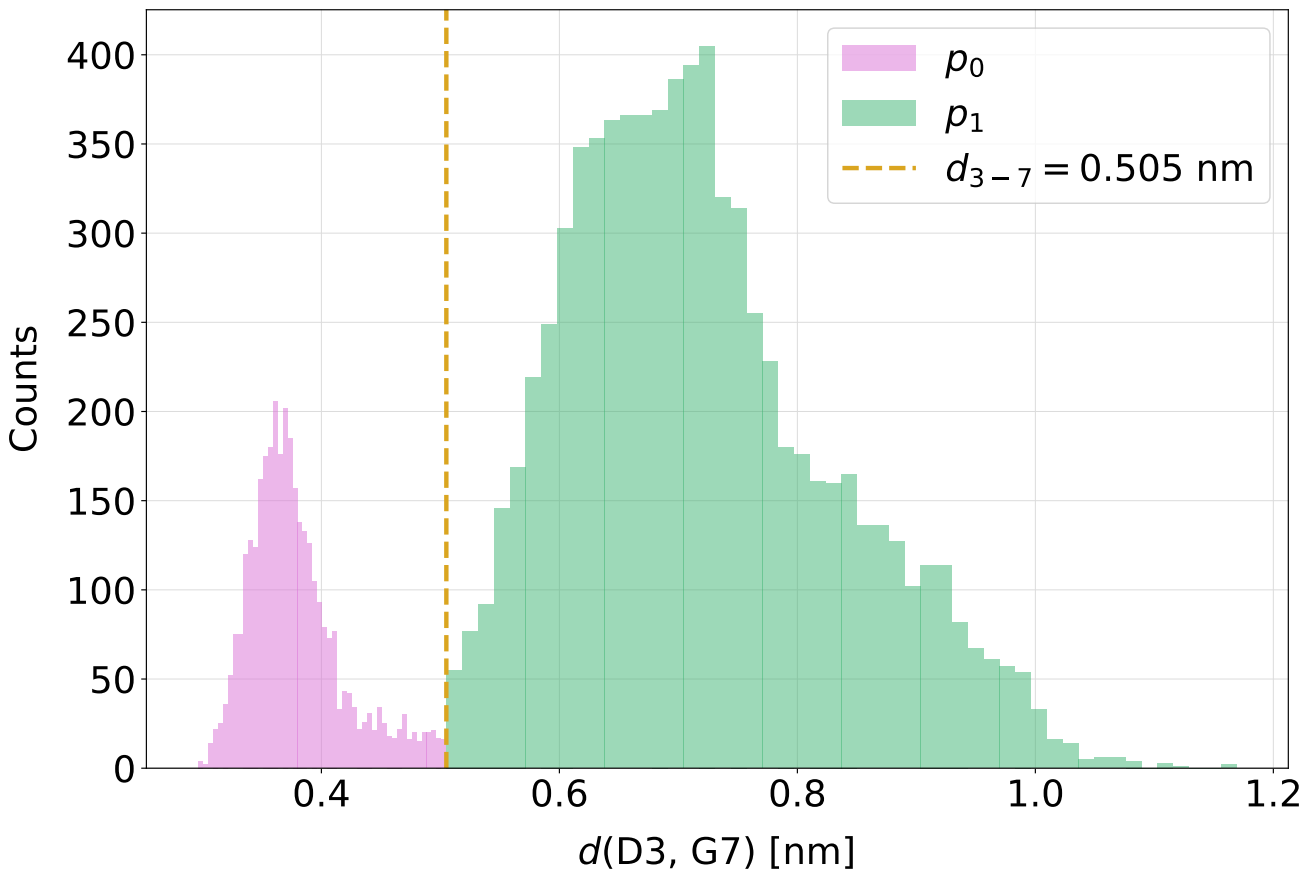

Figure S16: Distribution of d_{3-7} for the IH_1 division into p_0 and p_1 as counts, highlighting the threshold at $d_{3-7} = 0.505$ nm.

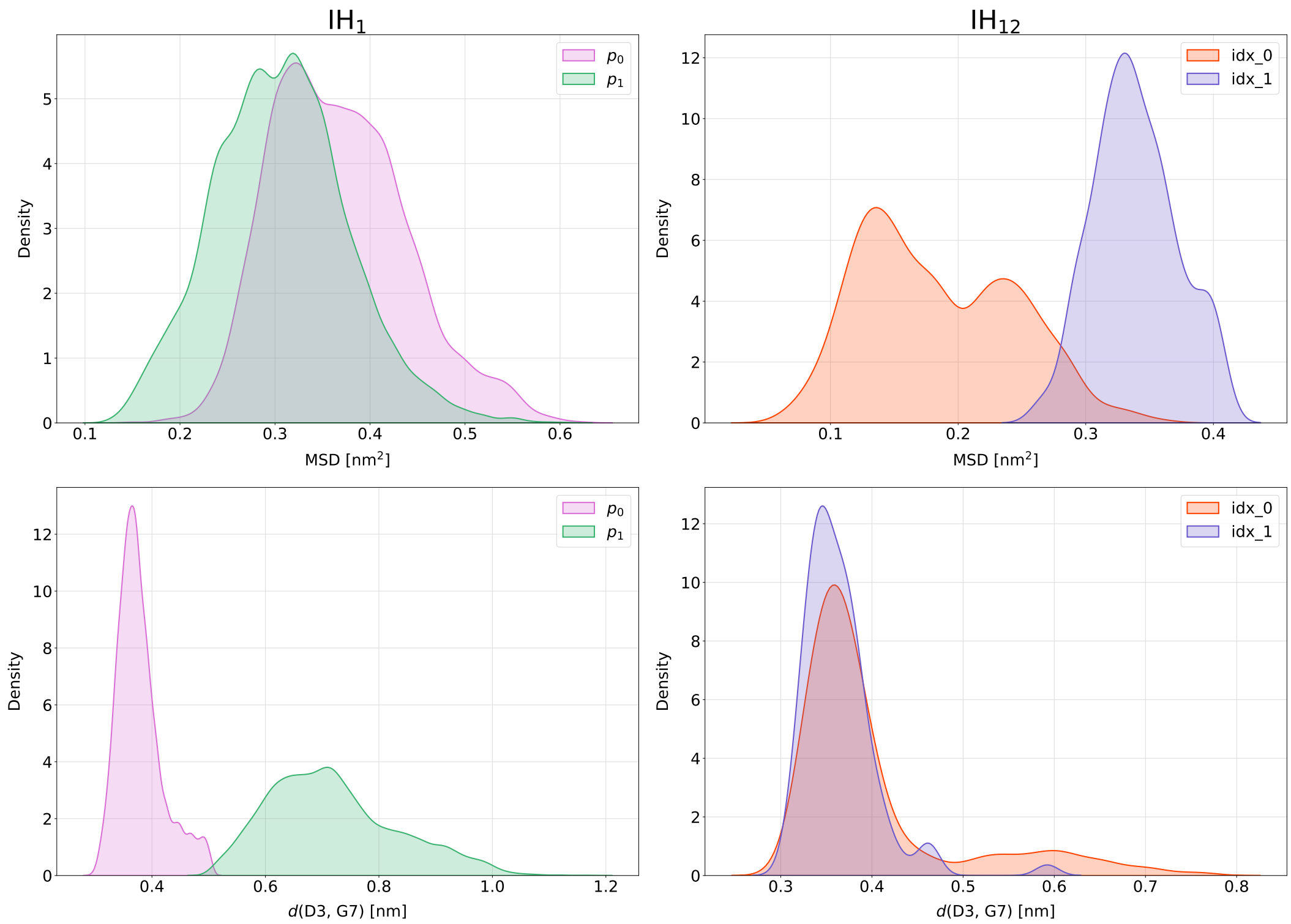

Figure S17: Panel reporting MSD and the distance d_{3-7} calculated for the two clusters of IH_1 according to d_{3-7} and IH_{12}, obtained with MSD.

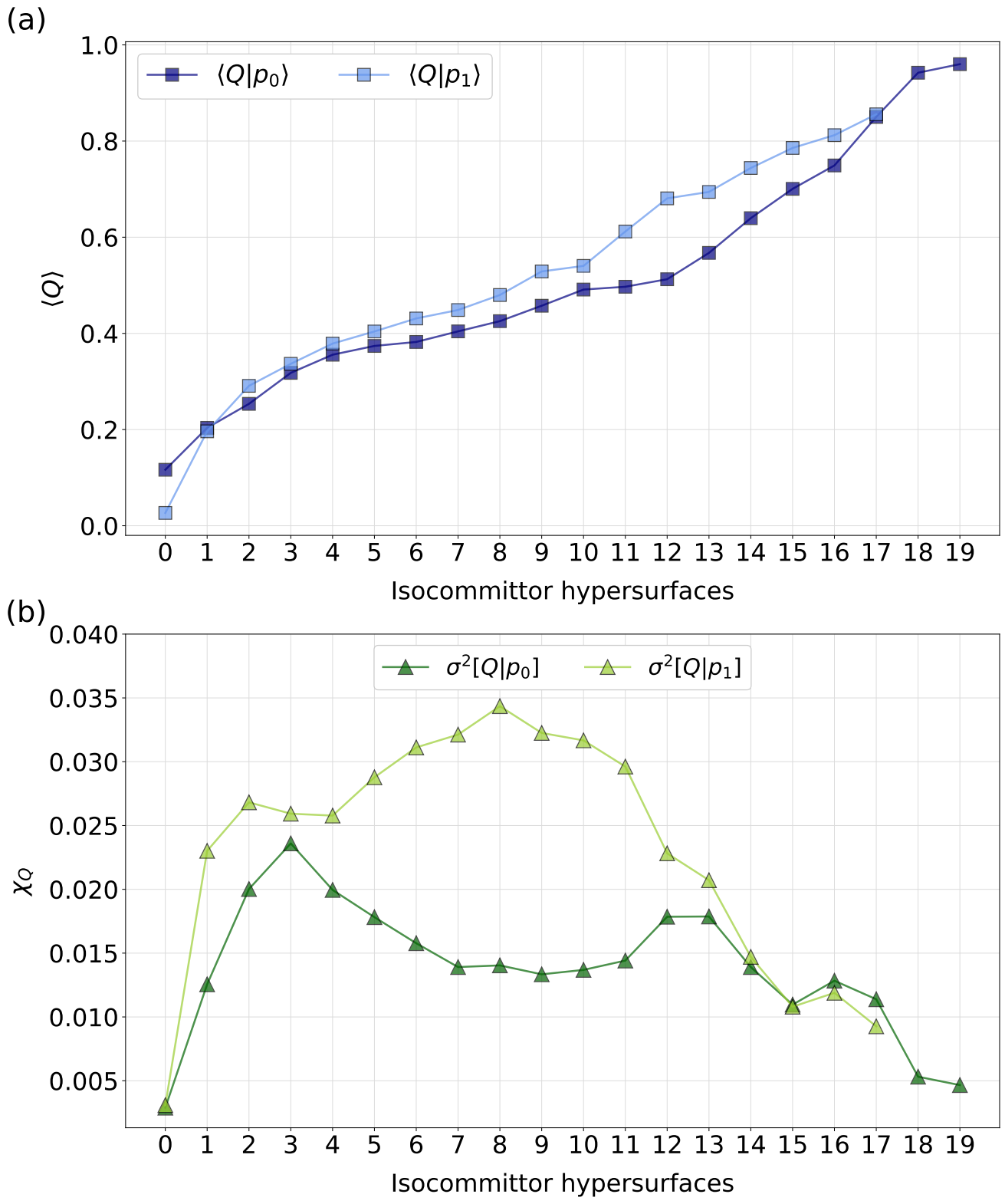

Figure S18: Panel reporting the quantitative analysis of the fraction of native contacts. (a) Average FONC contributions from the sub-states p_0 ($\langle Q|p_0\rangle$) and p_1 ($\langle Q|p_1\rangle$) for each IH, unweighted. (b) Susceptibility χ_Q contributions from the sub-states p_0 ($\sigma^2[Q|p_0]$) and p_1 ($\sigma^2[Q|p_1]$) for each IH.

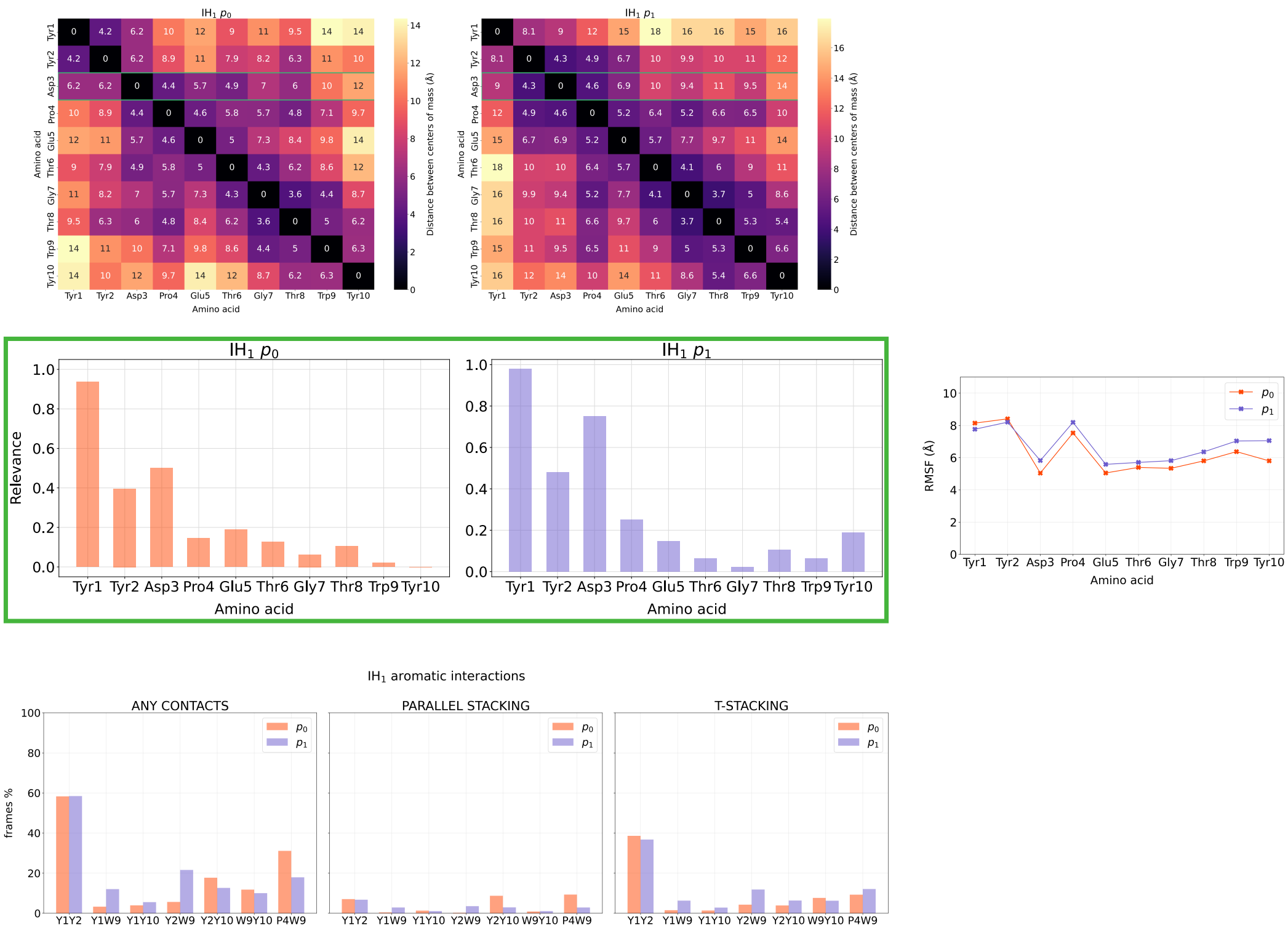

Figure S19: Panel reporting the measures used to analyze IH_1: contact map with all-to-all distances of the centers of mass of amino acids, relevance (MEOW results, highlighted in green), % of frames that contain a specific aromatic interaction among the selected amino acids, d_1, d_2, d_3, d_t, and residue-wise RMSF.

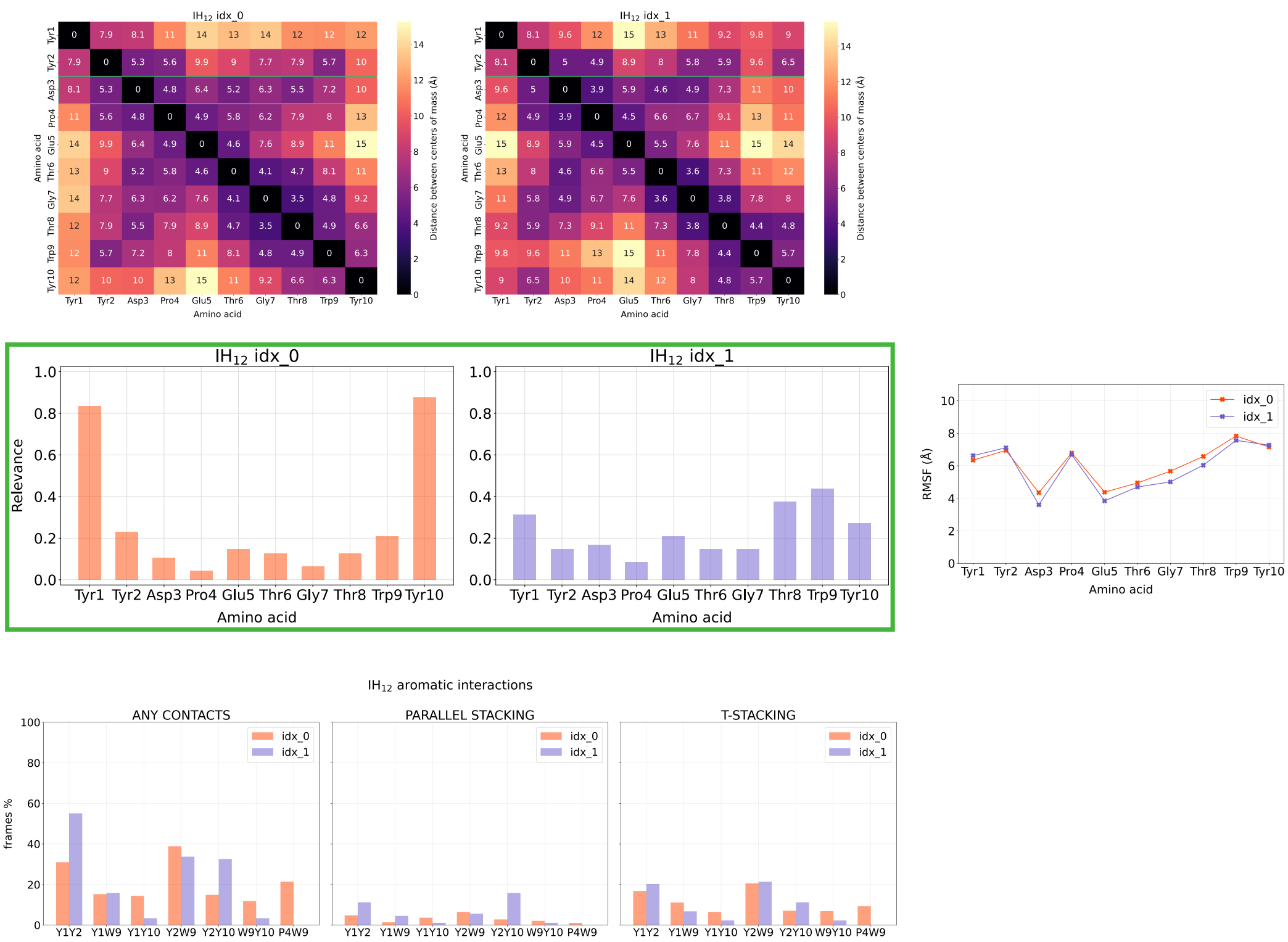

Figure S20: Panel reporting the measures used to analyze IH_{12}: contact map with all-to-all distances of the centers of mass of amino acids, relevance (MEOW results, highlighted in green), % of frames that contain a specific aromatic interaction among the selected amino acids, d_1, d_2, d_3, d_t, and residue-wise RMSF.

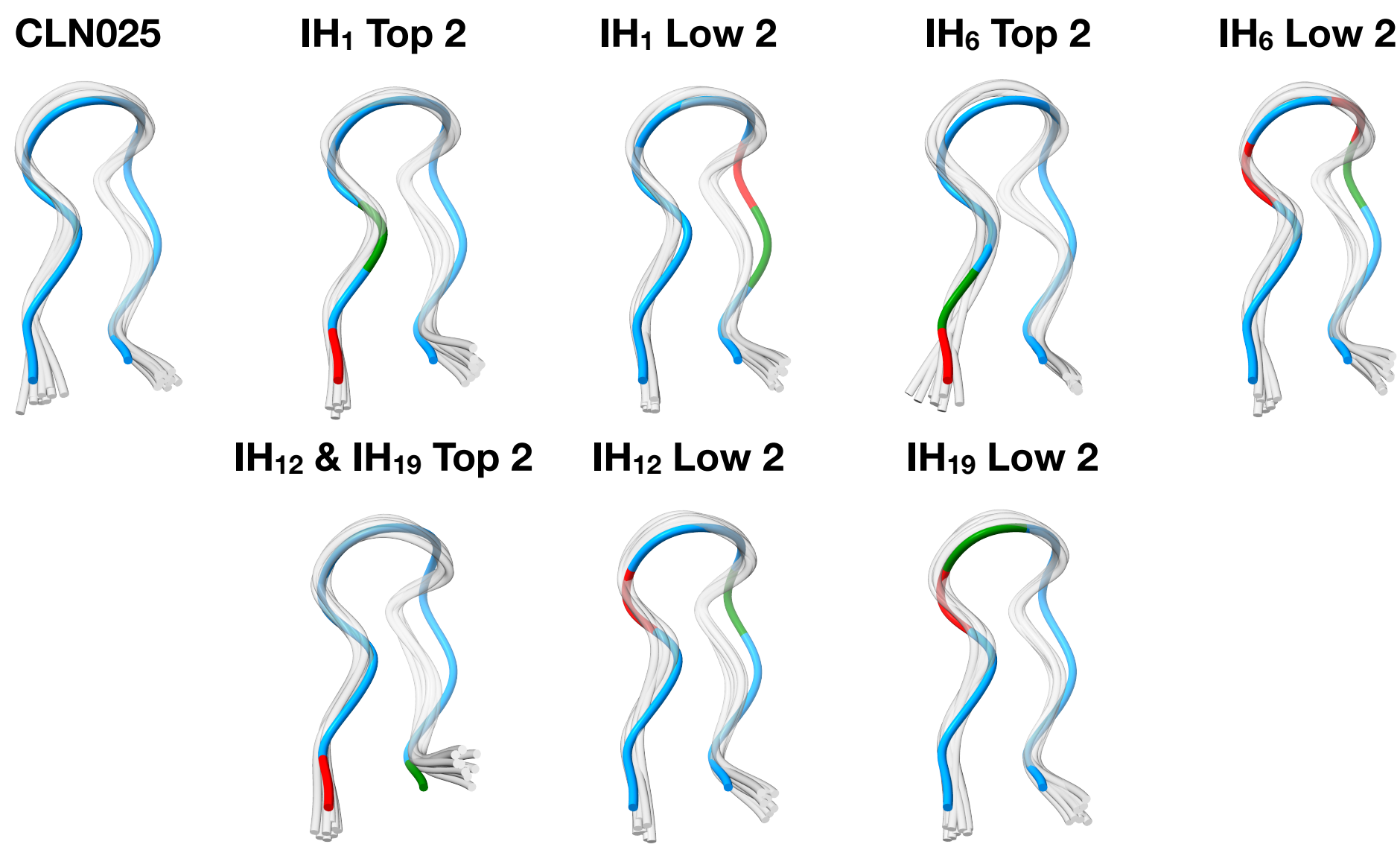

Figure S21: Graphic representation of the crystal structure of CLN025 (in blue) and the 10 structures generated by AlphaFold (silver overlay) with the two mutated residues colored in red and green.
